# Caspase Domain Duplication During the Evolution of Caspase-16

**DOI:** 10.1007/s00239-025-10252-w

**Published:** 2025-05-20

**Authors:** Leopold Eckhart, Attila Placido Sachslehner, Julia Steinbinder, Heinz Fischer

**Affiliations:** 1 Department of Dermatology, Medical University of Vienna, 1090 Vienna, Austria; 2 Division of Cell and Developmental Biology, Center for Anatomy and Cell Biology, Medical University of Vienna, 1090 Vienna, Austria

**Keywords:** Caspase, Protein domain, Pyroptosis, Evolution, Pseudogenization

## Abstract

Caspases are cysteine-dependent aspartate-directed proteases which have critical functions in programmed cell death and inflammation. Their catalytic activity depends on a catalytic dyad of cysteine and histidine within a characteristic protein fold, the so-called caspase domain. Here, we investigated the evolution of caspase-16 (CASP16), an enigmatic member of the caspase family, for which only a partial human gene had been reported previously. The presence of *CASP16* orthologs in placental mammals, marsupials and monotremes suggests that caspase-16 originated prior to the divergence of the main phylogenetic clades of mammals. Caspase-16 proteins of various species contain a carboxy-terminal caspase domain and an amino-terminal prodomain predicted to fold into a caspase domain-like structure, which is a unique feature among caspases known so far. Comparative sequence analysis indicates that the prodomain of caspase-16 has evolved by the duplication of exons encoding the caspase domain, whereby the catalytic site was lost in the amino-terminal domain and conserved in the carboxy-terminal domain of caspase-16. The murine and human orthologs of *CASP16* contain frameshift mutations and therefore represent pseudogenes (*CASP16P*). *CASP16* of the chimpanzee displays more than 98% nucleotide sequence identity with the human *CASP16P* gene but, like *CASP16* genes of other primates, has an intact protein coding sequence. We conclude that caspase-16 structurally differs from other mammalian caspases, and the pseudogenization of *CASP16* distinguishes humans from their phylogenetically closest relatives.

**Supplementary Information:**

The online version contains supplementary material available at 10.1007/s00239-025-10252-w.

## Introduction

Caspases (cysteine-dependent aspartate-directed proteases) are the main regulators of two modes of programmed cell death, apoptosis and pyroptosis (Salvesen and Ashkenazi 2011; Van Opdenbosch and Lamkanfi 2019; Green 2022; Kayagaki et al. 2024; Svandova et al. 2024; Abdelghany et al. 2024). Human caspases-1, -4 and -5 induce pyroptosis and activate pro-inflammatory signaling through cleaving the proforms of interleukin (IL)−1β and IL-18 (Bibo-Verdugo and Salvesen 2024; Newton et al. 2024). Thereby, these caspases play central roles in the immune defense against pathogens (Jorgensen and Miao 2015; Man et al. 2017; Kuriakose and Kanneganti 2023). Capases-2, −8, −9 and −10 induce apoptosis upon oligomerization in protein complexes, leading to the proteolytic activation of caspases-3, -6 and -7. The latter execute a cellular destruction program by cleaving a broad range of protein substrates (Newton et al. 2024; Julien and Wells 2017). Caspase-14 is activated during terminal differentiation of epidermal keratinocytes (Lippens et al. 2000; Eckhart et al. 2000; Fischer et al. 2005), contributes to the proteolytic breakdown of the cornification-associated protein filaggrin (Denecker et al. 2007; Hoste et al. 2011) and is hypothesized to target additional, yet unknown substrates (Steinbinder et al. 2024). The characteristic feature of caspases is the caspase domain (pfam00656: peptidase_C14) (Mistry et al. 2021), which mediates the proteolytic activity. The caspase domain is either preceded by a prodomain which folds into a caspase recruitment domain (CARD) in caspase-1, −2, −4, −5 and −9, or two death effector domains (DED) in caspase-8 and −10, or lacks a defined fold in human caspases with short prodomains (caspase-3, −6, −7, −14). Upon activation, the caspase domain is cleaved at one or two sites to generate a small and a large catalytic subunit. Further proteolytic processing separates the prodomain from the large subunit (Fuentes-Prior and Salvesen 2004). The dysregulation of caspase-dependent inflammation and cell death is a feature of many diseases, leading to attempts to pharmacologically target caspases. However, the incomplete understanding of caspase functions in humans and animal models has hampered the development of effective and safe modulators of caspase activity (Dhani et al. 2021).

Several mammalian caspases have not been fully characterized so far and their roles in the evolution of humans are only incompletely known. Caspase-11 and −13 have been recognized as the respective murine and bovine ortholog of human caspase-4 (Wang et al. 1998; Humke et al. 1998, Koenig et al. 2001; Eckhart and Fischer 2024). Caspase-12 was initially implicated in the control of pyroptosis and endoplasmic reticulum stress-induced cell death, but these roles were not confirmed in later studies (Saleh et al. 2004; Salvamoser et al. 2019). Humans have lost catalytically active caspase-12 due to the mutation of a site critical for activity and, in most populations, due to an additional premature stop codon (Fischer et al. 2002; Puente et al. 2005; Holland et al. 2024). Interestingly, the next relative of humans, the chimpanzee, has a *CASP12* gene with an apparently intact sequence, suggesting that the set of caspases differs among these species (Puente et al. 2005). Caspases-15, −17 and −18 were present in the last common ancestor of mammals and have been lost during the evolution of several mammalian subclades, such as primates including humans (Eckhart et al. 2005, 2006, 2008). Caspase-16, encoded by the gene *CASP16*, was identified as a caspase-14-like protease in various mammals (Eckhart et al. 2008; Sakamaki and Satou 2009). However, the gene structure of the human *CASP16* ortholog, a gene in proximity to the familial Mediterranean fever locus (Centola et al. 1998), has remained incompletely defined with some papers stating that it might be a pseudogene (Eckhart et al. 2008; Sakamaki and Satou 2009). Recent reviews have included caspase-16 as part of the human repertoire of caspases, but it has remained unknown whether caspase-16 is expressed as an active protease in humans (Abdelghany et al. 2024; Svandova et al. 2024).

Here, we investigated the evolution of *CASP16* in the phylogenetic lineages leading to humans, closely related primates and other mammals. Based on the identification and analysis of orthologous genes, we infer the evolution of the primary protein structure of caspase-16 and develop a scenario for the evolutionary loss of *CASP16* in humans.

## Results

### *CASP16* is Conserved in Phylogenetically Diverse Mammals

**Fig. 1:**
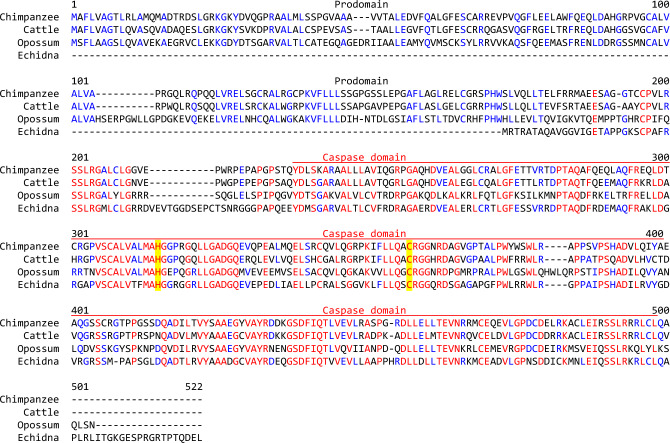
Caspase-16 contains a catalytic site that is conserved in species of the main clades of mammals. Amino acid sequence alignment of caspase-16 proteins of the chimpanzee (*Pan troglodytes*) (GenBank accession number: XP_523278.4), cattle (*Bos taurus*) (XP_005224700.2), opossum (*Monodelphis domestica*) (XP_016279605.1) and echidna (*Tachyglossus aculeatus*). The caspase domain is indicated by a red line above the sequences. Residues conserved in all or more than half of the sequences are highlighted by red and blue fonts, respectively. Yellow shading marks histidine and cysteine of the catalytic dyad (Color figure online)

*CASP16* genes were identified in the genomes of amniotes by inferring orthology on the basis of best reciprocal sequence similarity, clustering in molecular phylogenetics and conserved local synteny. In agreement with the later criterion, all *CASP16* were flanked by *ZNF213* on the 5’-side. Notably, *CASP16* genes of species from all main mammalian clades, i.e. monotremes (echidna), marsupials (opossum), placental mammals (cattle, chimpanzee) (Fig. 1; Suppl. Fig. S1), encode proteins in which key features of caspases are conserved (see below). Importantly, the protein-coding sequence of *CASP16* is conserved in a large set of phylogenetically diverse mammalian species (Suppl. Fig. S2), but not in all species of mammals. Mutations disrupting the coding sequence are present in the platypus (Suppl. Fig. S3), mouse (Suppl. Fig. S4), guinea pig (Eckhart et al. 2008), cetaceans (Strasser et al. 2015), humans (see below) and perhaps others. Amino acid sequence alignments revealed the conservation of the prodomain in caspase-16 proteins of marsupials and placental mammals, but not in the echidna, and conservation of the catalytic domain in all caspase-16 proteins predicted (Fig. 1, red line; Suppl. Fig. S2B). The residues critical for enzymatic activity, corresponding to Histidine 290 and Cysteine 332 of chimpanzee caspase-16, were strictly conserved (Fig. 1; Suppl. Fig. S2B).

**Fig. 2:**
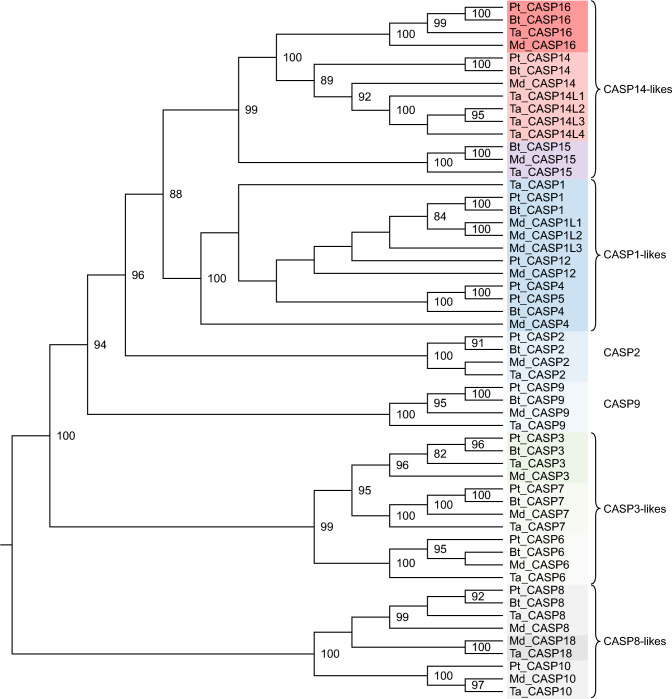
Caspase-16 is phylogenetically closely related to caspase-14. The caspases (CASPs) of chimpanzee (*Pan troglodytes*, Pt), cattle (*Bos taurus*, Bt), opossum (*Monodelphis domestica*, Md) and echidna (*Tachyglossus aculeatus*, Ta) were subjected to maximum likelihood analysis based on the amino acid sequence alignment of the caspase domain. Bootstrap values above 80 are shown on the cladogram. Paralogs of CASP14 of the echidna are named CASP14-like (CASP14L) 1 through 4, and paralogs of CASP1 of the opossum are named CASP1-like (CASP1L) 1 through 3. On the right, the main clades of caspases are labelled according to the best characterized member of each clade (e.g. CASP1-likes)

To determine the phylogenetic position of caspase-16 within the caspase family of proteases, we performed a maximum likelihood analysis of caspases from chimpanzee, cattle, opossum, and echidna. The resulting phylogenetic tree confirmed that the mammalian caspase-16 proteins identified above are orthologous (Fig. 2, Suppl. Fig. S5). In line with data published in previous studies (Eckhart et al. 2008; Sakamaki and Satou 2009), molecular phylogenetics suggested that caspase-16 proteins are most closely related to caspase-14. Caspase-14, −15 and −16 form a monophyletic clade (“CASP14-likes”), which is the sister group of CASP1-likes (Fig. 2, Suppl. Fig. S6).

### The Prodomain of Caspase-16 has Evolved by Duplication of Exons Encoding the Caspase Domain

**Fig. 3:**
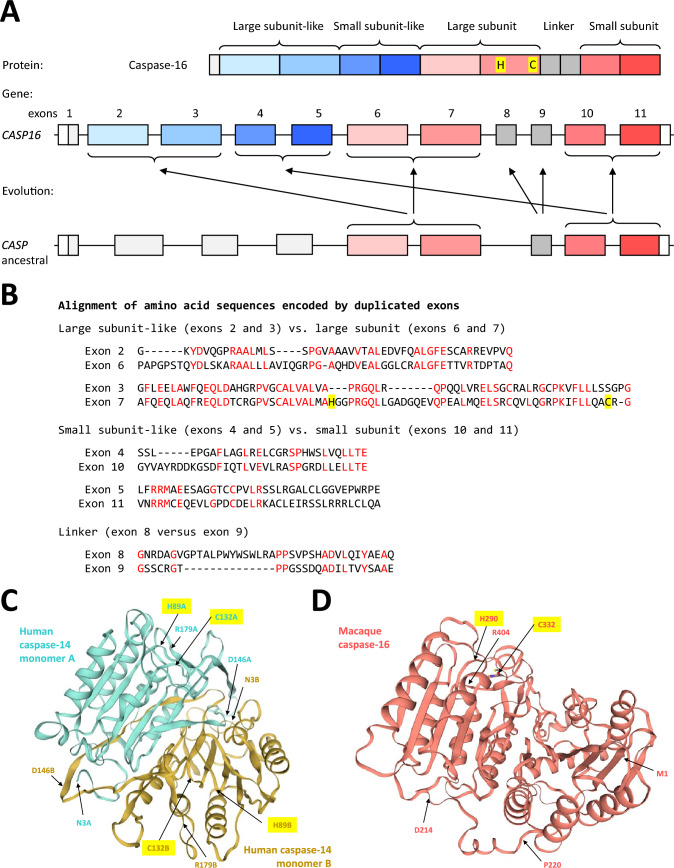
Exon duplications have led to the duplication of the caspase domain in caspase-16. **A** Schematic depiction of domains of the caspase-16 protein and exons of the corresponding gene. The model for evolution of exons is indicated by arrows from a putative ancestral *CASP* gene (assuming the same exon–intron structure as in *CASP1*) to *CASP16*. **B** Alignment of amino acid sequences encoded by different exons of chimpanzee *CASP16*. Red fonts highlight identical residues. Dashes were introduced to optimize the alignments. **C**, **D** Comparison of structure models of a human caspase-14 dimer and macaque caspase-16. Ribbon diagrams of a homo-dimer of human (*Homo sapiens*) caspase-14 (P31944) (https://swissmodel.expasy.org/repository/uniprot/P31944, last accessed on 29 January 2025) and Rhesus macaque (*Macaca mulatta*) caspase 16 (A0A5F8A9B6) (https://swissmodel.expasy.org/repository/uniprot/A0A5F8A9B6?model=AF-A0A5F8A9B6-F1-model-v4, last accessed on 29 January 2025). Note that an α-helix β-sheet α-helix sandwich fold characteristic for the caspase domain (pfam00656) is predicted for the monomers (turquoise ribbon, monomer A, and gold ribbon, monomer B) of caspase-14 (**C**) and for both the catalytic domain (upper left) and the prodomain (lower right) of caspase-16 (red ribbon) (**D**). A subset of residues are labelled. Yellow shading highlights cysteine and histidine residues of the catalytic dyad. The models are reproduced from the SWISS-MODEL repository (Waterhouse et al. 2018) under the CC BY-SA 4.0 Creative Commons Attribution-ShareAlike 4.0 International License (https://creativecommons.org/licenses/by-sa/4.0/) (Color figure online)

When we performed BLAST searches, we noticed that parts of the amino-terminal segment of chimpanzee caspase-16 showed sequence similarity to the caspase domain (pfam00656: peptidase_C14) (Mistry et al. 2021) present in the carboxy-terminal segment of the same protein. This sequence similarity suggested that duplications of gene sequences have occurred during the evolution of *CASP16*, as depicted schematically in Fig. 3A. Amino acid sequence alignments showed that the protein segments encoded by exons 2 and 3 are homologous to the large subunit, which is encoded by exons 6 and 7, of the caspase domain (Fig. 3B). Likewise, the protein segments encoded by exons 4 and 5 are homologous to the small subunit, which is encoded by exons 10 and 11, of the caspase domain (Fig. 3B). This pattern indicates that the prodomain of caspase-16 has evolved by duplication of the catalytic domain. The histidine and cysteine residues forming the catalytic dyad (Histidine 290 and Cysteine 332 of chimpanzee caspase-16) are not conserved in the prodomain (Fig. 3B). Structure models generated by the SWISS-MODEL homology-modelling pipeline (Waterhouse et al. 2018) suggest that the three-dimensional structure of the caspase-16 prodomain is similar to the caspase fold, and the overall structure of caspase-16 is similar to the structure of a caspase-14 homodimer (Fig. 3C, D).

### A Single-Nucleotide Deletion Leads to a Frameshift in Exon 3 of Human *CASP16P*

**Fig. 4:**
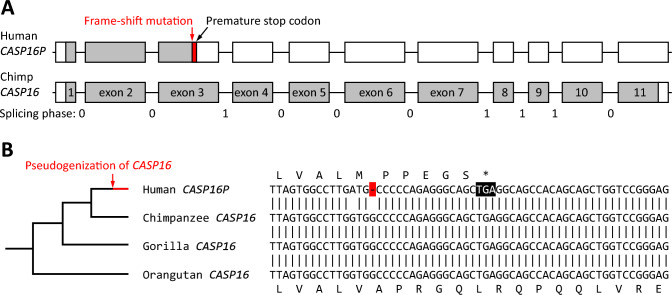
*CASP16* has undergone pseudogenization in humans after their evolutionary divergence from other primates. **A** Schematic depiction of human *CASP16P* and chimpanzee (chimp) *CASP16*. Exons are shown as boxes. Protein-coding segments are shaded grey and untranslated regions are white. The position of the frame-shift mutation in human *CASP16P* and the splicing phases between exons are indicated. **B** Alignment of nucleotide sequences homologous to the site of the frame-shift mutation in human *CASP16P*. A phylogenetic tree of the species is shown on the left. GenBank accession numbers: human (*Homo sapiens*) NC_000016.10, nucleotides 3,144,522–3,144,580; chimpanzee (*Pan troglodytes*) NC_072416.2, nucleotides 5,813,557–5,813,616; gorilla (*Gorilla gorilla gorilla*) NC_073242.2, nucleotides 6,791,343–6,791,402; orangutan (*Pongo abelii*) NC_072003.2, nucleotides 3,319,717–3,319,776

Finally, we compared the nucleotide sequences of the human ortholog of *CASP16* and *CASP16* genes of closely related primates. As expected, the orthologous genes of human and chimpanzee showed a high degree of conservation with more than 98% nucleotide sequence identity (Suppl. Fig. S7) and an identical exon–intron organization (Fig. 4A). However, a frameshift mutation is present in exon 3 of the human gene, which is therefore termed *CASP16P* to indicate that it is a pseudogene (Fig. 4A). The single-nucleotide deletion shifts the reading frame and leads to the termination of the coding sequence at the 6 th triplet downstream of the deletion (Fig. 4B).

The current annotation of *CASP16P* in GenBank predicts a non-coding RNA (accession number NR_132322.1, https://www.ncbi.nlm.nih.gov/nuccore/887247218, last accessed on 30 January, 2025) which begins at the aforementioned stop codon and lacks exons 1 and 2. A *CASP16P* transcript including exons 1 and 2 is predicted in the ENSEMBL database (accession number ENST00000635032.2). Human *CASP16P* is transcribed in the duodenum, small intestine and spleen (Suppl. Fig. S8A). Notably, the spleen is also a major site of *CASP16* expression in the cattle (Suppl. Fig. S8B). Analysis of the exon coverage and intron-spanning reads of aggregate RNA-seq data in the “Gene” view of *CASP16P* in National Center for Biotechnology Information (NCBI) GenBank indicated that the main *CASP16P* transcripts contain all 11 exons including exon 3 (Suppl. Fig. S9 A). These transcripts encode a carboxy-terminally truncated protein of 105 amino acid residues without a catalytic site (Suppl. Fig. S9B). The only potential start codon in frame with the catalytic domain is located in exon 7, and the following open reading frame corresponds to an amino-terminally truncated protein (Suppl. Fig. S9C) homologous to residues 288–470 of chimpanzee caspase-16. The resulting protein would lack not only the prodomain but also a portion of the catalytic domain, thereby precluding the formation of an active protease.

The analysis of RNA-seq reads in the “Gene” view of *CASP16P* in NCBI GenBank also indicates the existence of *CASP16P* mRNAs which lack exon 3 (Suppl. Fig. S9 A). The skipping of exon 3 removes the frameshift associated with the 1-nucleotide deletion within this exon. However, this mode of alternative splicing does not restore the reading frame because exon 2 ends in the splicing phase 0 (the last nucleotide of the exon corresponds to position 3 of a codon) and exon 4 begins in splicing phase 1 (the first nucleotide of the exon corresponds to position 2 of a codon). Accordingly, the reading frame is shifted at the junction of exons 2 and 4 and the coding sequence terminates prematurely (Fig. 4A; Suppl. Fig. S9D). Taken together, the currently available RNA-seq data suggest that transcription of *CASP16P* does not lead to the production a catalytically active caspase-16 in human tissues.

## Discussion

Caspase-16 has been known as a mammalian caspase for more than ten years (Eckhart et al. 2008; Sakamaki and Satou 2009; Svandova et al. 2024), but the structure of the *CASP16* gene and the conservation or loss of the caspase-16 protein in different species have remained unknown. The present study reveals that the unique primary structure of caspase-16 orthologs is conserved in many mammalian species (Suppl. Fig. S2), whereas a mutation has led to the pseudogenization of human *CASP16P*. The results of this study suggest the existence of inter-species differences depending on the presence or absence of caspase-16. Being a member of the caspase family of proteases, caspase-16 is predicted to control either programmed cell death or inflammation (Salvesen and Ashkenazi 2011; Van Opdenbosch and Lamkanfi 2019; Sakamaki and Satou 2009), but its function is not known at present.

The complete coding sequence of *CASP16* could be identified in the genomes of a broad variety of mammals (Fig. 1; Suppl. Fig. S2). The identification of a *CASP16* gene in the echidna, a phylogenetically basal mammal (Steinbinder et al. 2024), suggests that *CASP16* is at least 180 million years old, which corresponds to the divergence time of protherian (monotremes) and therian (marsupials and placentals) mammals (Zhou et al. 2021). However, caspase-16 is not conserved in all descendants of the last common ancestor of mammals, because *CASP16* has undergone pseudogenization in humans (Fig. 4), mouse (Suppl. Fig. S4) (Sakamaki and Satou 2009), platypus (Suppl. Fig. S3) and several other species (Eckhart et al. 2008; Strasser et al. 2015). In species that have retained an intact *CASP16* gene, amino acid residues critical for catalytic activity are conserved (Fig. 1; Suppl. Fig. S2), indicating that the function of caspase-16 depends on its enzymatic activity as a protease.

The pseudogenization of *CASP16* in humans and the main mammalian model species of molecular biology, the mouse, poses challenges to the functional characterization of caspase-16. The tissue expression pattern of *CASP16* is only incompletely known from transcriptome studies and reverse transcription-PCR analyses, indicating the expression of *CASP16* in the bovine spleen (Suppl. Fig. S8B) and the liver of the opossum (Eckhart et al. 2008), respectively. The detection of transcripts of *CASP16P* in human spleen (Centola et al. 1998; Eckhart et al. 2008) is not informative about the role of caspase-16 because these transcripts do not encode a protein. The functions of caspase-16 should be studied either in biomedical models, such as the rat and the rabbit, which have an intact *CASP16* gene (Suppl. Fig. S2 A), or in species, such as cattle, pig or dog (Suppl. Fig. S2 A), that are subjects of research in veterinary medicine.

Our identification of internal sequence similarities between the amino-terminal segment and the caspase domain of caspase-16 (Fig. 3) suggests that the prodomain of caspase-16 is derived from an ancestral caspase domain. According to the model proposed in Fig. 3B, the duplication of the exons encoding the large and small subunit of the caspase domain has led to the duplication of the caspase domain. Notably, the two exons encoding the inter-subunit linker segment of the carboxy-terminal domain have originated by another exon duplication. This model of exon duplications is compatible with the differences in exon–intron structures of *CASP16* and *CASP1* (Fig. 3A) and with the comparison of *CASP16*, *CASP15* and *CASP14* (Suppl. Fig. S10). *CASP14*, the phylogenetically closest relative of *CASP16*, contains exons homologous to the caspase domain-encoding exons of *CASP15* and *CASP1* including only one exon for the inter-subunit linker (Suppl. Fig. S10). The conservation of sequence features (Fig. 3B) and splicing phases (Suppl. Fig. S10) indicates the origin of specific exons in *CASP16*. Domain duplications have occurred during the evolution of a significant fraction of all proteins (Chothia et al. 2003; Björklund et al. 2006; Schüler and Bornberg-Bauer 2016). Duplications of DEDs and CARDs through partial gene duplications or gene fusions have occurred multiple times during the evolution of caspases and caspase-interacting proteins (Grenet et al. 1999; Eckhart et al. 2008; Eckhart et al. 2009; Korithoski et al. 2015), but – to best of our knowledge – the duplication of the caspase domain has not been reported yet for caspases of vertebrates.

The presence of two caspase domains (pfam00656) in a single protein is intriguing because the catalytic activity of caspases depends on the dimerization of caspase domains, which is canonically achieved by the dimerization of two caspase molecules (Boatright and Salvesen 2003). In caspase-16, the tandem arrangement of the amino-terminal caspase-like domain and the carboxy-terminal caspase domain is likely to facilitate direct interactions between these domains. Indeed, the homology-based structure model of caspase-16 of the rhesus macaque suggests that the prodomain assumes a stable fold and interacts with the caspase domain in the carboxy-terminal region of the protein (Fig. 3C, D). As the amino acid residues required for catalytic activity are conserved only in the carboxy-terminal domain of caspase-16 (Fig. 3), a single catalytic site is present in the putative caspase domain dimer. This situation is comparable to the heterodimerization of caspase-8 and a catalytically inactive paralog of caspase-8, named FLIP(L) (which stands for Fas-associated death domain protein-like interleukin-1β-converting enzyme-like inhibitory protein, long form). Caspase-8 is activated within this heterodimer (van Raam and Salvesen 2012), suggesting that also caspase-16 may assume a confirmation compatible with enzymatic activity. In an alternative scenario, the amino-terminal and/or the carboxy-terminal domains of two caspase-16 proteins might undergo intermolecular homodimerization. These hypotheses requires experimental testing in future studies.

By determining the exon–intron organization of *CASP16* in mammals and localizing a 1-nucleotide deletion in the human *CASP16P* gene, our study reveals the molecular basis for the evolutionary loss of caspase-16 in humans. The frameshift mutation of *CASP16P* is absent from closely related species of hominids. This suggests that the mutation has occurred after the divergence of the lineages leading to humans and chimpanzees approximately 6 million years ago (Kumar et al. 2022). In this regard, the pseudogenization of *CASP16* is comparable to the pseudogenization of *CASP12*, which also occurred in humans but not in chimpanzees (Fischer et al. 2002; Puente et al. 2005). Loss-of-function mutations play important roles in evolution especially with regard to the evolution of innate immunity (Zhang et al. 2010; Rausell et al. 2020; Lopes-Marques et al. 2024). The results of the present study provide the basis for the further characterization of caspase-16 and its role in the evolution of mammalian species.

## Methods

### Identification and Analysis of Nucleotide and Amino Acid Sequences

Nucleotide sequences of CASP genes were either obtained from annotated genome sequence assemblies in GenBank (National Center for Biotechnology Information) or identified by Basic Local Alignment Search Tool (BLAST) searches in genome sequences without annotation of genes of interest. Information about gene transcription levels in different organs was obtained from the “Expression” section of the NCBI GenBank “Gene” view (https://www.ncbi.nlm.nih.gov/gene/, last accessed on December 22, 2024). The *CASP16* gene locus was analyzed in the genome sequences of the following species: chimpanzee (*Pan troglodytes*), genome assembly NHGRI_mPanTro3-v2.0_pri (GenBank acc. nr. GCF_028858775.2, submitted by National Human Genome Research Institute); cattle (*Bos taurus*), genome assembly ARS-UCD2.0 (GenBank acc. nr. GCF_002263795.3, submitted by USDA ARS); opossum (*Monodelphis domestica*), genome assembly mMonDom1.pri (GenBank acc. nr. GCF_027887165.1, submitted by Vertebrates Genomes Project); echidna (*Tachyglossus aculeatus*, GenBank accession number NC_052096.1, submitted by the Vertebrates Genomes Project), platypus (*Ornithorhynchus anatinus*), genome assembly mOrnAna1.pri.v4 (GenBank acc. nr. GCF_004115215.2, submitted by Vertebrates Genomes Project), and human (*Homo sapiens*), genome assembly GRCh38.p14 (GenBank acc. nr. GCF_000001405.40). The orthology of genes was inferred by the detection of shared local synteny, best reciprocal sequence similarity (Kristensen et al. 2011) and phylogenetic analysis, as described below. Nucleotide sequences were aligned with BLASTn and MultAlin (Corpet 1988). Amino acid sequences were aligned with MUSCLE (Edgar 2004) and MultAlin (Corpet 1988). Three-dimensional protein structure models were downloaded from the SWISS-MODEL repository, which utilizes the SWISS-MODEL homology-modelling pipeline (Waterhouse et al. 2018).

### Analysis of mRNA Splicing Using RNA-seq Data Available in NCBI GenBank

The structure of human *CASP16P* mRNAs was investigated by analyzing RNA-seq exon coverage and RNA-seq intron-spanning reads in the “Gene” view of *CASP16P* on NCBI GenBank (https://www.ncbi.nlm.nih.gov/gene/197350, last accessed on March 27, 2025). We analyzed the histograms from aggregate RNA-seq data (NCBI Homo sapiens Annotation Release 110) that show exons and the splicing of exons (“RNA-seq intron-spanning reads”) in the “Genomic Regions, Transcripts, and Products” section of the “Gene” view. Further details are provided in Suppl. Fig. S9 A and the corresponding legend.

### Molecular Phylogenetics

Multiple sequence alignments and phylogenetic analyses were performed as described previously with modifications (Steinbinder et al. 2024). In brief, amino acid sequence alignments were generated with MUSCLE (Edgar 2004) in Aliview (Larsson 2014). The alignment of amino acid residues corresponding to the caspase domain was used for the phylogenetic analysis. The best fitting amino acid substitution model was JTT (Jones et al. 1998), as determined with Prottest (Version 3.0) (Darriba et al. 2011). The Akaike information criterion (Akaike et al. 1998) was used to estimate the likelihood of the best model. IQ-Tree2 (version: 2.6.3) (Minh et al. 2020) was used to calculate maximum likelihood-based phylogenetic trees with 1000 bootstrap replicates to assess statistical support of the generated tree. A bayesian inference tree was calculated with the same alignment and amino acid substitution model according to a published approach (Ehrlich et al. 2020). We performed two parallel Markov chain Monte Carlo (MCMC) runs of four chains each with 5 million generations and a sampling frequency of 1 per 1000 generations. The phylogenetic trees were visualized with FigTree, version v1.4.4 (http://tree.bio.ed.ac.uk/software/figtree/, last accessed on January 29, 2025). The raw images were edited with Inkscape (version: 1.3.0; https://inkscape.org/de/, accessed on January 29, 2025).

## Supplementary Information

Below is the link to the electronic supplementary material.

Supplementary file1 (DOCX 1404 KB)

## Data Availability

All data generated or analysed during this study are included in this published article and its supplementary information files.
