# Supplementary material for: Caspase Domain Duplication During the Evolution of Caspase-16
Source: J Mol Evol. 2025 May 20;93(3):395–405. doi: 10.1007/s00239-025-10252-w (PMC12198278; doi:10.1007/s00239-025-10252-w)
Supplement: Supplementary file 1 — Supplementary file1 (DOCX 1404 KB) [file 239_2025_10252_MOESM1_ESM.docx]

**Supplementary Information**

**Caspase domain duplication during the evolution of caspase-16**

Leopold Eckhart^1^*, Attila Placido Sachslehner^1^, Julia Steinbinder^1^, Heinz Fischer^2^

1. Department of Dermatology, Medical University of Vienna, Vienna, Austria

2. Division of Cell and Developmental Biology, Center for Anatomy and Cell Biology, Medical University of Vienna, Vienna, Austria

*Corresponding author. E-mail: leopold.eckhart@meduniwien.ac.at

**Content**

Supplementary Figures S1-S10

TCAGGTAGGGGCATGTCAGTGCCAGCAAAGAAGTCACCTAAGCCAGTTACAGGAGCCCAGGCTGGGAGAGTGGGGCCAGGACCCAGGGTT

CCAGGGAGGGGGCAGAGTCCAGCTCAGCTCATAAGCAGAGCTGATAGCACTCGTTAGGGTAGAGGGCAAGGGTTAGGACCCAGGGCACTG

AGAGCCCAGGGCCAGGAGGTGAGCTCCGAGCTTGGTGCTTGGAGGAAGCCCCGTCTGGCCCTATGGCCTTCCTGGTGGCTGGGACCCTGC

                                                               M  A  F  L  V  A  G  T  L

GGCTGGCCATGCAGATGGCAGACACTCGAGACAGCCTGGGGAGAAAG**GT**AGCAGGAGGCACCGCCTAGCCCAGCTGCTCCTGCTGAGCCC

R  L  A  M  Q  M  A  D  T  R  D  S  L  G  R  K

CAGCCCAGCCCAGCCCAGCTCTGGCCTTGCTTCCCC**AG**GGGAAGTATGACGTCCAGGGTCCAAGGGCAGCCCTGATGCTCAGCAGCCCTG

                                       G  K  Y  D  V  Q  G  P  R  A  A  L  M  L  S  S  P

GGGTGGCGGCCGCTGTAGTCACTGCCCTGGAGGACGTGTTCCAGGCCCTGGGCTTTGAGAGCTGCGCGAGGAGGGAGGTCCCGGTCCAG**G**

G  V  A  A  A  V  V  T  A  L  E  D  V  F  Q  A  L  G  F  E  S  C  A  R  R  E  V  P  V  Q

**T**GAGCCTCTGCCTCTTACATCCACCCTCAGGCCCAGCACCGACCCCTTCCCCACTCTCCCCTGACCCAGAATCTCCTCCCCACCCAAAAC

CTCCCCGGACTCAGGTCGGTCTCACTCTTGCCCCT**AG**GGCTTCCTCGAGGAACTGGCTTGGTTCCAGGAGCAGCTGGATGCCCACGGGCG

                                      G  F  L  E  E  L  A  W  F  Q  E  Q  L  D  A  H  G  R

CCCTGTGGGGTGTGCCTTAGTGGCCTTGGTGGCCCCCAGAGGGCAGCTGAGGCAGCCACAGCAGCTGGTCCGGGAGCTGAGCGGCTGCCG

  P  V  G  C  A  L  V  A  L  V  A  P  R  G  Q  L  R  Q  P  Q  Q  L  V  R  E  L  S  G  C  R

GGCCCTGCGGGGCTGCCCCAAAGTCTTCCTGCTGCTCTCAAGTGGCCCTGGGT**GT**GAGTGAGCTGGGTCAGGATCCAGGAGCTGGGCAGG

  A  L  R  G  C  P  K  V  F  L  L  L  S  S  G  P  G

GACCCAGGGGCAGAGCCTCGGGCCTCACTGCAGGCCAACATGCTGCTTCTCCACCC**AG**CCTCCCTGGAGCCCGGAGCCTTCCTTGCTGGC

                                                          S  S  L  E  P  G  A  F  L  A  G

CTGAGAGAGCTGTGTGGCCGCTCTCCTCACTGGTCCCTGGTGCAGCTGCTGACGGAG**GT**GGGGACGCTGGAGGGGGAGGCCCAGGGAAGC

 L  R  E  L  C  G  R  S  P  H  W  S  L  V  Q  L  L  T  E

GGGGCTGGTCCTGCTGTCTCTGCTGGTTCTGCTGTGCCCCCCTAAGCCAGTTACAGTTAGATTCATTCACTTGTATCCTCCCTCCGATTC

ATTCTACAAACCTCCTTTATTGTCTACAGAAGTGGTCTCAGCCCCTCACCCTCAACTCGGGACCCCTGACAACCCACTCAGGATCCCCGA

CACTCGACTCCTCCCAACTCCATGCCTTGGCCCAAGCGGTTCCCTCTGCCTGCTATGCCTTCCTGTTGTCCCGGGAGCCCTGCACAGTCC

TCTGCAGGCTGCCAGCGATGCCCTTCCTTGCATCCACGCTTGCACTTCAGGCTGCCCCTTTCTACACCAAGCAGTGGCTGTCCGCCCTGT

TAGATCGCAGGACCCTGGAGGCTGGGACTGGGAATGCTTCAAGTCTGGTCTCCAAGGCTGAAGGTCTCACTGTGTAGTTGGGTGGCCCCT

GGGCAGCCCTGGGGATGAGGGGGCCCCAGCTCTCCTCCACAGATCGCCCAGCAGGGTAGTGCCCACCCCGTGAGTCTTCCTGACAGGCCC

AGCCCCGCTCTAGGGAACATCCCCACTTCTCTGCGCACTGATCCCGGGGCAGGGGTTAATGGTGTGGCCACTGTCCACTCGAGGCCATCC

ATGCCCAGGGAAGTCCATAACCTTGAGCCTGAACTGGAGCCTGGGCCTTGCTTGAGGCCATCCTGCCTGTCTGTCCTTTGCTGGCCTCCT

CCTGTCTGCATTCTGGCAGGTGACCCTGAAGAGACTACAGAAGCCACAAGCCTGGCTGAGTCTTTTCTCGGCCCCATCCCC**AG**CTCTTCC

                                                                                    L  F

GCAGGATGGCTGAAGAGTCCGCAGGGGGCACCTGCTGCCCCGTCCTTAGGAGCTCCTTGAGGGGGGCACTGTGCCTGGGAGGCGTGGAGC

R  R  M  A  E  E  S  A  G  G  T  C  C  P  V  L  R  S  S  L  R  G  A  L  C  L  G  G  V  E

CCTGGAGGCCTGAG**GT**GAGGGGGGCAGGGCAGGGGTCCAATCACATGGCCACAGTTTCCAGTGGGACAGAAGCTTAGGGGGACCCAGGCC

P  W  R  P  E

GGGGAGGCCAGGAGCTGGAGTCCTCTCAGGCCACTTTAGATGTGTCTTAACCCTCTCTGC**AG**CCGGCCCCCGGTCCCAGCACACAGTATG

                                                               P  A  P  G  P  S  T  Q  Y

ACCTGTCCAAGGCCAGGGCTGCCCTCCTCCTGGCTGTGATCCAAGGCCGGCCTGGGGCCCAGCATGACGTGGAGGCGCTGGGGGGCCTGT

D  L  S  K  A  R  A  A  L  L  L  A  V  I  Q  G  R  P  G  A  Q  H  D  V  E  A  L  G  G  L

GCCGGGCCCTGGGCTTTGAGACCACCGTGAGAACGGACCCTACAGCTCAG**GT**GAGGGGAAGCCGAGAACTTCCGCTGGTGCTCTGAAGGA

C  R  A  L  G  F  E  T  T  V  R  T  D  P  T  A  Q

AGACCACCCCTCCCTAGAAACCTGGGGCCTCTCTCCTGGGCCTCTCTCCATCACCGGCAGGAAGTGCACCACAAGTCTAGCCTCTGTGCT

CCCTGTTGCCTGCATCTGGTCCGTCACTTCCCTGCCTCTGGAAGCCTGGTCTCCAGGGTCCCCGAGGCCTCCCTCACTGGCTGGTTCTCT

GGCCCCCCGCCCCCTCCCCAGCTCAAAGCTTTAGCTCCAAGTCTTGGTTTCCCTCTTGGCTCCCAGCAGCCCACTCCGCTCTCCTCACAC

CTCTCAACTTCTTGGTGCGGCTTCCCCACGAGGGCAGGAGGAGAACTGGCTCCAGGAAGCTGGGTCTCTACGTCACCTCTAAAGAGGCCA

TGCCAAGGCCTTGCAGGAGGGAGTTAGAAAAGGGCTTCTGGCCGGGCGCAGTGGCTCACGCCTGTAATCCCAAAACTTTAGGAGGCTGAG

ACGGGTGGATCACTTGAGATTAGGAGTTTGAGACCAGCCTGACTACCACGGTGAAACCTCGTCTCTACTATAAAGACAAAATTAGTAGGG

CATGGTGGTGCGTGCCTGTAATCCCAGCTACTTGGGAGGCTGAGGCAGGAGAATGGCTTGAACCCAGGAGGCCGAGGTTGCAGTGAGCTG

AGATCGGGCCACTGCACTCCAGCCTGGGCGACAGAGCAAGACTCCGTCTCAAAAAACAAAAACAGGCCGGGCACAGTGGCTCACGCCTGT

AATCCCAGCACTTTGGGAGGCCGAGGCAAGCGGATCACGACGTCAGGAGATCGAGACCATTCTGGCTAACACAGCGAAACCTCATCTCTA

CTAAAAATACAAAAAATTAGCCGGGTGTGGTGGCATATGCCTGTATTCCCAGCTACTCAGGAGGCTGAGGCAGGAGAATCGCGTGAACCC

AGGAGGCAGAGATAGCAGTGAGCCGAGATCGCGCCACTGCACTCCAGCCTGGGTGACAGAGCAAGACTCCTTCTCAAAAAAAAAAAAAAA

AAAAAAAAGAGGGCTTCCTCCCTGGACCTGTGAGTGGCAGGCGGTGGGAGGCCAGGTGGGGCAGGGTCTGGGAAACCTTGTCAGCCTCAC

AGAGGGCAGCCAGTGGCTGGGGAGGTGGTGGCTTTGGCCCAGGCCTCAACATTGTTCCCACCCC**AG**GCTTTCCAGGAGCAGCTGGCCCAG

                                                                   A  F  Q  E  Q  L  A  Q

TTCCGGGAGCAACTGGACACCTGCAGGGGCCCTGTGAGCTGTGCCCTTGTGGCCCTGATGGCCCATGGGGGACCACGGGGTCAGCTGCTG

 F  R  E  Q  L  D  T  C  R  G  P  V  S  C  A  L  V  A  L  M  A  H  G  G  P  R  G  Q  L  L

GGGGCTGACGGGCAAGAGGTGCAGCCCGAGGCACTCATGCAGGAGCTGAGCCGCTGCCAGGTGCTGCAGGGCCGCCCCAAGATCTTCCTG

 G  A  D  G  Q  E  V  Q  P  E  A  L  M  Q  E  L  S  R  C  Q  V  L  Q  G  R  P  K  I  F  L

TTGCAGGCCTGCCGTGGGG**GT**GAGCGGCCCGGCCTCCTACTGCCCTCACTTTCCTCGGCCAAGCTTCAGCCCCCGGGACTCACGGTCTAC

 L  Q  A  C  R  G

CTTCTCCAGGGAGCCCGGGTACCCGCCCTTCCCTGCCCCCTCTCCTGTCCTATCCTAGAGGTCAAGTCCACGACCTTGAACCCTTAACTC

TCAACACCTGTCATTCAGCGCTCTGAATGTCTCCAGTCTGGCAAGCCTGCCCTGGAGCTCTGGAGTTGGGTTCTCACCTTGACCCCACAT

TCACACTAGACCCCTGAGCACCCCCAGGTATCCCCGGAGTGAGACTATCTGCCTCTCCCCACCCTCTTC**AG**GAAACAGGGATGCTGGTGT

                                                                       G  N  R  D  A  G  V

GGGGCCCACAGCTCTCCCCTGGTACTGGAGCTGGCTGCGGGCACCTCCATCTGTCCCCTCCCATGCAGACGTCCTGCAGATCTACGCTGA

  G  P  T  A  L  P  W  Y  W  S  W  L  R  A  P  P  S  V  P  S  H  A  D  V  L  Q  I  Y  A  E

GGCCCAAG**GT**GGGTTCTGCCTTCCTTCCAGGGCCTGGGCTTGGGCAGGGCTGGTTGTGGGGACTGTCCAGAGAGCATCTCCAGGGCTCTA

  A  Q

AGCTGGGGTATGGCTGCCACCTGCATCCTCTGTTTGCCAAGACAATGGGAAGAAAAAAATCTTCCTAAACCGCAAGGGCCTTTGGGAAGT

GGGAGCTTCTTCCCCTGTTGGAGCCTGGCAAGAACGCTGGAGTCGGCAAGGTCAAATAGCTTTTCTGAGGTCACAGCTGGTAAGTGGCTG

GGCCGAGCTTTGAACTTCCGTCTGTCATTCCTGCCCTGCACTCTTTCCACCTCCCTGGGCTGCCCTTAAGCCACAGATGGGGAGCTCCCG

GGGCTGATGGAGTTCCACGATGTTGATCACTGGAATTGATTCCTCTTGC**AG**GCAGCTCCTGCAGGGGCACCCCTCCAGGGAGCTCTGACC

                                                   G  S  S  C  R  G  T  P  P  G  S  S  D

AAGCAGACATACTGACGGTCTACTCAGCCGCAGAGG**GT**AAGGAGATGGGTCATCGGGAGCCTGTGGTTACACAGGGCCCAGCTTCCTGGC

Q  A  D  I  L  T  V  Y  S  A  A  E

CTAAGATCTGGAGTAGCCTTAGGGGCAGCTAGGGCTTAGGGTTGGGGCACAGAGATGCCAGCCCAGCTGTGGTCCAGCCATGTTCCCTAC

ATGGGTTATGATGTGTAGTAACAGCAGTGATGGTGAGTGCTGTGAGGCGCAGCTGTGCCAACCACTGTGTGTGCAGGTTTCCTCTAGGCT

GTGAGTTCCACGAGGCCAGGGTAGATCTGCCTGCCCCAAGGCCCTGCTCAGTGTCTGGCACATAGTAGGTGCACAGTAAATGTTTGTTCA

GTAGTGAATCTCTCCATAGGCTCACCTCTGCAAATACCTAGCAACAACTTGTTCCAGATGCAAGAAGTCCCTGCTCCCTGCCCTGTTCTC

TTGCCTGATTCCTGGGTCCTGCCTCCTTGTACCCCACCTTCCACCAACAATAGGACCCCTGGGATTGGAAGGCAGAGGGTTGGGGCCTTG

GTCTGATGCTCTGGCCCTTATCCCCTGACCT**AG**GCTATGTGGCCTATCGCGATGACAAGGGCTCAGACTTTATCCAGACACTGGTGGAGG

                                 G  Y  V  A  Y  R  D  D  K  G  S  D  F  I  Q  T  L  V  E

TCCTCAGAGCCAGCCCCGGGAGAGACCTTCTGGAGCTGCTGACTGAG**GT**GTGTTGGGGGGTTCCAGGGTGACAAGTGGCAAGGAGCTGGG

V  L  R  A  S  P  G  R  D  L  L  E  L  L  T  E

TTTGCCCTTCTCCCCAGCCCTGGTATTCTGATCACCTCCTATGAACTCCATTGGCAAAGGAGGGATCCTCTGCCCTCAATACTACAAGAT

AACCAAACGCAGGATGGCCGACGCTGCACAGATGCCATCCACTGCAGTTCTTAGTCACACGTACTGCAGTCGGGTGGGGAGGAGGACACT

GCATGCCATGCGGGGCCACCTGGGCTTGTGCTCAGAGCAGGGTGAACCTGCAGGGCCAGTGGGAAGCTGGCTTTGTAGTGACAAGAGTGT

GAGATGCCCCCTGGTTCCCACGGGCAGATGTGGTTGGTTGGTTTGAATATTTCCAAGGCCTGTCAGGGGGCTGAAGTCCATTAGGGTGAC

GACCAGGTGGGGTGCAGCTGGTCTGCTGAGAGGGGACCTACGGGGTGGGAGCCTGTCCTGCTGGGTGGGGACATGTCTGGCCAGAGCAGA

GGAATTCACCGTTAGGCCTCTGGAGCTCTGCGAGCCTCAAAGATGTCCAGGCAGTCCTTGAAATTTTAGGCCTTGCAATGTACCAAGCCA

GGGGCTCTCCCCTGGGGCAGGCTGAGCCCCGGAGGGCTGTAGCCCCGGGCACAGGCTGGGTGTGGTTCTC**AG**GTCAACAGGCGGATGTGT

                                                                         V  N  R  R  M  C

GAGCAGGAGGTGCTGGGCCCCGACTGCGATGAACTCCGCAAGGCCTGCCTGGAGATCCGCAGCTCGCTCCGGCGCCGGCTCTGCCTCCAG

 E  Q  E  V  L  G  P  D  C  D  E  L  R  K  A  C  L  E  I  R  S  S  L  R  R  R  L  C  L  Q

GCCTGAGGGTGCGGCGGCCACGGGGGCGCTGCTGAGACGGTGGCCAGATCCCAGCGCCATTCTTGCCTCCATCCACCCCCCATCCCCCCG

 A  *

**Supplementary Figure S1. Chimpanzee *CASP16*.** The coding sequence of *CASP16* of the chimpanzee (*Pan troglodytes*) is highlighted by red fonts. The amino acid sequence translated from this coding sequence is shown below the nucleotide sequence. Splice donor and acceptor sites at the ends of introns are underlined. The nucleotide sequence corresponds to GenBank accession number NC_072416.2, nucleotides 5812821-5818940.

**A**

**Examples of caspase-16 proteins of mammalian species**

| **Species (common name)** | **Species (scientific name)** | **GenBank accession number** | **Protein name (GenBank, January 1, 2025)** | **Protein name, this study** |
| --- | --- | --- | --- | --- |
| Chimpanzee | *Pan troglodytes* | XP_523278.4 | uncharacterized protein CASP16 | caspase-16 |
| Gorilla | *Gorilla gorilla gorilla* | XP_004057200.3 | caspase-14-like | caspase-16 |
| Olive baboon | *Papio anubis* | XP_021787375.1 | uncharacterized protein LOC101020562 | caspase-16 |
| Rhesus macaque | *Macaca mulatta* | XP_014980933.2 | caspase-14-like isoform X2 | caspase-16 |
| Crab-eating macaque | *Macaca fascicularis* | XP_005591104.3 | caspase-14 | caspase-16 |
| Nancy Ma's night monkey | *Aotus nancymaae* | XP_064218437.1 | caspase-14-like isoform X4 | caspase-16 |
| Common marmoset | *Callithrix jacchus* | XP_009007271.3 | uncharacterized protein LOC100403233 isoform X5 | caspase-16 |
| Gray mouse lemur | *Microcebus murinus* | XP_012599027.1 | uncharacterized protein LOC105859632 | caspase-16 |
| Sunda flying lemur | *Galeopterus variegatus* | XP_008564539.1 | PREDICTED: putative caspase-16 | caspase-16 |
| Rabbit | *Oryctolagus cuniculus* | XP_008248858.1 | caspase-14 isoform X1 | caspase-16 |
| Rat | *Rattus norvegicus* | XP_063126149.1 | uncharacterized protein Casp16 isoform X1 | caspase-16 |
| Indian flying fox | *Pteropus giganteus* | XP_039741050.1 | caspase-14-like isoform X1 | caspase-16 |
| Brandt's bat | *Myotis brandtii* | XP_005873055.2 | PREDICTED: caspase-14 | caspase-16 |
| Malayan pangolin | *Manis javanica* | XP_017512178.2 | caspase-14-like isoform X1 | caspase-16 |
| Dog | *Canis lupus familiaris* | XP_038396551.1 | caspase-14 | caspase-16 |
| Brown bear | *Ursus arctos* | XP_026341365.2 | uncharacterized protein LOC113245433 isoform X1 | caspase-16 |
| Cat | *Felis catus* | XP_003998970.4 | caspase-14 isoform X1 | caspase-16 |
| Cattle | *Bos taurus* | XP_005224700.2 | caspase-14 isoform X2 | caspase-16 |
| Pig | *Sus scrofa* | XP_013851189.1 | uncharacterized protein LOC100627836 | caspase-16 |
| Cape golden mole | *Chrysochloris asiatica* | XP_006874042.1 | PREDICTED: caspase-14 | caspase-16 |
| Lesser hedgehog tenrec | *Echinops telfairi* | XP_045141910.1 | uncharacterized protein LOC101647320 | caspase-16 |
| Gray short-tailed opossum | *Monodelphis domestica* | XP_016279603.1 | uncharacterized protein CASP16 isoform X1 | caspase-16 |
| Echidna | *Tachyglossus aculeatus* | XP_038609296.1 | putative caspase-16 | caspase-16 |

**B**

>XP_523278.4 **CASP16 [*Pan troglodytes*]**

MAFLVAGTLRLAMQMADTRDSLGRKGKYDVQGPRAALMLSSPGVAAAVVTALEDVFQALGFESCARREVPVQGFLEELAWFQEQLDAHGRPVGCALVALVAPRGQLRQPQQLVRELSGCRALRGCPKVFLLLSSGPGSSLEPGAFLAGLRELCGRSPHWSLVQLLTELFRRMAEESAGGTCCPVLRSSLRGALCLGGVEPWRPEPAPGPSTQYDLSKARAALLLAVIQGRPGAQHDVEALGGLCRALGFETTVRTDPTAQAFQEQLAQFREQLDTCRGPVSCALVALMAHGGPRGQLLGADGQEVQPEALMQELSRCQVLQGRPKIFLLQACRGGNRDAGVGPTALPWYWSWLRAPPSVPSHADVLQIYAEAQGSSCRGTPPGSSDQADILTVYSAAEGYVAYRDDKGSDFIQTLVEVLRASPGRDLLELLTEVNRRMCEQEVLGPDCDELRKACLEIRSSLRRRLCLQA

>XP_004057200.3 **CASP16-partial [*Gorilla gorilla gorilla*]**

MQMADTRDSLGRKGKYDVQGPRAALMLSSPGVAAAVVTALEDVFQALGFESCKRREVPVQGFLEELACFQEQLDAHGRPVGCALVALVAPRGQLRQPQQLVRELSGCRALRGCPKVFLLLSSGPGSSLEPGAFLAGLRELCGCSPHWSLVQLLTELFHRVAEESAGGTCCPVLRSSFRGALCLGGVEPWRPEPAPSPSTQYDLSKARAALLLAVIQGRPGAQHDVEALGGLCRALGFETTVRTDPTAQAFQEELAQFREQLDTCRGPVSCVLVALMAHGGPRGQLLGADGQEVQPEALMQELSCCQVLQGRPKIFLLQACRGGNRDAGVGPTALPWYWSWLRAPLSVPSHADVLQIYAEAQGSSCRGTPPGSSDQADILTVYSAAEGYVAYRDDKGSDFIQTLVEVLRANPGRDLLELLTEVNRRVCEQEVLGPDCDELRKACLEIRSSLRRRLCLQA

>XP_021787375.1 **CASP16 [*Papio anubis*]**

MAFLVAGTLRVATQMADTRDSLGRKGKYDVRGPKAALMLISPGVVAAAVTALEDVFQALGFESWERRQVPVQGFLEKLAWFRELLDAHRHPVGCALVALVAPKGQLKQPQQLVRELSGCGALRGCPKVFLLLSSGPGSALESGAFLAGLRELCGRSPHWCLVQLLTELFRKVTEESTGGTCCPVLRSSLRGALCLGGVEPWRPELAPGPNTQYDLSKTRAALLLAVIQGRPGAQHDVEALGGLCRALGFETTVRTDPTAQAFQEELAQFQKQLDTCRGPVSCVLVALMAHGGPRGQLLGADGQEVQPEALMQELSRCRVLWGRPKVFLLQACRGGNRDAGVGPTALPWYWSWLRAPPSVPSHADVLQIYAEAQGSSCRGAPPGRSDQADILTVYSAAEGYVAYRDDKGSDFIQTLVEVLRANPGRDLLELLTEVNRRVCEQDVLGPDCDELRKACLEIRSSLRRRLCLQP

>XP_014980933.2 **CASP16 [*Macaca mulatta*]**

MAFLVAGTLRVATQMADTRDSLGRKGKYDVRGPKAALMLISPGVVAAAVTALEDVFQALGFESWERRQVPVQGFLEKLAWFRERLDAHRHPVGCALVALVAPRGQLKQPQQLVRELSGCGALRGCPKVFLLLSSGPGSALESGAFLAGLRELCGRFPHWSLVQLLTELFRKVTEESTGGTCCPVLRSSLRGALCLGGVEPWRPELAPGPNTQYDLSKTRAALLLAVIQGRPGAQHDVEALGGLCRALGFETTVRTDPTAQAFQEELAQFQKQLDTCRGPVSCVLVALMAHGGPQGQLLGADRQEVQPEALMQELSRCRVLWGHPKVFLLQACRGGNRDAGVGPTALPWYWSWLRAPPSVPSHADVLQIYAEAQGSSCRGAPPGRSDQADILTVYSAAEGYVAYRDDKGSDFIQTLVEVLRANPGRDLLELLTEVNRRMCEQDVLGPDCDELRKACLEIRSSLRRRLCLQP

>XP_005591104.3 **CASP16 [*Macaca fascicularis*]**

MAFLVAGTLRVATQMADTRDSLGRKGKYDVRGPKAALMLISPGVVAAAVTALEDVFQALGFESWERRQVPVQGFLKKLAWFRERLDAHRHPVGCALVALVAPRGQLKQPQQLVRELSGCGALRGCPKVFLLLSSGPGSALESGAFLAGLRELCGRFPHWSLVQLLTELFRKVTEESTGGTCCPVLRSSLRGALCLGGVEPWRPELAPGPNTQYDLSKTRAALLLAVIQGRPGAQHDVEALGGLCRALGFETTVRTDPTAQAFQEELAQFQKQLDTCRGPVSCVLVALMAHGGPQGQLLGADRQEVQPEALMQELSRCRVLWGRPKVFLLQACRGGNRDAGVGPTALPWYWSWLRAPPSVPSHADVLQIYAEAQGSSCRGAPPGRSDQADILTVYSAVEGYVAYRDDKGSDFIQTLVEVLRANPGRDLLELLTEVNRRMCEQDVLGPDCDELRKACLEIRSSLRRRLCLQP

>XP_064218437.1 **CASP16 [*Aotus nancymaae*]**

MAFLVAGTLRVAMQMADTRDSLERKGKYNVQGPRAALMLSSPGVAAAAVTALKDVFQALGFESCERREVPVQGFLKELAWFRERLDAHGCLVGCALVALVAPRGQLWQPQQLVRELSSCGVLQGCPKVFLLLPSGPGATLEPGAFLDGLRELCGRSPHWSLVQLLTELFHRVAEESTGGTCCPVLQSSLRGALCLGGVEPWRPEPATGPSTQYDLSKTRAALLLAVIQGRPGAQHDVEALGGLCRALGFETTVRTDPTAQAFQEELAQFREQLDTCRGPVSCALVALMAHGGPRGQLLGSDGQEVHPQALMQELSRCRVLRGHPKIFLLQACRGGNRDAGVGPTALPWYRRWLRAPPSIPSHADVLQIYAEAQDSSGRGPPSGSSHQADILTVYAAAEGYVAYRDEKGSDFIQTLVEVLRANPGRDLLELLTEVNRRVCELDVLGPDCDEPRKACLEICSSLRRRLCLQA

>XP_009007271.3 **CASP16 [*Callithrix jacchus*]**

MAFLVAGTLRVATQIADTRDSLGRKGKYNVQGPRAALMLSSPGVADAAVTALKDVFQALGFESFERREVPVQGFLEELAWFRERLDAQGHLLGCALVALVAPRGQLWHPQQLVRELSSCGVLQGCPKVFLLLPSGPGATLEPGAFLDGLRELCSRSPHWSLVQLLTELFHRVAEESTGGTCCPVLQSSLRGALCLGGMEPWRPEPAPGPSTQYDLSKTRAALLLAVIRGRPGAQHDVEALGGLCRALGFETTVRTNPTAQAFQEELAQFREQLDTCRGPVSCALVALMAHGGPQGQLLGADGQEVQPQALMQELSRCQMLRGHPKIFLLQACRGGNRDAGVGPAALPWYRRWLWAPPSVPSHADVLQIYAEARDSSSRGPPSGSSHQADILTVYAAAEGYVAYRDERGSDFIQTLVEVLRANPGRDLLELLTEVNRRVCEQDVLGPDCDEPRKACLEICSSLRHRLCL

>XP_012599027.1 **CASP16 [*Microcebus murinus*]**

MAFLVAGTLQVASQIADAQDSLGRKGKYSVQGPRVALTLSSPGVSAAAVAALEGVFQALGFDTCKRREASVQGFLEELVWFREWLDAHGGPVGCALVALVAPSGQLRQPQQLVQELSCCEALRGCPKVFLLLSSGPGATLEPEAFLSGLRELCGRFPHWSLVQLLTELFCRVAVESTEGTYCPILRSSLRGTLCLGGVEPWMPEPVPGPNTHYDLSRARAALLLAVIRGRPGAQHDVEALRGFCQALGFETTLRTDPTAQAFQEELAQFRERLDTRSGPVSCALVALMAHGGPRGQLIGADGQEVQPEVLKQELSHCRVLRGCPKIFLLQACRGGNKDAGAGPTALPWYWRWLRAPPAIPSHADVLQIYADAQGSASKGSLPGSSDQADILTVYAAAEGYVAYRDDKGSDFIQTLVEVLRAKPGGDLLELLTEVNRRVCEQDVLGPDCNELRKACLEIRSSLRRRLCLQA

>XP_008564539.1 **CASP16 [*Galeopterus variegatus*]**

MAFLVAGTLRVAAQMADAQDSLGRKGKYSLQGPRVALILSSPGVPTAMVAALGGVFQALGFESCERRESPVQGFLEELTWFREYLDARGGPMGCALVALVAPSGQLRQPQRLVQELSCCGALRGCPKVFLLLSAGHGAAPEPGAFLACLGELCGRSHHWSLLQLLTELFCRVAEESTGGTRCPILRSSLRGALFLGGVEPRGPEPEPSLSAQYDLSGARAALLLAVTQDRPGAQYDLEVLGGLCQALGFETTLRTDPTAQAFQEELAQFREQLDTRRGPVSCALVALMAHGGPRGQLLGADGQEAQPEALVRELSRCQALRGCPKIFLLQACRGGNRDTGVGLTALPWYWCWLRAPATIPSHADILQIYADAPGSSSGSPTPGSSDQADILSVYAATEGCVAYRDEKGSDFIQTLVEVLRADPGQDLLELLTEVNRRVCELDVLGPDCDQLRKACLEIRSSLRRRLCLRA

>XP_008248858.1 **CASP16 [*Oryctolagus cuniculus*]**

MAFLVAGTLQAAAQVADARDSLGRKGKYSVQGLRVALTLGSPGVPAATVAALEGVFQALGFESWERREALVQDFLRELALFRKQLDALQSPVGCALVALVAPRGQLRQPRQLVQELSCCEALQGCPKVVLLLSSGAGAALEPGAFLAGLRELCGRRPHWSLLQLLTELFCRVAEDSAGDIRCPVFQSSLRGALCLTSEEPWRPEAEPGPGAQYDMSGARAALLLAVIRDRPGAERDVEALGGLCQALGFETTLRTDPTAQAFWEELAQFQEQLDTYRGPVSCALVALMAHGGPQGQLLGADGQEVQPEALVQELSRCGALSGCPKVFLLQACRGGNRDAGKGPKALPWYRRWLWVPQPALPSHADVLQVHADAQGSSCSGPAPGSSGQADVLTVYAAAEGCVAYRDEMGSDFIQTLVDVLRADPGRDLLELLTEVNRRVCELDVLGPDCDEPRKACLEIRSSLRRRLRLRV

>XP_063126149.1 **CASP16 [*Rattus norvegicus*]**

MAFLVSGTLQAAAQLVDAWDSLGRKGKYSVQGSRAALILCSPGVSARAVAAMDGVLQALGFENYKKRQVLVQSFLEELGLFREQLDVQGVPVGCALVVLMAPSGQLRQPQLLVKELSHCGSLQGCPKIFLLLSSGLKAAWEPEAFLLHLGKICSQHPHWSLLQLLTELFCRIAEESAENTYCPIFRTSFRGTLCLGDGPWRPESDPGPSTQYDLSGTRAALLLAVFQDRLGARHDVTALRDLCQALGFKVTLRTNPSAQAFREELAEFRKKLDTHKGPVSCALVALMAHGGPQGQLLGADGEEVQPEVLVQELSCCQALHGHPKIFLFQACRGGYRDPGVGPRALPWYRHWLRAPPAIPTQADVLQIHADAPGSLLSTPGGSGQADILTVYAAAEGCVAYRDEKGSDFVQTLVEVIRANPGRDLLELMTEVNRRVCELDVLGPDSDELRKACLEIRSSLRRRLCL

>XP_039741050.1 **CASP16 [*Pteropus giganteus*]**

MAFLVSGTLQVAAQMADARESLRRKGKYNLQGPRVALTLSSPEVSASTVAVLESVFQTLGFQSCQRRKASVQDFHGELTGFREQLDAHRGPVGCALVALVAPSGQLQQLRPLVWELSHCGALQGCPKVFLLLSSIPGAAPKPGAFLTGLNELCGRCPHWSLLQLLTEVFCRTAEEYAGATYCPVLRSSLRGALCLGDVEPWGPEPEPSPSTQYDLSGAKAALLLAVIRDRSGAQHDVKALGGLCQALGFKTTLRTNPTAQAFQEELAQFREQLDTHRGPVSCALVALMAHGGPQGQLLGADGQEVKPEALVQELSHCRALRGCPKIFLLQACRGGHRDAGMGPTALPWFWRWLQAPPAIPSHADILQIYVNHQGSSSRDLTPGSSNQADILTVYAAAEGCVAYRDEKGSDFIQTLVEVLRTAPQGDLLELLTEVNRRVCELDVLGPDCDERRKACLEIHSSLRHRLCLQA

>XP_005873055.2 **CASP16 [*Myotis brandtii*]**

MAFLVSGTLRVAAQIADAQDSLGRKGKYSMRGPRVALTLSSPEVSASTVAILASVFQTLGFENCQRREASVQDFLEELTGFREQLDARGAPMGCVLVALVAPNGQLRQPQKLVRELSGCEALRGCPKVFLLLSSAPGAPPKPGTFLTGLSELCGRCPRWSLLQLLTEVFCRTAGESSGATYCPVLQSSLRGALCLGDMDPWGPEPEPSPGAQYDLSRDRAALCLAVIRDRPGAQRDVEALGGLCQTLGFETTLRTDPTAQAFQEELAQFQERLDTRKSPVSCALVALMAHGGPQGQLLGADGKEVRPEALVQELSHCRALRGCPKIFLLQACRGGHRDAGMGPTALPWFWRWLRAPPATPSHADVLRIYADAQGNSSGGFTPGSCDQADILMVYAAAEGCVAYRDEKGSDFIQTLVEVLRATPEGDLLELLTEVNRQVCELDVRGPDCNERRKACLEIRSSLRRRLCLQA

>XP_017512178.2 **CASP16 [*Manis javanica*]**

MAFLVAGTLQVASQMADAQESLERKGKYSVRGLRVALTLSSPEVSASTVAVLDGVFRALGFESCRRREASVQGFPGELAGFLEQLHAHRGPVGCALVAAVAPRGQLRRPRQLVRELSCCGALRGCPKVFLLLSSTPGAAPEPGAFLSGLQELCGRSPHWPLLQLLTEVFCRTAEETSGTTYCPVLRSSLRGALCLGDVGPWGPEPAPGPSAQYDLSGARAALLLAVIQGRPGARHDVEALGGLCEALGFETTLRTDPTAQAFLEELAQFRDRLDTHRGPVSCALVALMAHGGPQGQLLAVDGQAVWPEALVQELSGCRALRGCPKIFLLQACRGGHRDAGTGPTLLPWFQRWLRAPPATSSHADVLQIYADAQGGICSGPTPGSCDQADILTVYATAEGCVAYRDEKGSDFIQTLVEVLRAEPQGDLLELLTEANRQVCELDVLGPDSDERRKACLEIRSSLRRRLCLQA

>XP_038396551.1 **CASP16 [*Canis lupus familiaris*]**

MAFLVAGTLQVASLLADARQSLERKGKYSLQGPRVALTLSTPEVSASTVAVLEAVFRTLGFECCQRTEASVQGFLGELAGFRSQLDGLGGPVGCALVALLAPRGQLGQPQQLVRELSRCRALWGRPKVFLLLSSAPGGALERGAFLTGLSRLCGRCRHWSLLQLLTEVFYRTTEESEATYCPVFRSSLRGTLCLGDVEPWEPKLEPSPRAQYDLSGTRAALLLSVIRSRPGAKHDVEALGSLCQALNFKITLRTNPTAQAFQEEMVQFRECLDALSAPVSCALVALMAHGGPQGQLLGADGQEVQPEALVQELNRCRALWGCPKIFLLQACRGGHRDAGVGPTALSWFRRWLRASPTTPSHADVLEIYTDAQGSASRGPTAGSSDQADILMVYAAAEGCVAYRDEKGSDFIQTLAEVLRADPGGDLLELLTEVNRRVCELDVLGPDCPERRKACLEIRSSLRRPLCLQA

>XP_026341365.2 **CASP16 [*Ursus arctos*]**

MAFLVAGTLQVASKMADARQSLERKGKYGLQGPRVALTLCSPEVSASTVAVLEGVFRTLGFESCQRREASVQGFLGELAGFREQLDALGGPVGCALVALVAPRGQLRQPQQLVQELSHCRALWGCPKVFLLLSSAPGAASEPGAFLTVLSELCGRFPHWSLLQLLTEVFYRTAKESEATYCPVLWSSLRGALFLGDVEPWEPQLEPSPSTQYDLSGTRAALLLSVVHDRPGAQHDVEALGGLCRALSFKTTLRTDPTAQAFQEEMAQFRECLDARRAPVSCALVALMAHGGPQGQLLGADGQEVQPEALVQELSHCRALWGCPKIFLLQACRGGQRDAGVGPTALPWFRRWLRASPTTPSHADVLQIYADAQGSSSAGPAPGSPDQADVLMVYAAAEGCVAYRDEEKGSDFIQTLVEVLRADPGAELLELLTEVNRRVCELEVLGPDCQERVKACLEIRSSLRRQLCLQA

>XP_003998970.4 **CASP16 [*Felis catus*]**

MAFLVAGTLQVASQIADARESLGRKGKYSMRGPRVALTLSSPEVSASIVAVLEGVFRTLGFESCQRREASVQGFLGELAAFREQLDALGGPVGCALVALVAPSGQLKQPQQLVRELSRCGALWGRPKVFLLLSSAPGAAPEPGAFLTGLSELCGRFPHWSLLQLLTEVFCRTAEESEATYCPVLRSSLRGALCLGDAEPWGPEPEPSPTARYDLSGTRAALLLSVIHGRPGARHDVEALGTLCQALSFKTTLRTDPTAQAFQEELAQFRECLDAHRAPVSCALVALMAHGGPQGQLLGADGREVRPEALVQELSRCRALCGCPKIFLLQACRGGHRDAGVGPAGLPWFRRWLRASPTTPSHADVLQIYADAQGSSPFPTAGSSDQADILTVYAAAEGCVAYRDETGSDFIQTLVEVFRADPGREVLELLTEVNRRVCQLEVLGPDCPERRKACLEIRSSLRRRLCLQA

>XP_005224700.2 **CASP16 [*Bos taurus*]**

MAFLVAGTLQVASQVADAQESLGRKGKYSVKDPRVALALCSPEVSASTAALLEGVFQTLGFESCRRQGASVQGFRGELTRFREQLDAHGGSVGCAFVALVARPWQLRQSQQLVRELSRCKALWGRPKVFLLLSSAPGAVPEPGAFLASLGELCGRRPHWSLLQLLTEVFSRTAEESAGAAYCPVLRSSLRGALCLGNVEPWGPEPEPGPSAQYDLSGARAALLLAVTQGRLGAQHDVEALEGLCQALGFETTLRTDPTAQTFQEEMAQFRKRLDAHRGPVSCALVALMAHGGPQGQLLGADGQERQLEVLVQELSHCGALRGRPKIFLLQACRGGHRDAGVGPAALPWFRRWLRAPPATPSQADVLHVCTDVQGRSSRGPTPRSPNQADVLMVYAAAEGCVAYRDKKGSDFIQTLVEVLRADPKADLLELMTEVNRQVCELDVLGPDCDDRRKACLEIRSSLRRRLCLQV

>XP_013851189.1 **CASP16 [*Sus scrofa*]**

MAFLVAGTLRAASQMADARESLGRKGKYRVQGPRVALTLSSPEVSASTVAILEGVFRTLGFTSCQRREASAQGFRGELARFREQLDAHRGPVGCVLVALVAPREQLRQAQQLVWELSRCEALWGRPKVFLLLSGAPAAAPEPGTFLTELGELCGRCPRQPLLQLLAEVFCRTAEESPGATYCPVLRSSLRGALCLGDEELWGPEPEASPCAQYDLSGARAALLLAVIQDRPGALRDVEALGDLCQALGFETTLKMDPTAQVFQEEVARFREQLDTRRGPVSCALVALMAHGGPKGQLLGADGQEVQPEVLVQELSCCGALGGRPKIFLVQACRGGHRDAGVGPTALPWFWRWLRAPPAIHSRADVLQIYADVQGSLSRDPTAGRSNPADLLTVYAAAEGCVAYRDERGSDFIQTLVEVLRADPGRDLLELMTEVNRQVCELDVLGPDCEERRKACLEICSSLRRRLCLQA

>XP_006874042.1 **CASP16 [*Chrysochloris asiatica*]**

MAFLVAGTLRVAVQMADIRESLGRKGRYSVLHPRVALMLSSPGVPAVTIAALEGVLRALGFESLEQKVVPVQSFSEELVQFREQLDAQGDPISCTLVALVTLRGQLRWPLKLAQELNGCGVLWGCPKLFLLLSIAPGVTPESGSFLHHLAELCGHCPHWSLLQLLTEVFRRVTESAGGTFCPVLRSSLRGALCLGNVQAWESELDSGLKAQYDLSGVRVALLLGMIRGRPGAQCDMEALGSLCQTLGFETTVRTDPTAQAFQEELAKFQEQLTTCRDPVSCALVALMAHGGPQGRLLGADGQEVQPEALLLELSRCEALQGCPKIFLLQACRGGHRDLGVATTVSWFWRRLCSRPPTIPSHADVLQVYADVQGSSFRGHPPRTADHADILMVYSAAEGCVAYRDDKGSDFIQTLVEVVGADPGRDLLELMTEVNRQVCELDVLGPDSDKPHKACLEIRSSLRRSLCLRA

>XP_045141910.1 **CASP16 [*Echinops telfairi*]**

MAFLVAGTLRVAAQMADTWDSLARMGRYNMLGPKVALMLSSPGVPATTVTALEGLLQALGFERPEQREASLQDFLEEVARFREQLDTLGSPIGCALVALVIPRGQLRRPQRLAQELSHCGALQGRPKLLLLLTSAPGATAEPGTFLQGLAELSGCCPHWSLMRLLTEVFHRVTEDPTGTIPCPVLRSSLRGALYLGDMQAKRPELVSSPGAQYDLSGARVALLLAVLQGRPGAQCDMAVLEDLCQTLGFETTVRTDPTAQAFQEEVTQFREQLDARTGPVSCALVALMAHGGRQGQLLGTDGQEVHPEALVLELSRCGVLQGCPKIFLLQACRGGHRDSGMGPAAFSWFWSWLRAPPMVPSHADLLQVYADVQGSSSGVPRPGSADHADILTVYAAAEGCVAYRDEKGSDFIQTLVEVLRADSGGDLLELMTEVNRRMCELAVLGPDCDQPRKECLELRSSLRRRLCLRA

>XP_016279603.1 **CASP16 [*Monodelphis domestica*]**

MSFLAAGSLQVAVEKAEGRVCLEKKGDYDTSGARVALTLCATEGQAGEDRIIAALEAMYQVMSCKSYLRRVVKAQSFQEEMASFRENLDDRGSSMNCALVALVAHSERPGWLLGPDGKEVQEKELVRELNHCQALWGKAKVFLLLDIHNTDLGSIAFLSTLTDVCRHFPHWHLLEVLTQVIGKVTQEMPPTGHRCPIFQSSLRGALYLGRRRSQGLELSPIPQGVYDTSGAKVALVLCVTRDRPGAKQDLKALKRLFQTLGFKSILKMNPTAQDFRKELTRFRELLDARRTNVSCALVALMAHGEPQGRLLGADGQMVEVEEMVSELSACQVLQGKAKVVLLQGCRGGNRDPGMRPRALPWLGSWLQHWLQRPSTIPSHADILQVYANLQDVSSKGYSPKNPDQVDILRVYSAAEGYVAYRNENGSDFIQTLVQVIIANPDQDLLELLTEVNKRLCEMEVRGPDCDEIRKMSVEIQSSLRKQLYLKSQLSN

>XP_038609296.1 **CASP16 [*Tachyglossus aculeatus*]**

MRTRATAQAVGGVIGETAPPGKSCPAFRSSLRGMLCLGRRDVEVTGGDSEPCTSNRGGGPAPQEEYDMSGARVALTLCVFRDRAGAERDVEALERLCRTLGFESSVRRDPTAQDFRDEMAQFRAKLDGRGAPVSCALVTFMAHGGRGGRLLGADGQEVEPEDLIAELLPCRALSGGVKLFLLQSCRGGQRDSGAGAPGFPWLRRWLRGPPAIPSHADILRVYGDVRGRSSMPAPSGLDQADTLRVYAAADGCVAYRDEQGSDFIQTVVEVLLAAPPHRDLLDLLTEVNRKMCEADVLGPNSDDICKMNLEIQSSLRKRLCLQAPLRLITGKGESPRGRTPTQDEL

**C**

CLUSTAL multiple sequence alignment by MUSCLE (3.8)

XP_006874042.1 MAFLVAGTLRVAVQMADIRESLGRKGRYSVLHPRVALMLSSPGVPAV---TIAALEGVLR

XP_045141910.1 MAFLVAGTLRVAAQMADTWDSLARMGRYNMLGPKVALMLSSPGVPAT---TVTALEGLLQ

XP_063126149.1 MAFLVSGTLQAAAQLVDAWDSLGRKGKYSVQGSRAALILCSPGVSAR---AVAAMDGVLQ

XP_008248858.1 MAFLVAGTLQAAAQVADARDSLGRKGKYSVQGLRVALTLGSPGVPAA---TVAALEGVFQ

XP_005224700.2 MAFLVAGTLQVASQVADAQESLGRKGKYSVKDPRVALALCSPEVSAS---TAALLEGVFQ

XP_008564539.1 MAFLVAGTLRVAAQMADAQDSLGRKGKYSLQGPRVALILSSPGVPTA---MVAALGGVFQ

XP_012599027.1 MAFLVAGTLQVASQIADAQDSLGRKGKYSVQGPRVALTLSSPGVSAA---AVAALEGVFQ

XP_064218437.1 MAFLVAGTLRVAMQMADTRDSLERKGKYNVQGPRAALMLSSPGVAAA---AVTALKDVFQ

XP_009007271.3 MAFLVAGTLRVATQIADTRDSLGRKGKYNVQGPRAALMLSSPGVADA---AVTALKDVFQ

XP_021787375.1 MAFLVAGTLRVATQMADTRDSLGRKGKYDVRGPKAALMLISPGVVAA---AVTALEDVFQ

XP_014980933.2 MAFLVAGTLRVATQMADTRDSLGRKGKYDVRGPKAALMLISPGVVAA---AVTALEDVFQ

XP_005591104.3 MAFLVAGTLRVATQMADTRDSLGRKGKYDVRGPKAALMLISPGVVAA---AVTALEDVFQ

XP_523278.4 MAFLVAGTLRLAMQMADTRDSLGRKGKYDVQGPRAALMLSSPGVAAA---VVTALEDVFQ

XP_004057200.3 ------------MQMADTRDSLGRKGKYDVQGPRAALMLSSPGVAAA---VVTALEDVFQ

XP_013851189.1 MAFLVAGTLRAASQMADARESLGRKGKYRVQGPRVALTLSSPEVSAS---TVAILEGVFR

XP_005873055.2 MAFLVSGTLRVAAQIADAQDSLGRKGKYSMRGPRVALTLSSPEVSAS---TVAILASVFQ

XP_039741050.1 MAFLVSGTLQVAAQMADARESLRRKGKYNLQGPRVALTLSSPEVSAS---TVAVLESVFQ

XP_017512178.2 MAFLVAGTLQVASQMADAQESLERKGKYSVRGLRVALTLSSPEVSAS---TVAVLDGVFR

XP_038396551.1 MAFLVAGTLQVASLLADARQSLERKGKYSLQGPRVALTLSTPEVSAS---TVAVLEAVFR

XP_003998970.4 MAFLVAGTLQVASQIADARESLGRKGKYSMRGPRVALTLSSPEVSAS---IVAVLEGVFR

XP_026341365.2 MAFLVAGTLQVASKMADARQSLERKGKYGLQGPRVALTLCSPEVSAS---TVAVLEGVFR

XP_016279603.1 MSFLAAGSLQVAVEKAEGRVCLEKKGDYDTSGARVALTLCATEGQAGEDRIIAALEAMYQ

XP_038609296.1 ------------------------------------------------------------

XP_006874042.1 ALGFESLEQKVVPVQSFSEELVQFREQLDAQGDPISCTLVALVT----------LRGQLR

XP_045141910.1 ALGFERPEQREASLQDFLEEVARFREQLDTLGSPIGCALVALVI----------PRGQLR

XP_063126149.1 ALGFENYKKRQVLVQSFLEELGLFREQLDVQGVPVGCALVVLMA----------PSGQLR

XP_008248858.1 ALGFESWERREALVQDFLRELALFRKQLDALQSPVGCALVALVA----------PRGQLR

XP_005224700.2 TLGFESCRRQGASVQGFRGELTRFREQLDAHGGSVGCAFVALVA----------RPWQLR

XP_008564539.1 ALGFESCERRESPVQGFLEELTWFREYLDARGGPMGCALVALVA----------PSGQLR

XP_012599027.1 ALGFDTCKRREASVQGFLEELVWFREWLDAHGGPVGCALVALVA----------PSGQLR

XP_064218437.1 ALGFESCERREVPVQGFLKELAWFRERLDAHGCLVGCALVALVA----------PRGQLW

XP_009007271.3 ALGFESFERREVPVQGFLEELAWFRERLDAQGHLLGCALVALVA----------PRGQLW

XP_021787375.1 ALGFESWERRQVPVQGFLEKLAWFRELLDAHRHPVGCALVALVA----------PKGQLK

XP_014980933.2 ALGFESWERRQVPVQGFLEKLAWFRERLDAHRHPVGCALVALVA----------PRGQLK

XP_005591104.3 ALGFESWERRQVPVQGFLKKLAWFRERLDAHRHPVGCALVALVA----------PRGQLK

XP_523278.4 ALGFESCARREVPVQGFLEELAWFQEQLDAHGRPVGCALVALVA----------PRGQLR

XP_004057200.3 ALGFESCKRREVPVQGFLEELACFQEQLDAHGRPVGCALVALVA----------PRGQLR

XP_013851189.1 TLGFTSCQRREASAQGFRGELARFREQLDAHRGPVGCVLVALVA----------PREQLR

XP_005873055.2 TLGFENCQRREASVQDFLEELTGFREQLDARGAPMGCVLVALVA----------PNGQLR

XP_039741050.1 TLGFQSCQRRKASVQDFHGELTGFREQLDAHRGPVGCALVALVA----------PSGQLQ

XP_017512178.2 ALGFESCRRREASVQGFPGELAGFLEQLHAHRGPVGCALVAAVA----------PRGQLR

XP_038396551.1 TLGFECCQRTEASVQGFLGELAGFRSQLDGLGGPVGCALVALLA----------PRGQLG

XP_003998970.4 TLGFESCQRREASVQGFLGELAAFREQLDALGGPVGCALVALVA----------PSGQLK

XP_026341365.2 TLGFESCQRREASVQGFLGELAGFREQLDALGGPVGCALVALVA----------PRGQLR

XP_016279603.1 VMSCKSYLRRVVKAQSFQEEMASFRENLDDRGSSMNCALVALVAHSERPGWLLGPDGKEV

XP_038609296.1 ---------------------MRTRATAQAVGGVIGETA---------------PPGKS-

XP_006874042.1 WPLKLAQELNGCGVLWGCPKLFLLLSIAPGVTPESGSFLHHLAELCGHCPHWSLLQLLTE

XP_045141910.1 RPQRLAQELSHCGALQGRPKLLLLLTSAPGATAEPGTFLQGLAELSGCCPHWSLMRLLTE

XP_063126149.1 QPQLLVKELSHCGSLQGCPKIFLLLSSGLKAAWEPEAFLLHLGKICSQHPHWSLLQLLTE

XP_008248858.1 QPRQLVQELSCCEALQGCPKVVLLLSSGAGAALEPGAFLAGLRELCGRRPHWSLLQLLTE

XP_005224700.2 QSQQLVRELSRCKALWGRPKVFLLLSSAPGAVPEPGAFLASLGELCGRRPHWSLLQLLTE

XP_008564539.1 QPQRLVQELSCCGALRGCPKVFLLLSAGHGAAPEPGAFLACLGELCGRSHHWSLLQLLTE

XP_012599027.1 QPQQLVQELSCCEALRGCPKVFLLLSSGPGATLEPEAFLSGLRELCGRFPHWSLVQLLTE

XP_064218437.1 QPQQLVRELSSCGVLQGCPKVFLLLPSGPGATLEPGAFLDGLRELCGRSPHWSLVQLLTE

XP_009007271.3 HPQQLVRELSSCGVLQGCPKVFLLLPSGPGATLEPGAFLDGLRELCSRSPHWSLVQLLTE

XP_021787375.1 QPQQLVRELSGCGALRGCPKVFLLLSSGPGSALESGAFLAGLRELCGRSPHWCLVQLLTE

XP_014980933.2 QPQQLVRELSGCGALRGCPKVFLLLSSGPGSALESGAFLAGLRELCGRFPHWSLVQLLTE

XP_005591104.3 QPQQLVRELSGCGALRGCPKVFLLLSSGPGSALESGAFLAGLRELCGRFPHWSLVQLLTE

XP_523278.4 QPQQLVRELSGCRALRGCPKVFLLLSSGPGSSLEPGAFLAGLRELCGRSPHWSLVQLLTE

XP_004057200.3 QPQQLVRELSGCRALRGCPKVFLLLSSGPGSSLEPGAFLAGLRELCGCSPHWSLVQLLTE

XP_013851189.1 QAQQLVWELSRCEALWGRPKVFLLLSGAPAAAPEPGTFLTELGELCGRCPRQPLLQLLAE

XP_005873055.2 QPQKLVRELSGCEALRGCPKVFLLLSSAPGAPPKPGTFLTGLSELCGRCPRWSLLQLLTE

XP_039741050.1 QLRPLVWELSHCGALQGCPKVFLLLSSIPGAAPKPGAFLTGLNELCGRCPHWSLLQLLTE

XP_017512178.2 RPRQLVRELSCCGALRGCPKVFLLLSSTPGAAPEPGAFLSGLQELCGRSPHWPLLQLLTE

XP_038396551.1 QPQQLVRELSRCRALWGRPKVFLLLSSAPGGALERGAFLTGLSRLCGRCRHWSLLQLLTE

XP_003998970.4 QPQQLVRELSRCGALWGRPKVFLLLSSAPGAAPEPGAFLTGLSELCGRFPHWSLLQLLTE

XP_026341365.2 QPQQLVQELSHCRALWGCPKVFLLLSSAPGAASEPGAFLTVLSELCGRFPHWSLLQLLTE

XP_016279603.1 QEKELVRELNHCQALWGKAKVFLLLDI-HNTDLGSIAFLSTLTDVCRHFPHWHLLEVLTQ

XP_038609296.1 ------------------------------------------------------------

XP_006874042.1 VFRRVT-E-SAGGTFCPVLRSSLRGALCLG--NVQAWESELDSG---------LKAQYDL

XP_045141910.1 VFHRVTED-PTGTIPCPVLRSSLRGALYLG--DMQAKRPELVSS---------PGAQYDL

XP_063126149.1 LFCRIAEE-SAENTYCPIFRTSFRGTLCLG--D-GPWRPESDPG---------PSTQYDL

XP_008248858.1 LFCRVAED-SAGDIRCPVFQSSLRGALCLT--SEEPWRPEAEPG---------PGAQYDM

XP_005224700.2 VFSRTAEE-SAGAAYCPVLRSSLRGALCLG--NVEPWGPEPEPG---------PSAQYDL

XP_008564539.1 LFCRVAEE-STGGTRCPILRSSLRGALFLG--GVEPRGPEPEPS---------LSAQYDL

XP_012599027.1 LFCRVAVE-STEGTYCPILRSSLRGTLCLG--GVEPWMPEPVPG---------PNTHYDL

XP_064218437.1 LFHRVAEE-STGGTCCPVLQSSLRGALCLG--GVEPWRPEPATG---------PSTQYDL

XP_009007271.3 LFHRVAEE-STGGTCCPVLQSSLRGALCLG--GMEPWRPEPAPG---------PSTQYDL

XP_021787375.1 LFRKVTEE-STGGTCCPVLRSSLRGALCLG--GVEPWRPELAPG---------PNTQYDL

XP_014980933.2 LFRKVTEE-STGGTCCPVLRSSLRGALCLG--GVEPWRPELAPG---------PNTQYDL

XP_005591104.3 LFRKVTEE-STGGTCCPVLRSSLRGALCLG--GVEPWRPELAPG---------PNTQYDL

XP_523278.4 LFRRMAEE-SAGGTCCPVLRSSLRGALCLG--GVEPWRPEPAPG---------PSTQYDL

XP_004057200.3 LFHRVAEE-SAGGTCCPVLRSSFRGALCLG--GVEPWRPEPAPS---------PSTQYDL

XP_013851189.1 VFCRTAEE-SPGATYCPVLRSSLRGALCLG--DEELWGPEPEAS---------PCAQYDL

XP_005873055.2 VFCRTAGE-SSGATYCPVLQSSLRGALCLG--DMDPWGPEPEPS---------PGAQYDL

XP_039741050.1 VFCRTAEE-YAGATYCPVLRSSLRGALCLG--DVEPWGPEPEPS---------PSTQYDL

XP_017512178.2 VFCRTAEE-TSGTTYCPVLRSSLRGALCLG--DVGPWGPEPAPG---------PSAQYDL

XP_038396551.1 VFYRTTEE--SEATYCPVFRSSLRGTLCLG--DVEPWEPKLEPS---------PRAQYDL

XP_003998970.4 VFCRTAEE--SEATYCPVLRSSLRGALCLG--DAEPWGPEPEPS---------PTARYDL

XP_026341365.2 VFYRTAKE--SEATYCPVLWSSLRGALFLG--DVEPWEPQLEPS---------PSTQYDL

XP_016279603.1 VIGKVTQEMPPTGHRCPIFQSSLRGALYLG--RRRSQGLELSPI---------PQGVYDT

XP_038609296.1 ---------------CPAFRSSLRGMLCLGRRDVEVTGGDSEPCTSNRGGGPAPQEEYDM

** :.:*:** * * . . **

XP_006874042.1 SGVRVALLLGMIRGRPGAQCDMEALGSLCQTLGFETTVRTDPTAQAFQEELAKFQEQLTT

XP_045141910.1 SGARVALLLAVLQGRPGAQCDMAVLEDLCQTLGFETTVRTDPTAQAFQEEVTQFREQLDA

XP_063126149.1 SGTRAALLLAVFQDRLGARHDVTALRDLCQALGFKVTLRTNPSAQAFREELAEFRKKLDT

XP_008248858.1 SGARAALLLAVIRDRPGAERDVEALGGLCQALGFETTLRTDPTAQAFWEELAQFQEQLDT

XP_005224700.2 SGARAALLLAVTQGRLGAQHDVEALEGLCQALGFETTLRTDPTAQTFQEEMAQFRKRLDA

XP_008564539.1 SGARAALLLAVTQDRPGAQYDLEVLGGLCQALGFETTLRTDPTAQAFQEELAQFREQLDT

XP_012599027.1 SRARAALLLAVIRGRPGAQHDVEALRGFCQALGFETTLRTDPTAQAFQEELAQFRERLDT

XP_064218437.1 SKTRAALLLAVIQGRPGAQHDVEALGGLCRALGFETTVRTDPTAQAFQEELAQFREQLDT

XP_009007271.3 SKTRAALLLAVIRGRPGAQHDVEALGGLCRALGFETTVRTNPTAQAFQEELAQFREQLDT

XP_021787375.1 SKTRAALLLAVIQGRPGAQHDVEALGGLCRALGFETTVRTDPTAQAFQEELAQFQKQLDT

XP_014980933.2 SKTRAALLLAVIQGRPGAQHDVEALGGLCRALGFETTVRTDPTAQAFQEELAQFQKQLDT

XP_005591104.3 SKTRAALLLAVIQGRPGAQHDVEALGGLCRALGFETTVRTDPTAQAFQEELAQFQKQLDT

XP_523278.4 SKARAALLLAVIQGRPGAQHDVEALGGLCRALGFETTVRTDPTAQAFQEQLAQFREQLDT

XP_004057200.3 SKARAALLLAVIQGRPGAQHDVEALGGLCRALGFETTVRTDPTAQAFQEELAQFREQLDT

XP_013851189.1 SGARAALLLAVIQDRPGALRDVEALGDLCQALGFETTLKMDPTAQVFQEEVARFREQLDT

XP_005873055.2 SRDRAALCLAVIRDRPGAQRDVEALGGLCQTLGFETTLRTDPTAQAFQEELAQFQERLDT

XP_039741050.1 SGAKAALLLAVIRDRSGAQHDVKALGGLCQALGFKTTLRTNPTAQAFQEELAQFREQLDT

XP_017512178.2 SGARAALLLAVIQGRPGARHDVEALGGLCEALGFETTLRTDPTAQAFLEELAQFRDRLDT

XP_038396551.1 SGTRAALLLSVIRSRPGAKHDVEALGSLCQALNFKITLRTNPTAQAFQEEMVQFRECLDA

XP_003998970.4 SGTRAALLLSVIHGRPGARHDVEALGTLCQALSFKTTLRTDPTAQAFQEELAQFRECLDA

XP_026341365.2 SGTRAALLLSVVHDRPGAQHDVEALGGLCRALSFKTTLRTDPTAQAFQEEMAQFRECLDA

XP_016279603.1 SGAKVALVLCVTRDRPGAKQDLKALKRLFQTLGFKSILKMNPTAQDFRKELTRFRELLDA

XP_038609296.1 SGARVALTLCVFRDRAGAERDVEALERLCRTLGFESSVRRDPTAQDFRDEMAQFRAKLDG

* ..** * : ..* ** *: .* : :*.*: :. :*:** * .::. *. *

XP_006874042.1 CRDPVSCALVALMAHGGPQGRLLGADGQEVQPEALLLELSRCEALQGCPKIFLLQACRGG

XP_045141910.1 RTGPVSCALVALMAHGGRQGQLLGTDGQEVHPEALVLELSRCGVLQGCPKIFLLQACRGG

XP_063126149.1 HKGPVSCALVALMAHGGPQGQLLGADGEEVQPEVLVQELSCCQALHGHPKIFLFQACRGG

XP_008248858.1 YRGPVSCALVALMAHGGPQGQLLGADGQEVQPEALVQELSRCGALSGCPKVFLLQACRGG

XP_005224700.2 HRGPVSCALVALMAHGGPQGQLLGADGQERQLEVLVQELSHCGALRGRPKIFLLQACRGG

XP_008564539.1 RRGPVSCALVALMAHGGPRGQLLGADGQEAQPEALVRELSRCQALRGCPKIFLLQACRGG

XP_012599027.1 RSGPVSCALVALMAHGGPRGQLIGADGQEVQPEVLKQELSHCRVLRGCPKIFLLQACRGG

XP_064218437.1 CRGPVSCALVALMAHGGPRGQLLGSDGQEVHPQALMQELSRCRVLRGHPKIFLLQACRGG

XP_009007271.3 CRGPVSCALVALMAHGGPQGQLLGADGQEVQPQALMQELSRCQMLRGHPKIFLLQACRGG

XP_021787375.1 CRGPVSCVLVALMAHGGPRGQLLGADGQEVQPEALMQELSRCRVLWGRPKVFLLQACRGG

XP_014980933.2 CRGPVSCVLVALMAHGGPQGQLLGADRQEVQPEALMQELSRCRVLWGHPKVFLLQACRGG

XP_005591104.3 CRGPVSCVLVALMAHGGPQGQLLGADRQEVQPEALMQELSRCRVLWGRPKVFLLQACRGG

XP_523278.4 CRGPVSCALVALMAHGGPRGQLLGADGQEVQPEALMQELSRCQVLQGRPKIFLLQACRGG

XP_004057200.3 CRGPVSCVLVALMAHGGPRGQLLGADGQEVQPEALMQELSCCQVLQGRPKIFLLQACRGG

XP_013851189.1 RRGPVSCALVALMAHGGPKGQLLGADGQEVQPEVLVQELSCCGALGGRPKIFLVQACRGG

XP_005873055.2 RKSPVSCALVALMAHGGPQGQLLGADGKEVRPEALVQELSHCRALRGCPKIFLLQACRGG

XP_039741050.1 HRGPVSCALVALMAHGGPQGQLLGADGQEVKPEALVQELSHCRALRGCPKIFLLQACRGG

XP_017512178.2 HRGPVSCALVALMAHGGPQGQLLAVDGQAVWPEALVQELSGCRALRGCPKIFLLQACRGG

XP_038396551.1 LSAPVSCALVALMAHGGPQGQLLGADGQEVQPEALVQELNRCRALWGCPKIFLLQACRGG

XP_003998970.4 HRAPVSCALVALMAHGGPQGQLLGADGREVRPEALVQELSRCRALCGCPKIFLLQACRGG

XP_026341365.2 RRAPVSCALVALMAHGGPQGQLLGADGQEVQPEALVQELSHCRALWGCPKIFLLQACRGG

XP_016279603.1 RRTNVSCALVALMAHGEPQGRLLGADGQMVEVEEMVSELSACQVLQGKAKVVLLQGCRGG

XP_038609296.1 RGAPVSCALVTFMAHGGRGGRLLGADGQEVEPEDLIAELLPCRALSGGVKLFLLQSCRGG

***.**::**** *.*:. * : : ** * * * *:.*.*.****

XP_006874042.1 HRDLGVA-TTVSW----FWRRLCSRPPTIPSHADVLQVYADVQGSSFRGHPPRTADHADI

XP_045141910.1 HRDSGMGPAAFSW----FWSWLRAP-PMVPSHADLLQVYADVQGSSSGVPRPGSADHADI

XP_063126149.1 YRDPGVGPRALPW----YRHWLRAP-PAIPTQADVLQIHADAPGSLL--STPGGSGQADI

XP_008248858.1 NRDAGKGPKALPW----YRRWLWVPQPALPSHADVLQVHADAQGSSCSGPAPGSSGQADV

XP_005224700.2 HRDAGVGPAALPW----FRRWLRAP-PATPSQADVLHVCTDVQGRSSRGPTPRSPNQADV

XP_008564539.1 NRDTGVGLTALPW----YWCWLRAP-ATIPSHADILQIYADAPGSSSGSPTPGSSDQADI

XP_012599027.1 NKDAGAGPTALPW----YWRWLRAP-PAIPSHADVLQIYADAQGSASKGSLPGSSDQADI

XP_064218437.1 NRDAGVGPTALPW----YRRWLRAP-PSIPSHADVLQIYAEAQDSSGRGPPSGSSHQADI

XP_009007271.3 NRDAGVGPAALPW----YRRWLWAP-PSVPSHADVLQIYAEARDSSSRGPPSGSSHQADI

XP_021787375.1 NRDAGVGPTALPW----YWSWLRAP-PSVPSHADVLQIYAEAQGSSCRGAPPGRSDQADI

XP_014980933.2 NRDAGVGPTALPW----YWSWLRAP-PSVPSHADVLQIYAEAQGSSCRGAPPGRSDQADI

XP_005591104.3 NRDAGVGPTALPW----YWSWLRAP-PSVPSHADVLQIYAEAQGSSCRGAPPGRSDQADI

XP_523278.4 NRDAGVGPTALPW----YWSWLRAP-PSVPSHADVLQIYAEAQGSSCRGTPPGSSDQADI

XP_004057200.3 NRDAGVGPTALPW----YWSWLRAP-LSVPSHADVLQIYAEAQGSSCRGTPPGSSDQADI

XP_013851189.1 HRDAGVGPTALPW----FWRWLRAP-PAIHSRADVLQIYADVQGSLSRDPTAGRSNPADL

XP_005873055.2 HRDAGMGPTALPW----FWRWLRAP-PATPSHADVLRIYADAQGNSSGGFTPGSCDQADI

XP_039741050.1 HRDAGMGPTALPW----FWRWLQAP-PAIPSHADILQIYVNHQGSSSRDLTPGSSNQADI

XP_017512178.2 HRDAGTGPTLLPW----FQRWLRAP-PATSSHADVLQIYADAQGGICSGPTPGSCDQADI

XP_038396551.1 HRDAGVGPTALSW----FRRWLRAS-PTTPSHADVLEIYTDAQGSASRGPTAGSSDQADI

XP_003998970.4 HRDAGVGPAGLPW----FRRWLRAS-PTTPSHADVLQIYADAQGSSPF-PTAGSSDQADI

XP_026341365.2 QRDAGVGPTALPW----FRRWLRAS-PTTPSHADVLQIYADAQGSSSAGPAPGSPDQADV

XP_016279603.1 NRDPGMRPRALPWLGSWLQHWLQRP-STIPSHADILQVYANLQDVSSKGYSPKNPDQVDI

XP_038609296.1 QRDSGAGAPGFPW----LRRWLRGP-PAIPSHADILRVYGDVRGRSSM-PAPSGLDQADT

.* * ..* . .* :.**:* : : . . .*

XP_006874042.1 LMVYSAAEGCVAYRD-DKGSDFIQTLVEV-VGADPGRDLLELMTEVNRQVCELDVLGPDS

XP_045141910.1 LTVYAAAEGCVAYRD-EKGSDFIQTLVEV-LRADSGGDLLELMTEVNRRMCELAVLGPDC

XP_063126149.1 LTVYAAAEGCVAYRD-EKGSDFVQTLVEV-IRANPGRDLLELMTEVNRRVCELDVLGPDS

XP_008248858.1 LTVYAAAEGCVAYRD-EMGSDFIQTLVDV-LRADPGRDLLELLTEVNRRVCELDVLGPDC

XP_005224700.2 LMVYAAAEGCVAYRD-KKGSDFIQTLVEV-LRADPKADLLELMTEVNRQVCELDVLGPDC

XP_008564539.1 LSVYAATEGCVAYRD-EKGSDFIQTLVEV-LRADPGQDLLELLTEVNRRVCELDVLGPDC

XP_012599027.1 LTVYAAAEGYVAYRD-DKGSDFIQTLVEV-LRAKPGGDLLELLTEVNRRVCEQDVLGPDC

XP_064218437.1 LTVYAAAEGYVAYRD-EKGSDFIQTLVEV-LRANPGRDLLELLTEVNRRVCELDVLGPDC

XP_009007271.3 LTVYAAAEGYVAYRD-ERGSDFIQTLVEV-LRANPGRDLLELLTEVNRRVCEQDVLGPDC

XP_021787375.1 LTVYSAAEGYVAYRD-DKGSDFIQTLVEV-LRANPGRDLLELLTEVNRRVCEQDVLGPDC

XP_014980933.2 LTVYSAAEGYVAYRD-DKGSDFIQTLVEV-LRANPGRDLLELLTEVNRRMCEQDVLGPDC

XP_005591104.3 LTVYSAVEGYVAYRD-DKGSDFIQTLVEV-LRANPGRDLLELLTEVNRRMCEQDVLGPDC

XP_523278.4 LTVYSAAEGYVAYRD-DKGSDFIQTLVEV-LRASPGRDLLELLTEVNRRMCEQEVLGPDC

XP_004057200.3 LTVYSAAEGYVAYRD-DKGSDFIQTLVEV-LRANPGRDLLELLTEVNRRVCEQEVLGPDC

XP_013851189.1 LTVYAAAEGCVAYRD-ERGSDFIQTLVEV-LRADPGRDLLELMTEVNRQVCELDVLGPDC

XP_005873055.2 LMVYAAAEGCVAYRD-EKGSDFIQTLVEV-LRATPEGDLLELLTEVNRQVCELDVRGPDC

XP_039741050.1 LTVYAAAEGCVAYRD-EKGSDFIQTLVEV-LRTAPQGDLLELLTEVNRRVCELDVLGPDC

XP_017512178.2 LTVYATAEGCVAYRD-EKGSDFIQTLVEV-LRAEPQGDLLELLTEANRQVCELDVLGPDS

XP_038396551.1 LMVYAAAEGCVAYRD-EKGSDFIQTLAEV-LRADPGGDLLELLTEVNRRVCELDVLGPDC

XP_003998970.4 LTVYAAAEGCVAYRD-ETGSDFIQTLVEV-FRADPGREVLELLTEVNRRVCQLEVLGPDC

XP_026341365.2 LMVYAAAEGCVAYRDEEKGSDFIQTLVEV-LRADPGAELLELLTEVNRRVCELEVLGPDC

XP_016279603.1 LRVYSAAEGYVAYRN-ENGSDFIQTLVQV-IIANPDQDLLELLTEVNKRLCEMEVRGPDC

XP_038609296.1 LRVYAAADGCVAYRD-EQGSDFIQTVVEVLLAAPPHRDLLDLLTEVNRKMCEADVLGPNS

* **::.:* ****: . ****:**:.:* . : . ::*:*:**.*..:*: * **:.

XP_006874042.1 DKPHKACLEIRSSLRRSLCLRA----------------------

XP_045141910.1 DQPRKECLELRSSLRRRLCLRA----------------------

XP_063126149.1 DELRKACLEIRSSLRRRLCL------------------------

XP_008248858.1 DEPRKACLEIRSSLRRRLRLRV----------------------

XP_005224700.2 DDRRKACLEIRSSLRRRLCLQV----------------------

XP_008564539.1 DQLRKACLEIRSSLRRRLCLRA----------------------

XP_012599027.1 NELRKACLEIRSSLRRRLCLQA----------------------

XP_064218437.1 DEPRKACLEICSSLRRRLCLQA----------------------

XP_009007271.3 DEPRKACLEICSSLRHRLCL------------------------

XP_021787375.1 DELRKACLEIRSSLRRRLCLQP----------------------

XP_014980933.2 DELRKACLEIRSSLRRRLCLQP----------------------

XP_005591104.3 DELRKACLEIRSSLRRRLCLQP----------------------

XP_523278.4 DELRKACLEIRSSLRRRLCLQA----------------------

XP_004057200.3 DELRKACLEIRSSLRRRLCLQA----------------------

XP_013851189.1 EERRKACLEICSSLRRRLCLQA----------------------

XP_005873055.2 NERRKACLEIRSSLRRRLCLQA----------------------

XP_039741050.1 DERRKACLEIHSSLRHRLCLQA----------------------

XP_017512178.2 DERRKACLEIRSSLRRRLCLQA----------------------

XP_038396551.1 PERRKACLEIRSSLRRPLCLQA----------------------

XP_003998970.4 PERRKACLEIRSSLRRRLCLQA----------------------

XP_026341365.2 QERVKACLEIRSSLRRQLCLQA----------------------

XP_016279603.1 DEIRKMSVEIQSSLRKQLYLKSQLSN------------------

XP_038609296.1 DDICKMNLEIQSSLRKRLCLQAPLRLITGKGESPRGRTPTQDEL

. * :*: ****. * *

**Supplementary Figure S2. Amino acid sequences of caspase-16 (CASP16) proteins of mammalian species.** **(A)** Caspase-16 proteins in GenBank. **(B)** The amino acid sequences (FASTA format) of CASP16 proteins of various mammalian species were downloaded from GenBank. Note that the amino acid sequence of gorilla CASP16 is partial, because the amino-terminal sequence is incomplete. **(C)** CLUSTAL multiple sequence alignment by MUSCLE (3.8). “*”, “:” and “.” below the sequences indicate identity, high similarity and low similarity of the residue in all sequences. Cysteine and histidine residues of the catalytic dyad are highlighted by yellow shading.

A  F  Q  E  Q  L  A  Q  F  R  E  Q  L  D  T  C  R

Chimpanzee CCACCCC**AG**GCTTTCCAGGAGCAGCTGGCCCAGTTCCGGGAGCAACTGGACACCTGCAGG

Echidna CCCCTCC**AG**GATTTTCGGGATGAGATGGCCCAGTTCCGGGCCAAGTTGGATGGCCGTGGG

Platypus cccctcc**ag**gattcccaggatgagatggcccagttccaggcagagttggatgcccgtggg

D S Q D E M A Q F Q A E L D A R G

G  P  V  S  C  A  L  V  A  L  M  A  H  G  G  P  R  G  Q  L

Chimpanzee GGCCCTGTGAGCTGTGCCCTTGTGGCCCTGATGGCCCATGGGGGACCACGGGGTCAGCTG

Echidna GCCCCGGTGAGCTGTGCCCTGGTCACCTTCATGGCCCACGGTGGGCGAGGGGGGAGGCTG

Platypus gccctggtaagctgtgccctggtcaccttcagggcccatggcggg**tga**ggggggaggctg

A L V S C A L V T F R A H G G *****

 L  G  A  D  G  Q  E  V  Q  P  E  A  L  M  Q  E  L  S  R  C

Chimpanzee CTGGGGGCTGACGGGCAAGAGGTGCAGCCCGAGGCACTCATGCAGGAGCTGAGCCGCTGC

Echidna CTAGGGGCCGACGGGCAGGAGGTAGAGCCAGAAGATCTGATTGCAGAGCTGCTACCTTGC

Platypus ttgggggccgaagggcaggagatggagccagaggatctgattggagacctgctaccttgc

 Q  V  L  Q  G  R  P  K  I  F  L  L  Q  A  C  R  G

Chimpanzee CAGGTGCTGCAGGGCCGCCCCAAGATCTTCCTGTTGCAGGCCTGCCGTGGGG**GT**GAGCGG

Echidna CGGGCCCTAAGTGGCGGCGTCAAGCTGTTCCTGCTTCAGAGCTGCCGGGGCG**GT**GAGTGT

Platypus tgggctctaagagggggggtcaagctgttcctgcttcagagctgcacggctg**gt**gagtgt

**Supplementary Figure S3. The caspase-16 pseudogene (*CASP16P*) of the platypus contains an in-frame stop codon in the exon encoding the catalytic dyad.** Alignment of nucleotide sequences of the exon encoding the catalytic dyad (corresponding to Histidine 290 and Cysteine 332 of chimpanzee caspase-16, highlighted by yellow shading) of *CASP16* of the chimpanzee (*Pan troglodytes*), echidna (*Tachyglossus aculeatus*) and platypus (*Ornithorhynchus anatinus*). The amino acid sequences translated from these sequences are shown above and below the nucleotide sequences. An in-frame stop codon is highlighted by red fonts. Sequences belonging to the flanking introns are shown with blue fonts. Splice donor sites at the borders of introns are underlined. The nucleotide sequences correspond to GenBank accession numbers NC_072416.2, nucleotides 5816298-5816537 (chimpanzee); NC_052076.1, nucleotides 37244840-37245079 (echidna); NC_041738.1, nucleotides 50894606-50894845 (platypus).

**A**

**Exon 1**

**M** A F

Rat AGGCCCCAAAGTACTGGGAAGCAGAGCCAGGAAGTAAGCTCTGGGCCTGGAGGAAGCCCCTCCTGGTTCTC**ATG**GCCTTC

Mouse AGGCCCCAAGGTACCAGGAAACAGAGCCAGGAAG**TAA**GCCCGGGTCCTGGAGGAAGCCCCACCTGGTTCTCAGAGCCTTC

***** A R V L E E A P P G S Q S L

L V S G T L Q A A A Q L V D A W D S L G R K

Rat TTGGTGTCTGGGACCCTACAAGCCGCTGCACAGCTGGTGGATGCTTGGGACAGCCTGGGGAGAAAG**GT**AGTGAGATGCTC

Mouse CTGGTGTGTGGGCCCCTAAGGGCAGCTGCACAGCTGGCGGATGCTTG----------GGGAGAAAG**GT**AGTGAGATGCTC

P G V W A P K G S C T A G G C L - - - G R K

**B**

**Exon 6**

S D P G P S T Q Y

Rat GAGGACCTGGGAGTCCC------CTACTGAGTGTCCCTAACCCTTTGTAC**AG**TCAGACCCTGGACCCAGCACACAATATG

Mouse GAGGAACTGGGGGTCCCTTCCTTCTACTGTGTGTCCCTATTCCTCTGTAC**AG**TCAGACCCTAGAGCAAGCACACAATATG

S D P R A S T Q Y

D L S G T R A A L L L A V F Q D R L G A R H D V T A L

Rat ACCTGTCTGGGACCAGGGCT-GCCCTCCTACTGGCTGTGTTTCAAGATCGGCTAGGAGCACGGCACGATGTGACAGCACT

Mouse ACCTGTCTGGAACCAGGGCTTGCCCTCCTACTGGCTGTGATTCAAGACCGACTAGGAGCACAGCA**TGA**TGTGGCAGCATT

D L S G T R A C P P T G C D S R P T R S T A *****

R D L C Q A L G F K V T L R T N P S A Q

Rat GAGGGATCTTTGCCAGGCTTTGGGCTTCAAGGTCACCCTGAGGACAAACCCTTCAGCTCAG**GT**TGGGGGAAGCTCAAAGC

Mouse GAGGAACCTTTGCCAGGCCTTGGGCTTCAAGGCCACCCTGAGGATAAACCCATCAGCTCAGATTGGGGGAAGCCCAAAAC

**Supplementary Figure S4. The mouse caspase-16 pseudogene (*CASP16P*) contains frameshift mutations.** Alignment of nucleotide sequences of exon 1 **(A)** and exon 6 **(B)** of *CASP16* of the rat (*Rattus norvegicus*) and *CASP16P* (pseudogene) of the mouse (*Mus musculus*). The amino acid sequences translated from these sequences are shown above and below the nucleotide sequences. Sequences belonging to the flanking introns are shown with blue fonts. Mouse *CASP16P* nucleotide positions differing from the homologous site of the rat are indicated by grey shading. Frameshift mutations are shaded red. The start codon of rat *CASP16* is highlighted by green fonts. The homologous site in the exon 1 of mouse *CASP16P* lacks an in-frame start codon. The amino acid sequence below mouse *CASP16P* is the conceptual translation of codons that fit to the reading frame of exon 2 after splicing. However, a 10-nucleotide deletion shifts the reading frame relative to rat *CASP16* and a stop codon (red fonts) is present on the 5’-end of mouse *CASP16P* **(A)**. A 1-nucleotide deletion causes a frameshift and a premature stop (red fonts) in exon 6 **(B)**. Note that downstream of this site, there is no start codon in frame with caspase-coding sequence. Splice donor sites at the borders of introns are underlined. The nucleotide sequences correspond to GenBank accession numbers **(A)** NC_086028.1, nucleotides 13096060-13095901 (rat) and NC_000083.7, nucleotides 23774749-23774600 (mouse) and **(B)** NC_086028.1, nucleotides 13094234-13094002 (rat) and NC_000083.7, nucleotides 23773043-23772804 (mouse).

**A**

>Pt_CASP1

MADKVLKEKRKLFIRSMGEGTINGLLDELLQTRVLNQEEMEKVKRENATAMDKTRALIDSVIPKGAQACQICITYICEEDNYLAGTLGLSADQTSGNYLNMQDSQGVLSSFPAPQAVQDNPAMPTSSGSEGNVKLCSLEEAQRIWKEKSAEIYPIMDKSSRTRLALIICNEEFDTIPRRTGAEVDITGMTMLLQNLGYSVDVKKNLTASDMTTELKAFAHRPEHKTSDSTFLVFMSHGIREGICGKKYSEQVPDVLQLNAIFNMLNTKNCPSLKDKPKVIIIQACRGDSPGVVWFKDSVGVSGNLSLPTTEEFEDDAIKKAHIEKDFIAFCSSTPDNVSWRHPTMGSVFIGRLIEHMQEYACSCDVEEIFRKVRFSFEQPDGRAQMPTTERVTLTRCFYLFPGH

>Pt_CASP2

MAAPSAGSWSTFQHKELMAADRGRRILGVCGMHPHHQETLKKNRVVLAKQLLLSELLEHLLEKDIITLEMRELIQAKVGSFSQNVELLNLLPKRGPQAFDAFCEALRETKQGHLEDMLLTTLSGLQHVLPPLSCDYDLSLPFPVCESCPLYKKLRLSTDTVEHSLDNKDGPVCLQVKPCTPEFYQTHFQLAYRLQSRPRGLALVLSNVHFTGEKELEFRSGGDVDHSTLVTLFKLLGYDVHVLCDQTAQEMQEKLQNFAQLPAHRVTDSCIVALLSHGVEGAIYGVDGKLLQLQEVFQLFDNANCPSLQNKPKMFFIQACRGDETDRGVDQQDGKNHAGSPGCEESDAGKEKLPKMRLPTRSDMICGYACLKGTAAMRNTKRGSWYIEALAQVFSERACDMHVADMLVKVNALIKDREGYAPGTEFHRCKEMSEYCSTLCRHLYLFPGHPPT

>Pt_CASP3

MAAIVRRLWKLIKVSMENTENSVDSKSIKNLEPKIIHGSQSMDSGISLDNSYKMDYPEMGLCIIINNKNFHKSTGMTSRSGTDVDAANLRETFRNLKYEVRNKNDLTREEIVELMRDVSKEDHSKRSSFVCVLLSHGEEGIIFGTNGPVDLKKITNFFRGDRCRSLTGKPKLFIIQACRGTELDCGIETDSGVDDDMACHKIPVEADFLYAYSTAPGYYSWRNSKDGSWFIQSLCAMLKQYADKLEFMHILTRVNRKVATEFESFSFDATFHAKKQIPCIVSMLTKELYFYH

>Pt_CASP4

MEKERETRNLAEGNHRKKPLKMLESLGKDFLTGVLDNLVEQNVLNWKEEEKKKYYDAKTEDKVRVMADSIQEKQRMAGQMLLQTFFNIDQISPNKKAHPNMEAGPPESGESTDALKLCPHEEFLRLCKERAEEIYPIKERNNRTRLALIICNTEFDHLPPRNGADFDITGMKELLEGLDYSVDVEENLTARDMESALRAFATRPEHKSSDSTFLVLMSHGILEGICGTVHDEKKPDVLLYDTIFQIFNNRNCLSLKDKPKVIIVQACRGANHGELWVRDSPASLEVASSQSSENLEEDAVYKTHVEKDFIAFCSSTPHNVSWRDSTMGSIFITQLITCFQKYSWCCHLEEVFRKVQQSFETPRAKAQMPTIERLSMTRYFYLFPGN

>Pt_CASP5

MAEDSGKKKRRKNFEAMFKGILQSGLDNLVINHMLKNNVAGQTSIQTLVPNTDQKSTSVKKDNHKKKNTVKMLEYLGKDVLHGVFNYLAKHDVLTLKEEEKKKYYDAKIEDKALILVDSLRKNRVAHQMFTQTLLNMDQKITSVKPLLQIEAGPPESAESTNILKLCPREEFLRLCKKNHDEIYPIKKREDRRRLALIICNTKFDHLPARNGAHFDILGMKRLLQGLGYTVIDEKNLTARDMESALRAFAARPEHKSSDSTFLVLMSHGILEGICGTAHKKKKPDVLLYDTIFQIFNNRNCLSLKDKPKVIIVQACRGEKLGELWVRDSPASLAVISSQSSENLEADSVCKIHEEKDFIAFCSSTPHNVSWRDCTRGSIFITELITCFQKYSCCCHLMEIFRKVQKSFEVPQAKAQMPTIERATLTRDFYLFPGN

>Pt_CASP6

MSSASGLRRGHPAGGEENMTETDAFYKREMFDPAEKYKMDHRRRGIALIFNHERFFWHLTLPERRGTCADRDNLTRRFSDLGFEVKCFNDLKAEELLLKIHEASTVSHADADCFVCVFLSHGEGNHIYAYDAKIEIQTLTGLFKGDKCHSLVGKPKIFIIQACRGNQHDVPVIPLDVVDNQTEKLDTNITEVDAASVYTLPAGADFLMCYSVAEGYYSHRETVNGSWYIQDLCEMLGKYGSSLEFTELLTLVNRKVSQRRVDFCKDPSAIGKKQVPCFASMLTKKLHFFPKSN

>Pt_CASP7

MDCVGWPPGRKWHLEKNTSCGGSSGICASYVTQMADDQGCIEEQGVEDSANEDSVDAKPDRSSFVPSLFSKKKKNVTMRSIKTTRDRVRTYQYNMNFEKLGKCIIINNKNFDKVTGMGVRNGTDKDAEALFKCFRSLGFDVIVYNDCSCAKMQDLLKKASEEDHTNAACFACILLSHGEENVIYGKDGVTPIKDLTAHFRGDRCKTLLEKPKLFFIQACRGTELDDGIQADSGPINDTDANPRYKIPVEADFLFAYSTVPGYYSWRSPGRGSWFVQALCSILEEHGKDLEIMQILTRVNDRVARHFESQSDDPRFHEKKQIPCVVSMLTKELYFSQ

>Pt_CASP8

MEGGRRARVVIESKRNFFLGAFPTPFPAEHVELGRLGDSETAMVPGKGGADYILLPFKKMDFSRNLYDIGEQLDSEDLASLKFLSLDYIPQRKQEPIKDALMLFQRLQEKRMLEESNLSFLKELLFRINRLDLLITYLNTRKEEMERELQTPGRAQISAYRVMLYQISEEVSRSELRSFKFLLQEEISKCKLDDDMNLLDIFIEMEKRVILGEGKLDILKRVCAQINRSLLKIINDYEEFSKGEELCGVMTISDSPREQDSESQTLDKVYQMKSKPRGYCLIINNHNFAKAREKVPKLHSIRDRNGTHLDAGALTTTFEELHFEIKPHDDCTVQQIYEILKIYQLMDHSNMDCFICCILSHGDKGIIYGTDGQEAPIYELTSQFTGLKCPSLAGKPKVFFIQACQGDNYQKGIPVETDSEEQPYLEMDLSSPQTRYIPDEADFLLGMATVNNCVSYRNPAEGTWYIQSLCQSLRERCPRGDDILTILTEVNYEVSNKDDKKNMGKQMPQPTFTLRKKLVFPSD

>Pt_CASP9

MDEADRRLLRRCRLRLVEELQVDQLWDALLSRELFRPHMIEDIQRAGSGSRRDQARQLIIDLETRGSQALPLFISCLEDTGQDMLASFLRTNRQAAKLSKPTLENLTPVVLRPEIRKPEVLRPETPRPVDIGSGGFGDVGALESLRGNADLAYILSMEPCGHCLIINNVNFCRESGLRTRTGSNIDCEKLRRRFSSLHFMVEVKGDLTAKEMVLALLELARQDHGALDCCVVVILSHGCQASHLQFPGAVYGTDGCPVSVEKIVNIFNGTSCPSLGGKPKLFFIQACGGEQKDHGFEVASASPEDESPGSNPEPDATPFQEGLRTFDQLDAISSLPTPSDIFVSYSTFPGFVSWRDPKSGSWYVETLDDIFEQWAHSEDLQSLLLRVANAVSVKGIYKQMPGCFNFLRKKLFFKTS

>Pt_CASP10 XP_016805007.2

MKSQGQHWCSSSDKNCKVSFREKLLIIDSNLGVQDVENLKFLCIGLVPNKKLEKSSSASDVFEHLLAEDLLSEDDPFFLAELLYIIRQKKLLQHLNYTKEEVERLLPTRQRVSLFRNLLYELSEGIDSENLKDMIFLLKDSLPKTEMTSLSFLAFLEKQGKIDEDNLTCLEDLCTTVVPKLLRNIEKYKREKAVQIVTPPVDKEAESYQGEEELVSQTDVKTFLEALPQESWQNKHAGSNGNRATNGAPSLVSRGMQGASANTLNSETSTKRATVYRMNRNHRGLCVIVNNHSFTSLKDRQGTHKDAEILSHVFQWLGFTVHIHNNVTKVEMEEVLQRQKCNPAHADGDCFVFCILTHGRFGAVYSSDEALIPIREIMSHFTALQCPRLAEKPKLFFIQACQGEEIQPSVSIEADALNPEKAPTSLQDSIPAEADFLLGLATVPGYVSFRHVEEGSWYIQSLCNHLKKLVPRHEDILSILTAVNDDVSRRVDKQGTKKQMPQPAFTLRKKLVFPVPLDALSL

>Pt_CASP12

MADEKPSNGVLVHMVKLLIKTFLDGIFDDLMENNVLNTDEIHLIGKCLKFVVSNAENLVDDITETAQIAGKIFREHLWNSKKQLSSDISSDGERGANMPGLNICNKEFNYLHNRNGSELDLLGMRDLLENLGYSVVIKENLTAQEMETALRQFAAHPEHQSSDSTFLVFMSHGILNGICGTKHWDQEPDVLHDDTIFEIFNNRNCQSLKDKPKVVIMQACRGNIMSLPDGAGIVWFTTDSGKASADTHGRLLQGNICNDAVTKAHVEKDFIAFKSSTPHNVSWRHETNGSVFISQIIYYFKEYSWSHHLEEIFRKVQHSFETLNILTQLPTIERLSMTRYFYLFPGN

>Pt_CASP14

MSNPRSLEEEKYDMSGARLALTLCVTKAREGSEEDLDALEHMFRQLRFESTMQRDPTAQQFQEELEKFQQAIDSREDPVSCAFVVLMAHGREGFLKGEDGEMVKLENLFEALNNKNCQALRAKPKVYIIQACRGEQRDPGETVGGDEIMMVIKDSPQTIPTYTDALHVYSTVEGYIAYRHDQKGSCFIQTLVDVFTKRKGHILELLTEVTRRMAEAELVQEGKARKTNPEIQSTLRKRLYLQ

>Pt_CASP16

MAFLVAGTLRLAMQMADTRDSLGRKGKYDVQGPRAALMLSSPGVAAAVVTALEDVFQALGFESCARREVPVQGFLEELAWFQEQLDAHGRPVGCALVALVAPRGQLRQPQQLVRELSGCRALRGCPKVFLLLSSGPGSSLEPGAFLAGLRELCGRSPHWSLVQLLTELFRRMAEESAGGTCCPVLRSSLRGALCLGGVEPWRPEPAPGPSTQYDLSKARAALLLAVIQGRPGAQHDVEALGGLCRALGFETTVRTDPTAQAFQEQLAQFREQLDTCRGPVSCALVALMAHGGPRGQLLGADGQEVQPEALMQELSRCQVLQGRPKIFLLQACRGGNRDAGVGPTALPWYWSWLRAPPSVPSHADVLQIYAEAQGSSCRGTPPGSSDQADILTVYSAAEGYVAYRDDKGSDFIQTLVEVLRASPGRDLLELLTEVNRRMCEQEVLGPDCDELRKACLEIRSSLRRRLCLQA

>Bt_CASP1

MADKLLKEKRKLFVHSVSKGTINGLLDELLEKRVLNQEEMEKIRDENDTAMDRARVLIDTVIRKGPQACQICISHICEEDSHLAGILGLTSGSQSENYLRQKPQAVVPPFPAPQAMLDNPVKLASSGPGGNLKLCPPETAQRIRKEKSGEIYPIMERSNRTRLALIICNTEFENLTRRDGADVDTRNMKVLLEGLGYKVDVKENLTASEMILELKAFAAHSEHRTSDSTFLVFMSHGIRAGICGKKYSEEVPDILKVDDIFHILNTGNCPALKDKPKVIIIQACRGEKQGMVWVNDSVAASGNCSLVAPEDFESDAIKKAHIEKDFIAFCSSTPDNVSWRHPIFGSLFIIKLIETFQEYAWSCDLEEIFRKVRFSFELPDGKVQMPTAERVTLTRCFYLFPGY

>Bt_CASP2

MAAPSAGFQYPLQPKEQMAADRGRRMLRGCGMHPDHQEALKKNRVVLAKELLLSELLEHLLEKDIITLEMREHIQAKTGSFGQNVELLNLLPKRGPQAFDAFCVALRETKQSHLEELLLRTLSGLQPVVPPLSCDYDLSLPFPVCESCTRHKRLHLSPDAVEHSLDHGDGPPCLQVKPCTPEFYQTHHHLAYRLQSRPRGLALVLSNVHFTGEKDLEFRSGGDVDHSTLVTLFKLLGYKVHVLLDQTAQEMQEKLQNFAQLPVHRVTDSCIVALLSHGVEGGVYGVDGKLLQLQEVFRLFDNANCPSLQNKPKMFFIQACRGDETDRGVDLQDGKNHDRSPKCEESDASREELLKMRLPTRSDMICGYACLRGTAAMRNTKRGSWYVEALTQVFSERACDMHVADMLVKVNALIKEREGYAPGTEFHRCKEMSEYCSTLCRHLYLFPGHPPT

>Bt_CASP3

MSMENTENSVDSKSIKTSETKILHGSKSMDSGISLEESYKMDYPEMGLCIIINNKNFHENTGMACRSGTDVDAANLRETFMNLKYEVRIKNDLTCKEMLELMSNVSKEDHSKRSSFICVLLSHGEEGIIFGTNGPVNLKKLASFFRGDYCRSLTGKPKLFIIQACRGTELDCGIETDSGAEDDMACQKIPVEADFLYAYSTAPGYFSWRNAKNGSWFIQVLCEMLKKYAHRLELMHILTRVNRKVAIEYESFSTDSAFHAKKQIPCIMSMLTKELYF

>Bt_CASP4

MNKHFFPKGWEIHLLTTGDFPTVNPEVPNKNFSICGYKSQPPVLRKCPVTFIFETVLLQGSRKILSLLSMADKHNKNPLKMLESLGKELISGLLDDFVEKNVLKLEEEEKKKIYDAKLQDKARVLVDSIRQKNQEAGQVFVQTFLNIDKNSTSIKAPEETVAGPDESVGSAATLKLCPHEEFLKLCKERAGEIYPIKERKDRTRLALIICNTEFDHMPPRNGAALDILGMKQLLEGLGYTVEVEEKLTARDMESVLWKFAAREEHKSSDSTFLVFMSHGILDGICGTMHSEEEPDVLPYDTIFRTFNNRNCLSLKDKPKVIIVQACRGANRGELWVSDSPPALADSFSQSSENLEEDAVYKTHVEKDFIAFCSSTPHNVSWRDIKKGSLFITRLITCFQKYAWCCHLEEVFRKVQQSFEKPNVKAQMPTVERLSMTRYFYLFPGN

>Bt_CASP6

MSSEPPPRRARGPGEEQNMTEIDAFPRREIFDPTEKYKMDHKRRGIALIFNHERFFWHLTLPNRPGTSADRDNLRRRFSDLGFEVKCFDDLRAEELLLKIHEASTASHVDADCFLCVFLSHGEGNHIYAYDAKIEIQTLTGLFKGDKCQSLVGKPKIFIIQACRGSQHDVPVIPLDVVDHRTDTPDANLTQVDAASVYTLPAGADFLMCYSVAEGYYSHRETVNGSWYIQDLCEMLGKFGSSLEFTELLTLVNRKVSQRRVDFCRDPNAIGKKQVPCFASMLTKKLHFSPKSK

>Bt_CASP7

MADDQGHIVEQGAEDPANNDTVDAKPDRSSFVSSIFSKKKKNDSGKSVNIPRDRVPTYQYNMNFEKVGKCIIINNKNFDRITGMGVRNGTDKDAEALFKCFRSLGFDVSVYNDCSCAKMQDLLKKASEEDHRNSACFACILLSHGEENLIYGTDGKTAIKDLTAHFRGDRCKTLLEKPKLFFIQACRGTELDDGIQADSGPINDTDANPRYKIPVEADFLFAYSTVPGYYSWRNPGSGSWFVQALCSILNEHGKSLEILQILTRVNDRVARHFESQSDDPRFHEKKQIPCVVSMLTKELYF

>Bt_CASP8

MALTQCLYNIGEQLGSDDLAALKFLSRDHIPYRKQEPIKDALMLFQRLQEKRMLEENNLSFLKELLFRVNRLDLLLNYLYTSEEEMKRELQIPGRAQISAYRILLFQISEDVNKVELKDFKFFLSQEIAKCKLDDDMTLLDIFVEMEKRTILGEENLDTLKRICEQVNKSLLKKIYDYEELRKERRMSLERDPYAFSNDMSQSLPEEGYSKMPAMSDSPEQDSELQTSDTVYRMTSKPRGYCLIFNNYDFSIARKQVPELHDLKDRTGTDFDADALDKTFRELHFEIVHYKDLTAKGICEVLESYQKKDHKNKDCFICCILTHGNKGIIYGSDGQEASIYELTSYFTGLKCPSLIGKPKIFFIQACQGDKYQKGVAVETDSEQMEAYLEVDSSPQKRYIPDEADFLLGMATVKNYVSYRNIWNGAWYIQSLCQNLRERCPRGEDILTILTKVNFEVSKLDDKQRMAKQMPQPTFTLRKKLFFPLN

>Bt_CASP9

MNEADRLLLRRYRVRLVGELQVASLWDALLSRELFTPDMIEDIQRAGSGSRRDQARQLILDLETRGSQALPLFISCLEDTGQDTLASLLKTSRQAAKQDVEAIRPLDLKPVVLGPEGLKPGEPRVMKQDPSKPSQGKLAPVVLGPEELWPAKLRPEVLRPEVPRAVDAGSGGFTDVCPQDRAKGNADLAYVLNADPCGHCLIINNVNFCRESGLRARTGSNIDCERMRRRFHLLQFVVEVKCDLTAKQMVQALMQLARQDHSALDCCMVVILSHGCQASHLQFPGAVYGTDGCPVSVERIVNTFNGTGCPSLRGKPKLFFIQACGGEQKDHGFEVESTSPEDKTPSSDPEADATPFQEGPRSIDEPDAVSSLPTPSDILVSYSTFPGFVSWRDPKSGSWYIETLDSIFEQWAHCEDLQTLLLRVANAVSVKGIYKQMPGCFNFLRKKLFFKT

>Bt_CASP14

MSSPQPLEEETYDMSGARLALTLCVTKAREGSEADLDALERMFQQLGFESTMKRDPTAQQFQEELEKFQQAIDAREDFVSCAFVVLMAHGLEGRLKGKDEKMVELEDLFQALNNKNCRALRAKPKVYIVQACRGEQRDPGEPVTGGHLVMITENTPETIPTYTDTLHVFSTIEGYIAYRHDQEGSYFIQTLVDVFINKKGPILELLTEVTRRMAEAEMVQEGEAKKVNPEIQSTLRKQLYLQ

>Bt_CASP15

MEGSLGSGRRSGSDGQPAPPKISPTTEELQSRLQETLDLLSSQELRSFRDHLKKVEPPVSQVKLELEGHSPSGLAKLLAKHYYPAAVAKRVLVQVLELLPRADLLPRWQSAPTDCPVIPRKSLKRSYDCVDGELYRYDLSGRRKAFLMCVKKNRLGAHQDVQLMKDWLKECKFEPTLCIDPDKMDLLGKITSFRDELNEIKDDIGCCLVTLMSHGEEGFIKMKDGEKVSLEDIFEMFNNKNCPALQEKPKIFIIQACRGERRDSGVETDDEPMDLDDGSEKKRLPTFSDYFIVYPTQADHVALRDPRTGSVMIKEMTEVFKQYGNKWHLADFFTIVNNRVVHRDFNLCNKPVKVSLVMESTLTKFVYF

>Bt_CASP16

MAFLVAGTLQVASQVADAQESLGRKGKYSVKDPRVALALCSPEVSASTAALLEGVFQTLGFESCRRQGASVQGFRGELTRFREQLDAHGGSVGCAFVALVARPWQLRQSQQLVRELSRCKALWGRPKVFLLLSSAPGAVPEPGAFLASLGELCGRRPHWSLLQLLTEVFSRTAEESAGAAYCPVLRSSLRGALCLGNVEPWGPEPEPGPSAQYDLSGARAALLLAVTQGRLGAQHDVEALEGLCQALGFETTLRTDPTAQTFQEEMAQFRKRLDAHRGPVSCALVALMAHGGPQGQLLGADGQERQLEVLVQELSHCGALRGRPKIFLLQACRGGHRDAGVGPAALPWFRRWLRAPPATPSQADVLHVCTDVQGRSSRGPTPRSPNQADVLMVYAAAEGCVAYRDKKGSDFIQTLVEVLRADPKADLLELMTEVNRQVCELDVLGPDCDDRRKACLEIRSSLRRRLCLQV

>Md_CASP1L1

MLFLMYLGAERVSLLFLTDKVLKEKRRLFVESVDRGVINGLLDDLLELQVLNLEEMEILKEESRTIRDKARVLIDAIISKGPKASQCFTKSIWERDGHLAEKLGLSSAPQEIQDDAIDTLKLCSYADFQRIKKEKEGQIYPVMEKGFRTRLALIICNKEFDELPRRNGAENDVTGMQNLLEGLGYRVHVKENLTALEMESALKQFASYPEHRTSDSTFLVLMSHGVPGGICGKDYREKDPQILDVNKIFQIFNTSNCPNLKDKPKILILQACRGGNDGAVLVNDSSDSSEHRCIQNLPIFEDDAVRRAHVEKDFIAFCSSTPDNVSWRNIYKGSLFIIQLILYIKKFAWCYHLYEIFCKVQHSFENREEKLQMPTIERATLTKYFYLFPGN

>Md_CASP1L2

MADKVLMEKRRLFVESVDRGVMNGLLDDLLEVQVLNLEEMEIVREESKTIRDKARVLIDAVISKGPKASQCFTKSICERDGHLAEKLGLSSVPQAIQDDAIDTLKLCSYEDFQQKRKEMEGQIYPVMEKGFRTRLALIICNKEFDELPRRNGAENDVTGMQNLLEGLGYRVHVKENLTALEMESALKQFASYPEHQTSDSTFLVLMSHGVPGGICGKDYTDKVPQILDVNKIFQIFNTSNCPTLKDKPKIIILQACRGENDGVVWVNDSSKSSQQRCSQNLPIFEDDAVRRAHVEKDFIAFCSSTPDNISWRNIYKGSLFIIQLILYIKKFAWCYHLYEIFCKVQHSFETPAEKLQMPTIERATLTKYFYLFPGN

>Md_CASP1L3

MSEAMAVQQLKEKKNMFVESVNKDIINGLLDDLLESGMITQEKMEELRDENVTTMDKALALIEYVIWKGPEACQLLIKSIWHRDPHLARKMNLPYSNQDLKEKRKLFVQFVNKGTINCLLDDLLQANVFNQEEFEQIQKENDTSMDKARVLIDYVIRKGPKASEILIKSIWNRDPHLGHNLKLPYSASTAQTGSSGKLKVCSREEFLMQRRLRVEEIYPVMEKGSRTRLALIICNKKFENISERHGAEIDTIGMKTLLEDLDYNVYVKENLTSLEMESALKEFASHPDHQLSDSTFVVLMSHGVRHGICGIKHKNEDPDVFLYETMYEILNTRNCPNLKDKPKIIIVQACRGDNYGEVWVNDSSASCADSPKELQVFDHDAVSRAHSEKDFIAFHSSTPNNVSWRHPRNGSVFITKLIHYFQQYCWCYHIEEIFRKVQNAFETPEYKFQMPTIERLSMTRYFYLFPGN

>Md_CASP4

MVSFIICDFCFLLLEEVFSYFHYQLCLNCFQTWSQGKQEKQTSGAMADGDSNKKQIKRLEKKLKTKLNISKFTERNKVKLKEGEKKLRPRLKEKLESWEKSLIQKTIAENLLTGGELDVKDVKPHPSDAATYQVLPDEPINTLKICPPEHFKKFMKENEEKIYPVKEKEGRKRLALIICNIEFTYYSERTGAEVDIKGMEGLLKDLGYTVDVQRDLTSSEMASELRKFAARQEHESSDSTFVVLMSHGILDAICGKNHKKEEPDVLPYDTIFNILNTKNCPKLKDKPKVIIIQACRGVKSGKVLMRDSPEISEDSEINPELLEEDAIFETHVEKDFISFFSSTPNNVSWRDHENGSIFIIKVIDYIQKYSWCCHLVDIFQKVQRAFDTANVKLQMPTIERMTMTKNFYLFPGN

>Md_CASP12

MTEQKQSYYPVKIVKGLISTFVDGVIDDLIEKDVLSPNELKHLEKEFSTILNQSEGLVDALDHIFRGSQIIMKKLLLPYLPGNSGSQREDNELAVFMKSKEMHPILSALETICNSSGSVQSYRQRLGNSIFQFFKKIDSNAIQAAVPPNQVKLCRPDFYQGLKEAKEGDIYPIMEKGTRTRLALIICNILFDYLDERQGAYLDIWGMWKLLENLGYTVIIETNLTAQKMESVLKEFADRPEHLSSDSTFLVFMSHGLLNGICGIEHTKQKPDLLATDVIFQIFNDSNCPSLKGKPKVIIIQACRGEKLGITYVMDTPESSAGTPDQPMQDHSGNNPLEQKLLEKDFISFCSTTPHNVSWRVDILGSVFINELIYCFQQYAWCCHLEEIFRKVQKSFEIPKILVQMPTIERQSISKYFYLFPGI

>Md_CASP2

MAEARAGARPAFSRGVSTVGDGGRRMLGVWGMEQEHQEALKKNRVVLAKQLLLSELLEHLLEKDIITLEMRELIQAKVGSFSQNVEFLNLLPKRGPHAFEAFCDALRETKQGHLEDLLHRTLYSFQNLRPPLGCDYDSSFPLPVCDSCPPHKQLRLSVVLSLNLDFENCLPVSASETMEHSLDNGDGPPSLQVKPCTPEFYQTHQHLAYRLQSRPRGLAMVLSNVHFNGEKDLEFRSGGDVDHSSLVTLFKLLDYDVHVLRDQTAQKMQENLQRFAQLSAHQSTDSCMVALLSHGIEGGIYGVDGKLLQLQEVFRLFDNANCPNLQNKPKMFFIQACRGDETDRGVDLRDGKEHVASPGCEESDAGKEKILKMRLPTRSDMICGYACLKGTAAMRNTKRGSWYIEALTQVFSERARDMHVADMLVKVNALIKDREGYAPGTEFHRSKEMSEYSSTLCRHLYLFPGHPPTQ

>Md_CASP3

MEDTGTTVDAKSTKNSGVKLFHGSKSVESGLSSDSYKMDYPEMGLCIIINNKNFHPNTGMSFRSGTDVDAASLSDTFRSLKYEVRIKNDLTCNEITELLNSVSKEDHSQRSSFICVILSHGEEGVIFGTDRSVELKRLTCFFRGDKCRSLTGKPKLFIIQACRGTELDCGVETDSGTDEDIACQKIPVEADFLYAYSTAPGYYSWRNSKDGSWFIQALCAVLKQHAHKLEIMQILTRVNRKVATEFESYSLDISFHAKKQVPCIMSMLTKELYFSH

>Md_CASP6

MCFHLKSFGLFFVFEGDAEQNATEIDAFFKSKPLDPAEQYKMDHKRRGVALIFNHERFFWHLTLPERRGTRADRDNLRQRLSDLGFEVNCFDDLKAEDLLMTIHMVSTSSHIDADCFLCVFLTHGEGNHIYAYDAKIDIQKLTAMFKGDKCQTLVGKPKIFVIQACRGDQHDIPVIPLDVVDHLSDKVDVNETEVDAASVYTLPAGADFLMCYSVAEGYYSHRETVHGSWYIQDLCEMLEKYGSSLEFTELLTLVNRKVSQRRVDFCKDPNAIGKKQVPCFASMLTKKLYLFPKPK

>Md_CASP7

MADESPSVKQVAGDAGDAENDDIMDAKPDRSSFLAVLRKKKNVPEKSNQPQPEYKRIVMPTFQYNMDYKKVGKCIIINNKQFDSKTGMGTRNGTDKDAEGLTKCFRSLGFDVIVYNNRSCSDMRNLLKQVSQEDHTESACFACILLSHGEEDLIYGTDGVTPIKDLTGHFRGDKCKSLLGKPKLFFIQACRGTEFDDGIQTDSGPINDTNANPGCKIPVEADFLFAYSTVPGYYSWRSQAKGSWFVQALCSVLNEHGKSLEIMQILTRVNYMVAMDFESQSDNPCFNEKKQIPCMVSMLTKELYF

>Md_CASP8

MNMDFHTFLYGIAEEIDSADLASLKFLCLDQIPLKKQQHIKDALVLFQTLEERGLLEEDNLFFLKELLFRINRIDLLNEPLNTKGPEMERELQVPNKARISPYRILLFNLSEGVGPSELKSIKFMLSSKIPKCKIEDDMTLMDLFIEMEKRGILGEGNLDALKIVCDQIDKSLLKKIQEYEISKERSMERFPVEIQDVEETMSQPQEVYDSAGEKGNVYKMSSRPRGYCLIINNFDFKISRKERPENHYLTDRRGTNKDEEALKNIFKELHFDIQSFQDLTAEGIQQVLKTFKDNNHESKDCFVCCLLSHGNKGTIYGIDGKEVPIRDLTSYFSGSNCPSLAGKPKVFFIQACQGKATQYGISLDTDSEQQRESLEADKSFQSECIPNEADFLLGMATVENYVSYRDSARGTWYIQSLCKNLKEGCLRGNDILTILTEVNSEVSQKTDPKNNGKQMPQPKFTLRKKLVFPIS

>Md_CASP9

MEESQRELFQKNRLRLVCELQVEPLWDLMLDRELFTRDMIEDIQRAGTRRDQARQLVTDLQTRGKRALPIFISCLEATGQWDLAALLSESNTTLTQQPDPIDIKPVEMIRSGGLRSTDMKLPIKPLVPGSYTSKKDVPQGAVVSTQTQGGRDTSMVGQVYTLNSDPCGYCLIINNVDFCASSGLSSRKGSNIDCEKMHNRFQALHFAVEVERNLSAKGMGAALQQLAERDHSALDCCVVVILSHGCQASHIQFPGAIHGTDGASISVERIVNIFSGSRCPSLGGKPKLFFIQACGGDQRDHGFQVSSNIPEDKSPGSDPETDATPFQNNFDQPDAVASLPTPSDILVSYSTFPGFVSWRDPKSGSWYIETLDRVLEEWADREDLLHMLLMVSNAVSARGTYKQIPGCFNFLRKRFFFKTK

>Md_CASP10

MEDNSKIKFNQQLLSINEKLEPEEIEALKFLCSDLISPRNLEDIESGHQLFQKFMDEDLLNEGDYFLVAELLFLIKHHNLLQKIGYTKEKVKKELSSKRKISSYRVMLYNLSEEITTEDFEHIKFIMKEHIPKTLKTFLSLLRHMEKQNLLSENNLEMLVKICKPLSINLIKRIEQYKSEKGESESLSEQPITEEFLSKSQEEEKLNVASAITGTTSDEMEEAKFYRMDHSHRGHCIIFNNFEFQTMKRRRGSCKDADELKSVFEWLGFTVKIHHNKEKREMEDILQQCSTSPEHRESDCFVCCVLTHGESGSVFSSDEEKIAIRELTSYFKAHRCPGLANKPKLFFFQACQGPDIQESMLLEEDAKISPPIQVENPQRYIPVEADFLLGMATVDGCCAIRHTIKGSWYIQALCHRLKCMVPRNEDILTILTEVNNDVSQLTDAEGKKKQMPQPAFTLRKKVVFPVPPLSPP

>Md_CASP14

MSSESEYTSDQDVYNMSDARLALTLCITKNRAGSETDVQALEKMFKALNFKNTVRRNIPAKGFWEELEKFRDDMDQMKDPVSCCFVVLMAHGEEGILQGVDGNTIRLDELFCMLTNKNCRALRGKPKVFIVQACRGDQKDPGEVVKPTSSGGDVLLAKERPPKLPTFSDSLHVYATVEGYIAYRHEEDGSFFIQTLVDVFTNMKGNILDLLTEVTRRMADAELMEEGKPRKVNPEVQSTLRKLLYLQ

>Md_CASP15

MLQPDMESFKAQVVSTLTKLSPEELRRFGIYLRNVGEEPRVSRHDLEMATKPEELADLLLKRYVMTGTPRVLVQVLGQVPRKDLALEWESMSRMNLKRPYEDDSSELDRYNMDRTRKAFVMCVKTGRPGAKQDISRIKNWLEKCKFEYTYCDDPDEEELFEKLTQFRDGINGIKEEVSCCLVTLMAHGGKGHIKTKSNERVNLSDIFEMFNNENCPALQEKPKIFVIQACRGDKRDGGVVQADDEPMELDLSEKKRLPTSSDYYIIYATQDDHVAFRHPRDGSVMIQAIDEVFRQHGKKWHIADFFTQVNNLVVHTDFYINRNPAKVVLVMESTLTKAVYF

>Md_CASP16 XP_016279603.1

MSFLAAGSLQVAVEKAEGRVCLEKKGDYDTSGARVALTLCATEGQAGEDRIIAALEAMYQVMSCKSYLRRVVKAQSFQEEMASFRENLDDRGSSMNCALVALVAHSERPGWLLGPDGKEVQEKELVRELNHCQALWGKAKVFLLLDIHNTDLGSIAFLSTLTDVCRHFPHWHLLEVLTQVIGKVTQEMPPTGHRCPIFQSSLRGALYLGRRRSQGLELSPIPQGVYDTSGAKVALVLCVTRDRPGAKQDLKALKRLFQTLGFKSILKMNPTAQDFRKELTRFRELLDARRTNVSCALVALMAHGEPQGRLLGADGQMVEVEEMVSELSACQVLQGKAKVVLLQGCRGGNRDPGMRPRALPWLGSWLQHWLQRPSTIPSHADILQVYANLQDVSSKGYSPKNPDQVDILRVYSAAEGYVAYRNENGSDFIQTLVQVIIANPDQDLLELLTEVNKRLCEMEVRGPDCDEIRKMSVEIQSSLRKQLYLKSQLSN

>Md_CASP18 NP_001107832.1

MDEILLQIKDKLTRSDLESLKFLSSDIIPLQKQENITRSLAFFQALQKRELLDNNSILEELLFLIGRKDILTKQLNVNIEELKRKLQHSDSLKISPYRKLLFKIAENMTSDYLDSAKFLLRRDLPQSKLEGIKTPLKLFIEMEKHGLIEKTNLKALKDILQSLNAECLIKHIKEYEKKTKDLAGSAVTAQLESHLGHLSLLSGNAGQAAAPLNIPEESEMNTLPQSMPPYKMEHVPHGYVVIIDNIHFSNPVDVRIGTEKDVAALRKVFGRLQFKEEYHSNLDASQLHEVMKDYSKRDYTDQDAFICCILSHGKKGVVLGTDWKPVAIKKLLSYFTANECKTLKDKPKLFFIQACQNGKSDSLPEVDVEFDLEADAICSNTTHEWSDIFIGMATVEDSLAQRSGSIGSPYIQNLCKELEAHCPQKKELLEIMTSVNSKVSNIIQMPEFRSTLRAPFIFQVPEESQKAPIGH

>Ta_CASP1

MATQLLKDRWCLIIESLTHGMISGLLDDLLQTQVINQEEMDTVREENQRPAEKSRALLNSVIPKGDLASQIFIDALCKRNPFIAAKLGLSTDRQGQSSNTQLVAHSPVYPAMAASCSAPQALQAPKALTESHPDGPVEILRLCTSEEREKLQKENAGEIYPVLNKTGRKRQALIICNIKFDELLERVGAELDIKGMKKLLEDLDYNVQIERNLSATEMESTLKLFAQQPEHKFSDSTFLVFMSHGILEGICGTKFKTQDPDVLYYSTIFRIFNNLNCPGLRDKPKIIIVQACRGENEGMALVSDSLGASAYSSQDLEDLENDAIHRTHVEKDLIAFCSSTPDNVSWRDPKTGSLFITQLIKCFQNHAWGCDLESLFMKVQGHFETPKRKLQMPTRERATLTKRFFLFPGY

>Ta_CASP2

MLGMCGMQPCHQEALKKNRVALAKQLMLSELLEHLLEKDVITMEMRELIQAKAGSFSQNVEFLNLLPKRGPKAFDAFCAALRETKQGHLQDLLCRTLSTLSPTSAALGCDYDSSLPFPTRESGLALKKPRLSEEEMENSLDNGDGPPCPQVKPCTPEFYHVHHHLAYRLESRPRGLALVLSNVRFNGEKDLEFRSGGDVDHNALVTLFKHLDYSVEFLXDQTAQEMQEKLHSFAQSPRHRYTDSCIVALLSHGVEGGIYGVDGKLLQLQEVFRLFDNANCPSLQNKPKMFFIQACRGDETDRGVDQLDGKERAASPGCEESDAGKEEMLKVRLPTRSDMICGYACLKGTAAMRNTKRGSWYIEALTQVFAESARDMHVADMLVKVNALIKEREGYAPGTEFHRCKEMSEYCSTLCRHLYLFPGR

>Ta_CASP3

MADSQVEAGAAAGEVMVDSKSFHSSHGKVPGKQFLGSSASLDHRYRMDYPNMGICLIINNKNFHPNTGMGCRSGTDVDAASLIDTFKKLKYEVRCKNDMKRHEILELLTSVAHEDHSKRSSFVCVLLSHGEEGVIFGTDGSLELKSLANLFRGDNCRSLVGKPKLFIVQACRGTELDSGVEADSSSADDGPEQKIPVEADFLYAYSTAPGYYSWRNSLNGSWFIQALCAMLKQHAPTLELLHILTRVNRKVATEFESYSPDASFHAKKQIPCIVSMLTKELYFPC

>Ta_CASP6

MMEIDGFYSRETLDPAAKYKMEYKRRGVALIFNHERFYWQLKLPERRGTRADRENLTRRLSALGFEVKCFDDLKAEEVMLKIHEASSSNHADADCFLCVFLSHGLGNHVYAYDGKIDIPEITSMFKGDQCRSLVGKPKIFIFQACRGEEHDVGVVPLDVLDSKMSEQDVNETEVDAASVYTLPAGADFLMCYSVAEGYYSHRETVNGSWYVQDLCEMLGRYGSSLEFTELLTLVNRKVSYRRVDFCNNPRAIGKKQIPCFASMLTKKLHFLPKAQ

>Ta_CASP7

MGQMADDQTPTMHQEENDSEDEVDAKPERLKVAKLFSRKKDEAESQSGGLLPERNRVVSSSFTYKMNYENLGKCIIINNKNFDSKTGMNTRNGTDKDAGSLQKCFRNLGFDVSVFNDFSCNQMKEILQKASEENHENSACFACILLSHGEGDCIYGTDDCIPIKELTILFRGDKCKSLLGKPKLFFIQSCRGTEFDDGIQTDSTPVTDPDANPGCRIPVEADFLFAYSTVPGYYSWRNVERGSWFVQALCSVLNDHGKELEIMQLLTRVNHQVAINFESWCSDPYYCQKKQIPCLVSMLTKELYF

>Ta_CASP8 XP_038605280.1

MDFHKLLFSIGEELAADDLEALKFLCLDLIPLKKQETIQDALGLFWLLQEKGRLEVGKLSFLKELLFRIHRIDLLTTHLGTSQEDVSRELEEPGRAQVSAYRVLLFTLSDNVTRDELKSITFLLSNELPKSRLDNKTLMGIFIEMEKKGILGENNLDILKEICDKIDKTLLGKIDHYEERRAVQGLSSGFQEMSDARGEQGSQPQKNGDFYKMNSNPRGYCLIINNYNFQQARAEVPKLKKMKNRDGTDKDAEALRQIFKALAFLPVVLEDQTANQILETLQRFQRMDHSAQDCFVCCILSHGNRGVVYGTDGQQASICSLTSYFTGSRCPTLAGKPKLFFIQACQGDAYHLAVALETDTGPAAASPAEPDAVFHERCIPDEADFLLGMATVSNCVSYRVPSTGTWYIQSLCHHLRNLCPLGDDILTILTKVNFEVSQKIDLNNRGKQMPQPMFTLRKKLVLPPL

>Ta_CASP9 XP_038602498.1

MEERGRQALRGARLRLVRELRVAPLWDLLLARGLFTRDMIDHIQQAGTRRDQARQLVTDLETRGRQALPVFISCLRETDQHVLAALLSEGCQALQFAPVDVRPVEIIRPGGQGDVYVKPFSPMPSTTKREQEATRPGLVEFPQDKSRRSSDLVYSLKSEPCGHCLIINNVDFSLGSELSTRTGSDVDCEKIQRRFRLLHFEVAVERNLTAEEMVKALNKLARKDHSALDCCVVVILSHGYQASHIQFPGGIHGTDGKSISVETIVNYFNGSRCPSLGGKPKLFFIQACGGEQKDRGFEVNLDSPEDHPTRRDPESDATPFQAAPEGSDEPDAVASLPTPSDILVSYSTFPGFVSWRDTKSGSWYIETLDSVLEQWAHTEDLLTLLLRVSNGVSSKGKYKQIPGCFNFLRKRLYFNTK

>Ta_CASP10 XP_038605241.1

MNDDRGVGFRQQLLNIDKNLGSEEVEALKFLCNDWIPFKKLEKVRSAQEIFQHLEDGDRLNRENPFVVVELLYHIRQHALLKHVGYTKEKVIKELPAKGKLSPFRKMLFELSEDITQEDLKSMLFLLADHLPKKKMQSTLSLLLFLEKQELLGENNLEKLEEVCRQVSPELVRRINKYKNERHFRKADPVSLQEHDDKVAKVPLPVSRELVGESNFRRGSEGSTEEMSTYRMDRARRGYCIIFNNFTFKGKLNSRKGTQKDVAELQRVFRWLGLDVETFNDKTRQEMVATLEECSRRPDHDVRDCLVCCVLSHGESGAVYSADEELIPIRQIMSYFTAKGCPGLAHKPKLFFIQACQGKDIQEAVQIEPDARNPELDPQPEPPPAPRESPKDSIPAEVDFLLGMATVDGYASFRHVYQGTWYIQALCRQLQLLVPRREDILSILTAVNDDVSQRADNLGKKKQMPQPAFTLRRKLVFPVPRGAPPSSLE

>Ta_CASP14L1 XP_038598963.1

MSSEEMVYDMSGARLALTLCITKGREGAEPDIAALERMYRALRFESTVKRDPPAQGFRDEVVHFREEMEKRTDPISCCFVVLMAHGKEGRLLGADGQVVELEELYDVLTNKTCRALLGKSKVFILQACRGDQKDTGEMLRTEQVLTTMFQELPKIPTFTDTLHVYPTVEGYIAYRNVKKGSCFIQTLVDVFITGKSNILDLLTEVTRRMTEAELVWEGQVRKVNPEIYSTLRKQLYLQ

>Ta_CASP14L2 XP_038598364.1

MSSEEMAYDMSEARLALTLCITKGREGAEPDMVALERMYRALRFESTVKRDPPAQGFRDEVVHFREEMEKRTDPISCCFVVLMAHGKEGRLLGADGQVVELEELYDVLTNKTCRALLGKPKVFILQACRGDQKDTGEMLRTEQVLTTMFQELPKIPTFTDTLHVYSTVVGYIAYRNIKEGSCFIQTLVDVFITGKSNILDLLTEVTRQMVEAELVWEDHVRKVNPEIYSTLRKQLYLQ

>Ta_CASP14L3 XP_038598727.1

MSSEEMVYDMSGARLALTLCITKGREGAEPDIMALERMYRALRFESTVKRDPPAQGFWDELVHFREEMEKRTDPISRYFVVLMAHGKEGRLLGADGQVVELEELYDILTNKTYRALLGKPKVFILQACRGDQKDTGEMLRTEQVLTTMFQELPKIPTFTDTLHVYSTVEGYIAYRNVKEGSCFIQTLLDVFITGKSNILDLLTDVTRQMVEAELVWEGQVRKVNPEIYSTLRKQLYLQ

>Ta_CASP14L4 XP_038599033.1

MSSEEMVYDMSGSWLALTLCITKGREGAEPDIVAQERMYRTLRFESTVKRNPPAQGFRGEVAHFREEMEKRTDPISCCFVVLMAHGKEGRLLGADGQVVELEELYDVLTNKTCRALLGKPKVFILKACLGDQKDTGEMLRTEQVLTTMFQELPKIPTFTDTLHVYSTVEGYIAYWNVKEGSCLIQTLVDVFITGKSNILNLLTEVTRWMAEAKLVWEGQVRKVNPEIYSTLRKQPYLQ

>Ta_CASP15_XP_038617796.1

MAPPLQGAVERLRPQELKRLKFQLSYLPAPDRLPAGLLAQAHDGPALLRLLQFHLGPRAPDALQRALEATPGLRAPVFLPGPYPDPNNLLPPGNKRKRLDQCDQDEPKRYDMSKCREAFLMGVKKGRSGATQDIEKLRKHLAAFRFSVQECIDPDGKEILQDLRAFRDKINGSPYEMSCCLVVLMSHGVDGFIKGKDEEEVNLEEIFKLFNNANCPNLREKPKIFIIQACRGDRRDGGVEAFDNVAEEMDDTSNVKRLPTTSDFFIVYSTQKGHVSVRNPLLGSRMIEAIDDVFSRYGNKWQLTDLFTKVNDELVHRDFYGFQCVVKVTLEMQSTLTRAVYLASEQDL

>Ta_CASP16 XP_038609296.1

MRTRATAQAVGGVIGETAPPGKSCPAFRSSLRGMLCLGRRDVEVTGGDSEPCTSNRGGGPAPQEEYDMSGARVALTLCVFRDRAGAERDVEALERLCRTLGFESSVRRDPTAQDFRDEMAQFRAKLDGRGAPVSCALVTFMAHGGRGGRLLGADGQEVEPEDLIAELLPCRALSGGVKLFLLQSCRGGQRDSGAGAPGFPWLRRWLRGPPAIPSHADILRVYGDVRGRSSMPAPSGLDQADTLRVYAAADGCVAYRDEQGSDFIQTVVEVLLAAPPHRDLLDLLTEVNRKMCEADVLGPNSDDICKMNLEIQSSLRKRLCLQAPLRLITGKGESPRGRTPTQDEL

>Ta_CASP18 XP_038605767.1

MSFQVKELLFSIGEELAADDLEALKFLCLDLIPLKKQETIQDALGLFWLLQEKGKMEEGKLSFLKELLFRIHRIDLLTAHLGTSQEDVEMELEEPGRAQVSAYRYLLYQIAEELTKENLTSIKFLLLSEIPKSKLQDPVTALSIFVHMEKHGKMNEGDLGMLKKILKDVQPSLLKPIEMYEEKSRAGAGAAGNLSTALGQLSLTTGNYNRQVSSPKAAEEMERISQIIAPYKMSHLPHGFCVIINNSEFQNPQNTRRGADKDAAALDKVFSWLQFKVEHHSNLKGEAITRVLRAYSERDHTDHDCFICCLLSHGQKGEILGTDWEPVPLRALLGLFTSSACRTLAAKPKLFFVQACQGGQGQGGLSLRGHDDALLESDAVVIPSIPDWADLLVGMATVEDFICFRRPCHGSEYIQALCRALETFCPRGNDLLTILTHVNKKVGQKVFGNNKQMPEVKFTLQRPLIFPVPELGNELEEQEEGC

**B**

>Pt_CASP12

ISSDGERGANMPGLNICNKEFN----------YLHNRNGSELDLLGMRDLLENLGYSVVIKENLTAQEMETALRQFAAHPEHQSSD--STFLVFMSHGIL-------NGICGTKHWDQEPDVLHDDTIFEIFNNRNCQSLKDKPKVVIMQACRGNIMSLPDGAGI---------VWFTTDSG----------KASADTHGRLLQGNICNDAVTK-AH---VEKDFIAFKSSTPHNVSWRHETNGSVFISQIIYYFKEYSWS-HHLEEIFRKVQHSFETLNI----------LTQLP-TIERLSMTRYFYL

>Md_CASP4

YPVKEKEGRKRLALIICNIEFT----------YYSERTGAEVDIKGMEGLLKDLGYTVDVQRDLTSSEMASELRKFAARQEHESSD--STFVVLMSHGIL-------DAICGKNHKKEEPDVLPYDTIFNILNTKNCPKLKDKPKVIIIQACRG--VK----SGK---------VLM-RDSP----------EISED-SEINPELLE-EDAIFE-TH---VEKDFISFFSSTPNNVSWRDHENGSIFIIKVIDYIQKYSWC-CHLVDIFQKVQRAFDTANV----------KLQMP-TIERMTMTKNFYL

>Ta_CASP1

YPVLNKTGRKRQALIICNIKFD----------ELLERVGAELDIKGMKKLLEDLDYNVQIERNLSATEMESTLKLFAQQPEHKFSD--STFLVFMSHGIL-------EGICGTKFKTQDPDVLYYSTIFRIFNNLNCPGLRDKPKIIIVQACRG--EN----EGM---------ALV-SDSL----------GASAY-SSQDLEDLE-NDAIHR-TH---VEKDLIAFCSSTPDNVSWRDPKTGSLFITQLIKCFQNHAWG-CDLESLFMKVQGHFETPKR----------KLQMP-TRERATLTKRFFL

>Md_CASP12

YPIMEKGTRTRLALIICNILFD----------YLDERQGAYLDIWGMWKLLENLGYTVIIETNLTAQKMESVLKEFADRPEHLSSD--STFLVFMSHGLL-------NGICGIEHTKQKPDLLATDVIFQIFNDSNCPSLKGKPKVIIIQACRGEK------LGI---------TYV-MDTP----------ESSAGTPDQPMQDHSGNNPLEQ-KL---LEKDFISFCSTTPHNVSWRVDILGSVFINELIYCFQQYAWC-CHLEEIFRKVQKSFEIPKI----------LVQMP-TIERQSISKYFYL

>Bt_CASP4

YPIKERKDRTRLALIICNTEFD----------HMPPRNGAALDILGMKQLLEGLGYTVEVEEKLTARDMESVLWKFAAREEHKSSD--STFLVFMSHGIL-------DGICGTMHSEEEPDVLPYDTIFRTFNNRNCLSLKDKPKVIIVQACRGAN------RGE---------LWV-SDSP----------PALADSFSQSSENLE-EDAVYK-TH---VEKDFIAFCSSTPHNVSWRDIKKGSLFITRLITCFQKYAWC-CHLEEVFRKVQQSFEKPNV----------KAQMP-TVERLSMTRYFYL

>Pt_CASP5

YPIKKREDRRRLALIICNTKFD----------HLPARNGAHFDILGMKRLLQGLGYTVIDEKNLTARDMESALRAFAARPEHKSSD--STFLVLMSHGIL-------EGICGTAHKKKKPDVLLYDTIFQIFNNRNCLSLKDKPKVIIVQACRGEK------LGE---------LWV-RDSP----------ASLAVISSQSSENLE-ADSVCK-IH---EEKDFIAFCSSTPHNVSWRDCTRGSIFITELITCFQKYSCC-CHLMEIFRKVQKSFEVPQA----------KAQMP-TIERATLTRDFYL

>Pt_CASP4

YPIKERNNRTRLALIICNTEFD----------HLPPRNGADFDITGMKELLEGLDYSVDVEENLTARDMESALRAFATRPEHKSSD--STFLVLMSHGIL-------EGICGTVHDEKKPDVLLYDTIFQIFNNRNCLSLKDKPKVIIVQACRGAN------HGE---------LWV-RDSP----------ASLEVASSQSSENLE-EDAVYK-TH---VEKDFIAFCSSTPHNVSWRDSTMGSIFITQLITCFQKYSWC-CHLEEVFRKVQQSFETPRA----------KAQMP-TIERLSMTRYFYL

>Pt_CASP1

YPIMDKSSRTRLALIICNEEFD----------TIPRRTGAEVDITGMTMLLQNLGYSVDVKKNLTASDMTTELKAFAHRPEHKTSD--STFLVFMSHGIR-------EGICGKKYSEQVPDVLQLNAIFNMLNTKNCPSLKDKPKVIIIQACRGDS------PGV---------VWF-KDSV----------GVSGNLSLPTTEEFE-DDAIKK-AH---IEKDFIAFCSSTPDNVSWRHPTMGSVFIGRLIEHMQEYACS-CDVEEIFRKVRFSFEQPDG----------RAQMP-TTERVTLTRCFYL

>Bt_CASP1

YPIMERSNRTRLALIICNTEFE----------NLTRRDGADVDTRNMKVLLEGLGYKVDVKENLTASEMILELKAFAAHSEHRTSD--STFLVFMSHGIR-------AGICGKKYSEEVPDILKVDDIFHILNTGNCPALKDKPKVIIIQACRGEK------QGM---------VWV-NDSV----------AASGNCSLVAPEDFE-SDAIKK-AH---IEKDFIAFCSSTPDNVSWRHPIFGSLFIIKLIETFQEYAWS-CDLEEIFRKVRFSFELPDG----------KVQMP-TAERVTLTRCFYL

>Md_CASP1L3

YPVMEKGSRTRLALIICNKKFE----------NISERHGAEIDTIGMKTLLEDLDYNVYVKENLTSLEMESALKEFASHPDHQLSD--STFVVLMSHGVR-------HGICGIKHKNEDPDVFLYETMYEILNTRNCPNLKDKPKIIIVQACRGDN------YGE---------VWV-NDSS----------ASCAD-SPKELQVFD-HDAVSR-AH---SEKDFIAFHSSTPNNVSWRHPRNGSVFITKLIHYFQQYCWC-YHIEEIFRKVQNAFETPEY----------KFQMP-TIERLSMTRYFYL

>Md_CASP1L2

YPVMEKGFRTRLALIICNKEFD----------ELPRRNGAENDVTGMQNLLEGLGYRVHVKENLTALEMESALKQFASYPEHQTSD--STFLVLMSHGVP-------GGICGKDYTDKVPQILDVNKIFQIFNTSNCPTLKDKPKIIILQACRGEN------DGV---------VWV-NDSS----------KSSQQRCSQNLPIFE-DDAVRR-AH---VEKDFIAFCSSTPDNISWRNIYKGSLFIIQLILYIKKFAWC-YHLYEIFCKVQHSFETPAE----------KLQMP-TIERATLTKYFYL

>Md_CASP1L1

YPVMEKGFRTRLALIICNKEFD----------ELPRRNGAENDVTGMQNLLEGLGYRVHVKENLTALEMESALKQFASYPEHRTSD--STFLVLMSHGVP-------GGICGKDYREKDPQILDVNKIFQIFNTSNCPNLKDKPKILILQACRGGN------DGA---------VLV-NDSS----------DSSEHRCIQNLPIFE-DDAVRR-AH---VEKDFIAFCSSTPDNVSWRNIYKGSLFIIQLILYIKKFAWC-YHLYEIFCKVQHSFENREE----------KLQMP-TIERATLTKYFYL

>Ta_CASP15

LDQCDQDEPKRYDMSKCREAFL--------MGVKKGRSGATQDIEKLRKHLAAFRFSVQECIDPDGKEILQDLRAFRDKINGSPYEMSCCLVVLMSHGVD-------GFIKGK-----DEEEVNLEEIFKLFNNANCPNLREKPKIFIIQACRGDRRD----GGV-------------------------------EAFDNVAEEMDDTSNVKR-LP---TTSDFFIVYSTQKGHVSVRNPLLGSRMIEAIDDVFSRYGNK-WQLTDLFTKVNDELVHRDFYGFQCV----VKVTL-EM-QSTLTRAVYL

>Bt_CASP15

YDLSGR--RKAFLMCVK-----------------KNRLGAHQDVQLMKDWLKECKFEPTLCIDPDKMDLLGKITSFRDELNEIKDDIGCCLVTLMSHGEE-------GFIKMK-----DGEKVSLEDIFEMFNNKNCPALQEKPKIFIIQACRGERRD----SGV----------ET-DDEP----------MDLDDGSE-----------KKR-LP---TFSDYFIVYPTQADHVALRDPRTGSVMIKEMTEVFKQYGNK-WHLADFFTIVNNRVVHRDFNLCNKP----VKVSL-VM-ESTLTKFVYF

>Md_CASP15

YNMDRT--RKAFVMCVK-----------------TGRPGAKQDISRIKNWLEKCKFEYTYCDDPDEEELFEKLTQFRDGINGIKEEVSCCLVTLMAHGGK-------GHIKTK-----SNERVNLSDIFEMFNNENCPALQEKPKIFVIQACRGDKRD----GGV---------VQA-DDEP-----------MELDLSE-----------KKR-LP---TSSDYYIIYATQDDHVAFRHPRDGSVMIQAIDEVFRQHGKK-WHIADFFTQVNNLVVHTDFYINRNP----AKVVL-VM-ESTLTKAVYF

>Pt_CASP14

YDMSGA--RLALTLCVT-----------------KAREGSEEDLDALEHMFRQLRFESTMQRDPTAQQFQEELEKFQQAIDSREDPVSCAFVVLMAHGRE-------GFLKGE-----DGEMVKLENLFEALNNKNCQALRAKPKVYIIQACRGEQRD----PGE---------------------------TVGGDEIMMVI-----KDSPQT-IP---TYTDALHVYSTVEGYIAYRHDQKGSCFIQTLVDVFTKRK---GHILELLTEVTRRMAEAELVQEGKA----RKTNP-EI-QSTLRKRLYL

>Bt_CASP14

YDMSGA--RLALTLCVT-----------------KAREGSEADLDALERMFQQLGFESTMKRDPTAQQFQEELEKFQQAIDAREDFVSCAFVVLMAHGLE-------GRLKGK-----DEKMVELEDLFQALNNKNCRALRAKPKVYIVQACRGEQRD----PGE--------------------------------PVTGGHLVMITENTPET-IP---TYTDTLHVFSTIEGYIAYRHDQEGSYFIQTLVDVFINKK---GPILELLTEVTRRMAEAEMVQEGEA----KKVNP-EI-QSTLRKQLYL

>Md_CASP14

YNMSDA--RLALTLCIT-----------------KNRAGSETDVQALEKMFKALNFKNTVRRNIPAKGFWEELEKFRDDMDQMKDPVSCCFVVLMAHGEE-------GILQGV-----DGNTIRLDELFCMLTNKNCRALRGKPKVFIVQACRGDQKD----PGE-----------------------------VVKPTSSGGDVLLAKERPPK-LP---TFSDSLHVYATVEGYIAYRHEEDGSFFIQTLVDVFTNMK---GNILDLLTEVTRRMADAELMEEGKP----RKVNP-EV-QSTLRKLLYL

>Ta_CASP14L4

YDMSGS--WLALTLCIT-----------------KGREGAEPDIVAQERMYRTLRFESTVKRNPPAQGFRGEVAHFREEMEKRTDPISCCFVVLMAHGKE-------GRLLGA-----DGQVVELEELYDVLTNKTCRALLGKPKVFILKACLGDQKD----TGE---------------------------MLRTEQVLTTM-----FQELPK-IP---TFTDTLHVYSTVEGYIAYWNVKEGSCLIQTLVDVFITGK---SNILNLLTEVTRWMAEAKLVWEGQV----RKVNP-EI-YSTLRKQPYL

>Ta_CASP14L3

YDMSGA--RLALTLCIT-----------------KGREGAEPDIMALERMYRALRFESTVKRDPPAQGFWDELVHFREEMEKRTDPISRYFVVLMAHGKE-------GRLLGA-----DGQVVELEELYDILTNKTYRALLGKPKVFILQACRGDQKD----TGE---------------------------MLRTEQVLTTM-----FQELPK-IP---TFTDTLHVYSTVEGYIAYRNVKEGSCFIQTLLDVFITGK---SNILDLLTDVTRQMVEAELVWEGQV----RKVNP-EI-YSTLRKQLYL

>Ta_CASP14L1

YDMSGA--RLALTLCIT-----------------KGREGAEPDIAALERMYRALRFESTVKRDPPAQGFRDEVVHFREEMEKRTDPISCCFVVLMAHGKE-------GRLLGA-----DGQVVELEELYDVLTNKTCRALLGKSKVFILQACRGDQKD----TGE---------------------------MLRTEQVLTTM-----FQELPK-IP---TFTDTLHVYPTVEGYIAYRNVKKGSCFIQTLVDVFITGK---SNILDLLTEVTRRMTEAELVWEGQV----RKVNP-EI-YSTLRKQLYL

>Ta_CASP14L2

YDMSEA--RLALTLCIT-----------------KGREGAEPDMVALERMYRALRFESTVKRDPPAQGFRDEVVHFREEMEKRTDPISCCFVVLMAHGKE-------GRLLGA-----DGQVVELEELYDVLTNKTCRALLGKPKVFILQACRGDQKD----TGE---------------------------MLRTEQVLTTM-----FQELPK-IP---TFTDTLHVYSTVVGYIAYRNIKEGSCFIQTLVDVFITGK---SNILDLLTEVTRQMVEAELVWEDHV----RKVNP-EI-YSTLRKQLYL

>Md_CASP16

YDTSGA--KVALVLCVT-----------------RDRPGAKQDLKALKRLFQTLGFKSILKMNPTAQDFRKELTRFRELLDARRTNVSCALVALMAHGEPQ------GRLLGA-----DGQMVEVEEMVSELSA--CQVLQGKAKVVLLQGCRGDVSS----KGY----------------------------------------------SPK-NP---DQVDILRVYSAAEGYVAYRN-ENGSDFIQTLVQVIIANPDQ--DLLELLTEVNKRLCEMEVRGPDCDEI--RKMSV-EI-QSSLRKQLYL

>Ta_CASP16

YDMSGA--RVALTLCVF-----------------RDRAGAERDVEALERLCRTLGFESSVRRDPTAQDFRDEMAQFRAKLDGRGAPVSCALVTFMAHGGRG------GRLLGA-----DGQEVEPEDLIAEL--LPCRALSGGVKLFLLQSCRGGQRD----SGAGAPGFPWLRRWL-RGPP----------AIPSHADILRVYGDVRGRSSMP-APSGLDQADTLRVYAAADGCVAYRD-EQGSDFIQTVVEVLLAAPPH-RDLLDLLTEVNRKMCEADVLGPNSDDI--CKMNL-EI-QSSLRKRLCL

>Pt_CASP16

YDLSKA--RAALLLAVI-----------------QGRPGAQHDVEALGGLCRALGFETTVRTDPTAQAFQEQLAQFREQLDTCRGPVSCALVALMAHGGPR------GQLLGA-----DGQEVQPEALMQELS--RCQVLQGRPKIFLLQACRGGNRD----AGVGPTALPWYWSWL-RAPP----------SVPSHADVLQIYAEAQGSSCRGTPPGSSDQADILTVYSAAEGYVAYRDDK-GSDFIQTLVEVLRASPGR--DLLELLTEVNRRMCEQEVLGPDCDEL--RKACL-EI-RSSLRRRLCL

>Bt_CASP16

YDLSGA--RAALLLAVT-----------------QGRLGAQHDVEALEGLCQALGFETTLRTDPTAQTFQEEMAQFRKRLDAHRGPVSCALVALMAHGGPQ------GQLLGA-----DGQERQLEVLVQELS--HCGALRGRPKIFLLQACRGGHRD----AGVGPAALPWFRRWL-RAPP------ATPSQADVLHVCTDVQGRSSRGPTPR-SP---NQADVLMVYAAAEGCVAYRD-KKGSDFIQTLVEVLRADPKA--DLLELMTEVNRQVCELDVLGPDCDDR--RKACL-EI-RSSLRRRLCL

>Ta_CASP2

YRLESR--PRGLALVLSNVRFN-------GEKDLEFRSGGDVDHNALVTLFKHLDYSVEFLXDQTAQEMQEKLHSFAQSPRHRYTD--SCIVALLSHGVE-------GGIYGV-----DGKLLQLQEVFRLFDNANCPSLQNKPKMFFIQACRGDETD----RGV----------DQ-LDGK----------ERAASPGCEESDAGKEEMLKVR-LP---TRSDMICGYACLKGTAAMRNTKRGSWYIEALTQVFAESARD-MHVADMLVKVNALIKEREG-YAPGTEFHRCKEMS-EY-CSTLCRHLYL

>Md_CASP2

YRLQSR--PRGLAMVLSNVHFN-------GEKDLEFRSGGDVDHSSLVTLFKLLDYDVHVLRDQTAQKMQENLQRFAQLSAHQSTD--SCMVALLSHGIE-------GGIYGV-----DGKLLQLQEVFRLFDNANCPNLQNKPKMFFIQACRGDETD----RGV----------DL-RDGK----------EHVASPGCEESDAGKEKILKMR-LP---TRSDMICGYACLKGTAAMRNTKRGSWYIEALTQVFSERARD-MHVADMLVKVNALIKDREG-YAPGTEFHRSKEMS-EY-SSTLCRHLYL

>Pt_CASP2

YRLQSR--PRGLALVLSNVHFT-------GEKELEFRSGGDVDHSTLVTLFKLLGYDVHVLCDQTAQEMQEKLQNFAQLPAHRVTD--SCIVALLSHGVE-------GAIYGV-----DGKLLQLQEVFQLFDNANCPSLQNKPKMFFIQACRGDETD----RGV----------DQ-QDGK----------NHAGSPGCEESDAGKEKLPKMR-LP---TRSDMICGYACLKGTAAMRNTKRGSWYIEALAQVFSERACD-MHVADMLVKVNALIKDREG-YAPGTEFHRCKEMS-EY-CSTLCRHLYL

>Bt_CASP2

YRLQSR--PRGLALVLSNVHFT-------GEKDLEFRSGGDVDHSTLVTLFKLLGYKVHVLLDQTAQEMQEKLQNFAQLPVHRVTD--SCIVALLSHGVE-------GGVYGV-----DGKLLQLQEVFRLFDNANCPSLQNKPKMFFIQACRGDETD----RGV----------DL-QDGK----------NHDRSPKCEESDASREELLKMR-LP---TRSDMICGYACLRGTAAMRNTKRGSWYVEALTQVFSERACD-MHVADMLVKVNALIKEREG-YAPGTEFHRCKEMS-EY-CSTLCRHLYL

>Pt_CASP9

YILSME--PCGHCLIINNVNFC-------RESGLRTRTGSNIDCEKLRRRFSSLHFMVEVKGDLTAKEMVLALLELARQ-DHGALD--CCVVVILSHGCQASHLQFPGAVYGT-----DGCPVSVEKIVNIFNGTSCPSLGGKPKLFFIQACGGEQKD----HGF----------EVASASPEDESPGSNP-EPDATPFQEGLRTFDQLDAISS-LP---TPSDIFVSYSTFPGFVSWRDPKSGSWYVETLDDIFEQWAHS-EDLQSLLLRVANAVSVKGI----------YKQMP-GC-FNFLRKKLFF

>Bt_CASP9

YVLNAD--PCGHCLIINNVNFC-------RESGLRARTGSNIDCERMRRRFHLLQFVVEVKCDLTAKQMVQALMQLARQ-DHSALD--CCMVVILSHGCQASHLQFPGAVYGT-----DGCPVSVERIVNTFNGTGCPSLRGKPKLFFIQACGGEQKD----HGF----------EVESTSPEDKTPSSDP-EADATPFQEGPRSIDEPDAVSS-LP---TPSDILVSYSTFPGFVSWRDPKSGSWYIETLDSIFEQWAHC-EDLQTLLLRVANAVSVKGI----------YKQMP-GC-FNFLRKKLFF

>Ta_CASP9

YSLKSE--PCGHCLIINNVDFS-------LGSELSTRTGSDVDCEKIQRRFRLLHFEVAVERNLTAEEMVKALNKLARK-DHSALD--CCVVVILSHGYQASHIQFPGGIHGT-----DGKSISVETIVNYFNGSRCPSLGGKPKLFFIQACGGEQKD----RGF----------EVNLDSPEDHPTRRDP-ESDATPFQAAPEGSDEPDAVAS-LP---TPSDILVSYSTFPGFVSWRDTKSGSWYIETLDSVLEQWAHT-EDLLTLLLRVSNGVSSKGK----------YKQIP-GC-FNFLRKRLYF

>Md_CASP9

YTLNSD--PCGYCLIINNVDFC-------ASSGLSSRKGSNIDCEKMHNRFQALHFAVEVERNLSAKGMGAALQQLAER-DHSALD--CCVVVILSHGCQASHIQFPGAIHGT-----DGASISVERIVNIFSGSRCPSLGGKPKLFFIQACGGDQRD----HGF----------QV-SSNIPEDKSPGSDPETDATPFQ---NNFDQPDAVAS-LP---TPSDILVSYSTFPGFVSWRDPKSGSWYIETLDRVLEEWADR-EDLLHMLLMVSNAVSARGT----------YKQIP-GC-FNFLRKRFFF

>Pt_CASP10

YRMNRN--HRGLCVIVNNHSFT----------SLKDRQGTHKDAEILSHVFQWLGFTVHIHNNVTKVEMEEVLQRQKCNPAHADGD--CFVFCILTHGRF-------GAVYSS-----DEALIPIREIMSHFTALQCPRLAEKPKLFFIQACQGEEIQ----PSV----------SI---------------EADALNPEKAPTSLQ-----DS-IP---AEADFLLGLATVPGYVSFRHVEEGSWYIQSLCNHLKKLVPR---MLKFLEKTMEIRGRKRTVWGAKQTQISATSLPMAISAQTPRPPMHR

>Ta_CASP10

YRMDRA--RRGYCIIFNNFTFK---------GKLNSRKGTQKDVAELQRVFRWLGLDVETFNDKTRQEMVATLEECSRRPDHDVRD--CLVCCVLSHGES-------GAVYSA-----DEELIPIRQIMSYFTAKGCPGLAHKPKLFFIQACQGKDIQ----EAV----------QIEPDAR----------NPELDPQPEPPPAPR-ESPKDS-IP---AEVDFLLGMATVDGYASFRHVYQGTWYIQALCRQLQLLVPRREDILSILTAVNDDVSQRADNLGK------KKQMP-QP-AFTLRRKLVF

>Md_CASP10

YRMDHS--HRGHCIIFNNFEFQ----------TMKRRRGSCKDADELKSVFEWLGFTVKIHHNKEKREMEDILQQCSTSPEHRESD--CFVCCVLTHGES-------GSVFSS-----DEEKIAIRELTSYFKAHRCPGLANKPKLFFFQACQGPDIQ----ESM---------------------------LLEEDAKISPP--IQVENPQRY-IP---VEADFLLGMATVDGCCAIRHTIKGSWYIQALCHRLKCMVPRNEDILTILTEVNNDVSQLTDAEGK------KKQMP-QP-AFTLRKKVVF

>Ta_CASP8

YKMNSN--PRGYCLIINNYNFQQARAEVPKLKKMKNRDGTDKDAEALRQIFKALAFLPVVLEDQTANQILETLQRFQRM-DHSAQD--CFVCCILSHGNR-------GVVYGT-----DGQQASICSLTSYFTGSRCPTLAGKPKLFFIQACQGDAYH----LAV----------AL---------------ETDTGPAAASPAEPDAVFHERC-IP---DEADFLLGMATVSNCVSYRVPSTGTWYIQSLCHHLRNLCPLGDDILTILTKVNFEVSQKIDLNNR------GKQMP-QP-MFTLRKKLVL

>Md_CASP8

YKMSSR--PRGYCLIINNFDFKISRKERPENHYLTDRRGTNKDEEALKNIFKELHFDIQSFQDLTAEGIQQVLKTFKDN-NHESKD--CFVCCLLSHGNK-------GTIYGI-----DGKEVPIRDLTSYFSGSNCPSLAGKPKVFFIQACQGKATQ----YGI----------SL---------------DTDSEQQRESLEADK-SFQSEC-IP---NEADFLLGMATVENYVSYRDSARGTWYIQSLCKNLKEGCLRGNDILTILTEVNSEVSQKTDPKNN------GKQMP-QP-KFTLRKKLVF

>Pt_CASP8

YQMKSK--PRGYCLIINNHNFAKAREKVPKLHSIRDRNGTHLDAGALTTTFEELHFEIKPHDDCTVQQIYEILKIYQLM-DHSNMD--CFICCILSHGDK-------GIIYGT-----DGQEAPIYELTSQFTGLKCPSLAGKPKVFFIQACQGDNYQ----KGI---------------------------PVETDSEEQPYLEMDLSSPQTRYIP---DEADFLLGMATVNNCVSYRNPAEGTWYIQSLCQSLRERCPRGDDILTILTEVNYEVSNKDDKKNM------GKQMP-QP-TFTLRKKLVF

>Bt_CASP8

YRMTSK--PRGYCLIFNNYDFSIARKQVPELHDLKDRTGTDFDADALDKTFRELHFEIVHYKDLTAKGICEVLESYQKK-DHKNKD--CFICCILTHGNK-------GIIYGS-----DGQEASIYELTSYFTGLKCPSLIGKPKIFFIQACQGDKYQ----KGV----------AV---------------ETDSEQMEAYLEVDS-SPQKRY-IP---DEADFLLGMATVKNYVSYRNIWNGAWYIQSLCQNLRERCPRGEDILTILTKVNFEVSKLDDKQRM------AKQMP-QP-TFTLRKKLFF

>Md_CASP18

YKMEHV--PHGYVVIIDNIHFS---------NPVDVRIGTEKDVAALRKVFGRLQFKEEYHSNLDASQLHEVMKDYSKR-DYTDQD--AFICCILSHGKK-------GVVLGT-----DWKPVAIKKLLSYFTANECKTLKDKPKLFFIQACQNGKSDSLPEVDV----------EF---------------DLEADAIC-----------SNT-TH---EWSDIFIGMATVEDSLAQRSGSIGSPYIQNLCKELEAHCPQKKELLEIMTSVNSKVSN-------------IIQMP-EF-RSTLRAPFIF

>Ta_CASP18

YKMSHL--PHGFCVIINNSEFQ---------NPQNTRRGADKDAAALDKVFSWLQFKVEHHSNLKGEAITRVLRAYSER-DHTDHD--CFICCLLSHGQK-------GEILGT-----DWEPVPLRALLGLFTSSACRTLAAKPKLFFVQACQGGQGQ----GGL---------SLRGHDDA----------LLESDAVV-----------IPS-IP---DWADLLVGMATVEDFICFRRPCHGSEYIQALCRALETFCPRGNDLLTILTHVNKKVGQKVFGN--------NKQMP-EV-KFTLQRPLIF

>Ta_CASP6

YKMEYK--RRGVALIFNHERFY-------WQLKLPERRGTRADRENLTRRLSALGFEVKCFDDLKAEEVMLKIHEASSS-NHADAD--CFLCVFLSHGLG-------NHVYAY-----DG-KIDIPEITSMFKGDQCRSLVGKPKIFIFQACRGEEHD----VGV-------VPLDV-LDSK----------MSEQDVNETEVDAAS----VYT-LP---AGADFLMCYSVAEGYYSHRETVNGSWYVQDLCEMLGRYGSS-LEFTELLTLVNRKVSYRRVDFCNNPRAIGKKQIP-CF-ASMLTKKLHF

>Md_CASP6

YKMDHK--RRGVALIFNHERFF-------WHLTLPERRGTRADRDNLRQRLSDLGFEVNCFDDLKAEDLLMTIHMVSTS-SHIDAD--CFLCVFLTHGEG-------NHIYAY-----DA-KIDIQKLTAMFKGDKCQTLVGKPKIFVIQACRGDQHD----IPV-----------IPLDVV----------DHLSDKVDVNETEVD-AASVYT-LP---AGADFLMCYSVAEGYYSHRETVHGSWYIQDLCEMLEKYGSS-LEFTELLTLVNRKVSQRRVDFCKDPNAIGKKQVP-CF-ASMLTKKLYL

>Pt_CASP6

YKMDHR--RRGIALIFNHERFF-------WHLTLPERRGTCADRDNLTRRFSDLGFEVKCFNDLKAEELLLKIHEASTV-SHADAD--CFVCVFLSHGEG-------NHIYAY-----DA-KIEIQTLTGLFKGDKCHSLVGKPKIFIIQACRGNQHD----VPV-----------IPLDVV----------DNQTEKLDTNITEVD-AASVYT-LP---AGADFLMCYSVAEGYYSHRETVNGSWYIQDLCEMLGKYGSS-LEFTELLTLVNRKVSQRRVDFCKDPSAIGKKQVP-CF-ASMLTKKLHF

>Bt_CASP6

YKMDHK--RRGIALIFNHERFF-------WHLTLPNRPGTSADRDNLRRRFSDLGFEVKCFDDLRAEELLLKIHEASTA-SHVDAD--CFLCVFLSHGEG-------NHIYAY-----DA-KIEIQTLTGLFKGDKCQSLVGKPKIFIIQACRGSQHD----VPV-----------IPLDVV----------DHRTDTPDANLTQVD-AASVYT-LP---AGADFLMCYSVAEGYYSHRETVNGSWYIQDLCEMLGKFGSS-LEFTELLTLVNRKVSQRRVDFCRDPNAIGKKQVP-CF-ASMLTKKLHF

>Ta_CASP3

YRMDYP--NMGICLIINNKNFH-------PNTGMGCRSGTDVDAASLIDTFKKLKYEVRCKNDMKRHEILELLTSVAHE-DHSKRS--SFVCVLLSHGEE-------GVIFGT-----DG-SLELKSLANLFRGDNCRSLVGKPKLFIVQACRGTELD----SGV---------------------------EADSSSAD--------DGPEQK-IP---VEADFLYAYSTAPGYYSWRNSLNGSWFIQALCAMLKQHAPT-LELLHILTRVNRKVATEFESYSPDASFHAKKQIP-CI-VSMLTKELYF

>Md_CASP3

YKMDYP--EMGLCIIINNKNFH-------PNTGMSFRSGTDVDAASLSDTFRSLKYEVRIKNDLTCNEITELLNSVSKE-DHSQRS--SFICVILSHGEE-------GVIFGT-----DR-SVELKRLTCFFRGDKCRSLTGKPKLFIIQACRGTELD----CGV---------------------------ETDSGTDE--------DIACQK-IP---VEADFLYAYSTAPGYYSWRNSKDGSWFIQALCAVLKQHAHK-LEIMQILTRVNRKVATEFESYSLDISFHAKKQVP-CI-MSMLTKELYF

>Pt_CASP3

YKMDYP--EMGLCIIINNKNFH-------KSTGMTSRSGTDVDAANLRETFRNLKYEVRNKNDLTREEIVELMRDVSKE-DHSKRS--SFVCVLLSHGEE-------GIIFGT-----NG-PVDLKKITNFFRGDRCRSLTGKPKLFIIQACRGTELD----CGI---------------------------ETDSGVDD--------DMACHK-IP---VEADFLYAYSTAPGYYSWRNSKDGSWFIQSLCAMLKQYADK-LEFMHILTRVNRKVATEFESFSFDATFHAKKQIP-CI-VSMLTKELYF

>Bt_CASP3

YKMDYP--EMGLCIIINNKNFH-------ENTGMACRSGTDVDAANLRETFMNLKYEVRIKNDLTCKEMLELMSNVSKE-DHSKRS--SFICVLLSHGEE-------GIIFGT-----NG-PVNLKKLASFFRGDYCRSLTGKPKLFIIQACRGTELD----CGI---------------------------ETDSGAED--------DMACQK-IP---VEADFLYAYSTAPGYFSWRNAKNGSWFIQVLCEMLKKYAHR-LELMHILTRVNRKVAIEYESFSTDSAFHAKKQIP-CI-MSMLTKELYF

>Ta_CASP7

YKMNYE--NLGKCIIINNKNFD-------SKTGMNTRNGTDKDAGSLQKCFRNLGFDVSVFNDFSCNQMKEILQKASEE-NHENSA--CFACILLSHGEG-------DCIYGT-----DD-CIPIKELTILFRGDKCKSLLGKPKLFFIQSCRGTEFD----DGI----------QT-DSTP----------VTDPDANP-----------GCR-IP---VEADFLFAYSTVPGYYSWRNVERGSWFVQALCSVLNDHGKE-LEIMQLLTRVNHQVAINFESWCSDPYYCQKKQIP-CL-VSMLTKELYF

>Md_CASP7

YNMDYK--KVGKCIIINNKQFD-------SKTGMGTRNGTDKDAEGLTKCFRSLGFDVIVYNNRSCSDMRNLLKQVSQE-DHTESA--CFACILLSHGEE-------DLIYGT-----DG-VTPIKDLTGHFRGDKCKSLLGKPKLFFIQACRGTEFD----DGI----------QT-DSGP----------INDTNANP-----------GCK-IP---VEADFLFAYSTVPGYYSWRSQAKGSWFVQALCSVLNEHGKS-LEIMQILTRVNYMVAMDFESQSDNPCFNEKKQIP-CM-VSMLTKELYF

>Pt_CASP7

YNMNFE--KLGKCIIINNKNFD-------KVTGMGVRNGTDKDAEALFKCFRSLGFDVIVYNDCSCAKMQDLLKKASEE-DHTNAA--CFACILLSHGEE-------NVIYGK-----DG-VTPIKDLTAHFRGDRCKTLLEKPKLFFIQACRGTELD----DGI----------QA-DSGP----------INDTDANP-----------RYK-IP---VEADFLFAYSTVPGYYSWRSPGRGSWFVQALCSILEEHGKD-LEIMQILTRVNDRVARHFESQSDDPRFHEKKQIP-CV-VSMLTKELYF

>Bt_CASP7

YNMNFE--KVGKCIIINNKNFD-------RITGMGVRNGTDKDAEALFKCFRSLGFDVSVYNDCSCAKMQDLLKKASEE-DHRNSA--CFACILLSHGEE-------NLIYGT-----DG-KTAIKDLTAHFRGDRCKTLLEKPKLFFIQACRGTELD----DGI----------QA-DSGP----------INDTDANP-----------RYK-IP---VEADFLFAYSTVPGYYSWRNPGSGSWFVQALCSILNEHGKS-LEILQILTRVNDRVARHFESQSDDPRFHEKKQIP-CV-VSMLTKELYF

**Supplementary Figure S5. Amino acid sequences and alignment used for phylogenetic analysis of caspases. (A)** Amino acid sequences in FASTA format. All sequences have downloaded from GenBank. Accession numbers are added in the FASTA title line only when necessary for clearly linking the sequence to the database. **(B)** Amino acid sequence alignment in FASTA format. Species: Bt, *Bos*

*tauru*s, cattle; Md, *Monodelphis domestica*, opossum; Pt, *Pan troglodytes*, chimpanzee; Ta, *Tachyglossus aculeatus*, echidna.


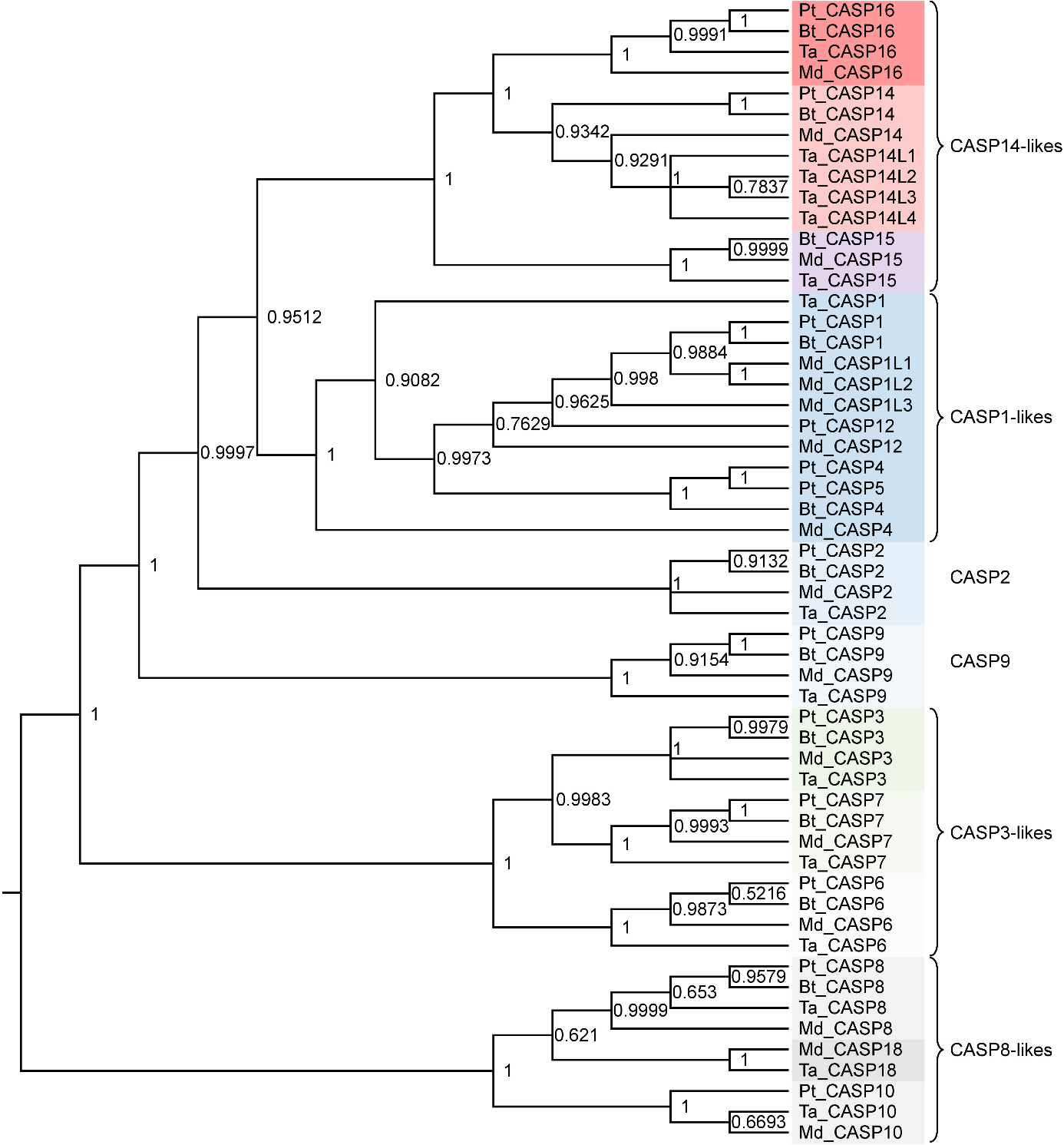


**Supplementary Figure S6. Phylogenetic analysis of mammalian caspases according to Bayesian inference.** The caspases (CASPs) of chimpanzee (*Pan troglodytes*, Pt), cattle (*Bos taurus*, Bt), opossum (*Monodelphis domestica*, Md) and echidna (*Tachyglossus aculeatus*, Ta) were subjected to Bayesian inference analysis based on the amino acid sequence alignment of the caspase domain. Values at the nodes indicate the posterior probabilities. Paralogs of CASP14 of the echidna are named CASP14-like (CASP14L) 1 through 4, and paralogs of CASP1 of the opossum are named CASP1-like (CASP1L) 1 through 3. On the right, the main clades of caspases are labelled according to the best characterized member of each clade (e.g. CASP1-likes).

Chimp 1 AGCTTGGTGCTTGGAGGAAGCCCCGTCTGGCCCTATGGCCTTCCTGGTGGCTGGGACCCT 60

|||||||||||||||||||||||||||| |||||||||||||||||||||||||||||||

Human 1 AGCTTGGTGCTTGGAGGAAGCCCCGTCTCGCCCTATGGCCTTCCTGGTGGCTGGGACCCT 60

Chimp 61 GCGGCTGGCCATGCAGATGGCAGACACTCGAGACAGCCTGGGGAGAAAGGTAGCAGGAGG 120

||||||||||| ||||||||||||||||||||||||||||||||||||||||||||||||

Human 61 GCGGCTGGCCACGCAGATGGCAGACACTCGAGACAGCCTGGGGAGAAAGGTAGCAGGAGG 120

Chimp 121 CACCGCCTAGCCCAGCTGCTCCTGC--------TGAG---C--CCCAGCCCAGCCCAGCC 167

||||||||||||||||||||||||| |||| | |||||||||||||||||

Human 121 CACCGCCTAGCCCAGCTGCTCCTGCCTCCCTGCTGAGCCCCAGCCCAGCCCAGCCCAGCC 180

Chimp 168 CAGCTCTGGCCTTGCTTCCCCAGGGGAAGTATGACGTCCAGGGTCCAAGGGCAGCCCTGA 227

||||||||||||||| ||||||||||||||||||||||||||||||||||||||||||||

Human 181 CAGCTCTGGCCTTGCCTCCCCAGGGGAAGTATGACGTCCAGGGTCCAAGGGCAGCCCTGA 240

Chimp 228 TGCTCAGCAGCCCTGGGGTGGCGGCCGCTGTAGTCACTGCCCTGGAGGACGTGTTCCAGG 287

||||||||||||||||||||||||||||||||||||||||||||||||||||||||||||

Human 241 TGCTCAGCAGCCCTGGGGTGGCGGCCGCTGTAGTCACTGCCCTGGAGGACGTGTTCCAGG 300

Chimp 288 CCCTGGGCTTTGAGAGCTGCGCGAGGAGGGAGGTCCCGGTCCAGGTGAGCCTCTGCCTCT 347

||||||||||||||||||||| ||||||||||||||||||||||||||||||||||||||

Human 301 CCCTGGGCTTTGAGAGCTGCGAGAGGAGGGAGGTCCCGGTCCAGGTGAGCCTCTGCCTCT 360

Chimp 348 TACATCCACCCTCAGGCCCAGCACCGaccccttccccactctcccctgacccagaatctc 407

||||||||||||||||||||||||||||||||||||||||||||||||||||||||||||

Human 361 TACATCCACCCTCAGGCCCAGCACCGACCCCTTCCCCACTCTCCCCTGACCCAGAATCTC 420

Chimp 408 ctccccacccaaaacctccccGGACTCAGGTCGGTCTCACTCTTGCCCCTAGGGCTTCCT 467

||||||||||||||||||||||||||||||||||||||||||||||||||||||||||||

Human 421 CTCCCCACCCAAAACCTCCCCGGACTCAGGTCGGTCTCACTCTTGCCCCTAGGGCTTCCT 480

Chimp 468 CGAGGAACTGGCTTGGTTCCAGGAGCAGCTGGATGCCCACGGGCGCCCTGTGGGGTGTGC 527

||||||||||||||||||||||||||||||||||||||||||||||||||||||||||||

Human 481 CGAGGAACTGGCTTGGTTCCAGGAGCAGCTGGATGCCCACGGGCGCCCTGTGGGGTGTGC 540

Chimp 528 CTTAGTGGCCTTGGTGGCCCCCAGAGGGCAGCTGAGGCAGCCACAGCAGCTGGTCCGGGA 587

||||||||||||| | ||||||||||||||||||||||||||||||||||||||||||||

Human 541 CTTAGTGGCCTTGAT-GCCCCCAGAGGGCAGCTGAGGCAGCCACAGCAGCTGGTCCGGGA 599

Chimp 588 GCTGAGCGGCTGCCGGGCCCTGCGGGGCTGCCCCAAAGTCTTCCTGCTGCTCTCAAGTGG 647

||||||||||||||||||||||||||||||||||||||||||||||||||||||||||||

Human 600 GCTGAGCGGCTGCCGGGCCCTGCGGGGCTGCCCCAAAGTCTTCCTGCTGCTCTCAAGTGG 659

Chimp 648 CCCTGGGTGTGAGTGAGCTGGGTCAGGATCCAGGAGCTGGGCAGGGACCCAGGGGCAGAG 707

|||||||||||||||||||||||||||||||||||||||||||||||||||||||||||

Human 660 TCCTGGGTGTGAGTGAGCTGGGTCAGGATCCAGGAGCTGGGCAGGGACCCAGGGGCAGAG 719

Chimp 708 CCTCGGGCCTCACTGCAGGCCAACATGCTGCTTCTCCACCCAGCCTCCCTGGAGCCCGGA 767

|||||||||||||||||||||||||||||||||||| |||||||||||||||||||||||

Human 720 CCTCGGGCCTCACTGCAGGCCAACATGCTGCTTCTCTACCCAGCCTCCCTGGAGCCCGGA 779

Chimp 768 GCCTTCCTTGCTGGCCTGAGAGAGCTGTGTGGCCGCTCTCCTCACTGGTCCCTGGTGCAG 827

||||||||||||||||||||||||||||||||||||||||||||||||||||||||||||

Human 780 GCCTTCCTTGCTGGCCTGAGAGAGCTGTGTGGCCGCTCTCCTCACTGGTCCCTGGTGCAG 839

Chimp 828 CTGCTGACGGAGGTGGGGACGCTGGAGGGGGAGGCCCAGGGAAGCGGGGCTGGTCCTGCT 887

||||||||| ||||||||||||||||||||||||||||||||||||||||||||||||||

Human 840 CTGCTGACGAAGGTGGGGACGCTGGAGGGGGAGGCCCAGGGAAGCGGGGCTGGTCCTGCT 899

Chimp 888 GTCTCTGCTGGTTCTGCTGTGCCCCCCTAAGCCAGTTACAGTTAGATTCATTCACTTGTA 947

||||| ||||||||||||||||||||||||||||||||||||||||||||||||||||||

Human 900 GTCTCCGCTGGTTCTGCTGTGCCCCCCTAAGCCAGTTACAGTTAGATTCATTCACTTGTA 959

Chimp 948 TCCTCCCTCCGATTCATTCTACAAACCTCCTTTATTGTCTACAGAAGTGGTCTCAGCCCC 1007

|||||||||||||||||||||||||||||||||||| |||||||||||||||||||||||

Human 960 TCCTCCCTCCGATTCATTCTACAAACCTCCTTTATTTTCTACAGAAGTGGTCTCAGCCCC 1019

Chimp 1008 TCACCCTCAACTCGGGACCCCTGACAACCCACTCAGGATCCCCGACACTCGACTCCTCCC 1067

||||||||||||||||||||||||||||||||||||||||||||||||||||||||||||

Human 1020 TCACCCTCAACTCGGGACCCCTGACAACCCACTCAGGATCCCCGACACTCGACTCCTCCC 1079

Chimp 1068 AACTCCATGCCTTGGCCCAAGCGGTTCCCTCTGCCTGCTATGCCTTCCTGTTGTCCCGGG 1127

| |||||||||||||||||||| |||||||||||||||||||||||||||||||||||||

Human 1080 AGCTCCATGCCTTGGCCCAAGCAGTTCCCTCTGCCTGCTATGCCTTCCTGTTGTCCCGGG 1139

Chimp 1128 AGCCCTGCACAGTCCTCTGCAGGCTGCCAGCGATGCCCTTCCTTGCATCCACGCTTGCAC 1187

||||||||||||||||||||||||||||||||||||||||||||||||||||||||||||

Human 1140 AGCCCTGCACAGTCCTCTGCAGGCTGCCAGCGATGCCCTTCCTTGCATCCACGCTTGCAC 1199

Chimp 1188 TTCAGGCTGCCCCTTTCTACACCAAGCAGTGGCTGTCCGCCCTGTTAGATCGCAGGACCC 1247

||||||||||||||||||||||||||||||||||||||||||||||||||||||||||||

Human 1200 TTCAGGCTGCCCCTTTCTACACCAAGCAGTGGCTGTCCGCCCTGTTAGATCGCAGGACCC 1259

Chimp 1248 TGGAGGCTGGGACTGGGAATGCTTCAAGTCTGGTCTCCAAGGCTGAAGGTCTCACTGTGT 1307

||||||||||||||||||||||||||||||||||||||||||||||||||||||||||||

Human 1260 TGGAGGCTGGGACTGGGAATGCTTCAAGTCTGGTCTCCAAGGCTGAAGGTCTCACTGTGT 1319

Chimp 1308 AGTTGGGTGGCCCCTGGGCAGCCCTGGGGATGAGGGGGCCCCAGCTCTCCTCCACAGATC 1367

||||||||||||||||||||||||||||||||||||||||||||||||||||||||||||

Human 1320 AGTTGGGTGGCCCCTGGGCAGCCCTGGGGATGAGGGGGCCCCAGCTCTCCTCCACAGATC 1379

Chimp 1368 GCCCAGCAGGGTAGTGCCCACCCCGTGAGTCTTCCTGACAGGCCCAGCCCCGCTCTAGGG 1427

||||||||||||||||||||||||||||||||||| ||||||||||||||||||||||||

Human 1380 GCCCAGCAGGGTAGTGCCCACCCCGTGAGTCTTCCCGACAGGCCCAGCCCCGCTCTAGGG 1439

Chimp 1428 AACATCCCCACTTCTCTGCGCACTGATCCCGGGGCAGGGGTTAATGGTGTGGCCACTGTC 1487

||||||||||| ||||||||||||||||||||||||||||||||||||||||||||||||

Human 1440 AACATCCCCACCTCTCTGCGCACTGATCCCGGGGCAGGGGTTAATGGTGTGGCCACTGTC 1499

Chimp 1488 CACTCGAGGCCATCCATGCCCAGGGAAGTCCATAACCTTGAGCCTGAACTGGAGCCTGGG 1547

||||||||||||||||||||||||||||||||||||||||||||||||||||||||||||

Human 1500 CACTCGAGGCCATCCATGCCCAGGGAAGTCCATAACCTTGAGCCTGAACTGGAGCCTGGG 1559

Chimp 1548 CCTTGCTTGAGGCCATCCTGCCTGTCTGTCCTTTGCTGGCCTCCTCCTGTCTGCATTCTG 1607

|| |||||||||||||||||||||||||||||||||||||||||||||||||||||||||

Human 1560 CCCTGCTTGAGGCCATCCTGCCTGTCTGTCCTTTGCTGGCCTCCTCCTGTCTGCATTCTG 1619

Chimp 1608 GCAGGTGACCCTGAAGAGACTACAGAAGCCACAAGCCTGGCTGAGTCTTTTCTCGGCCCC 1667

|||||||||||||||||||||| |||||||||||||||||||||||||||||||||||||

Human 1620 GCAGGTGACCCTGAAGAGACTATAGAAGCCACAAGCCTGGCTGAGTCTTTTCTCGGCCCC 1679

Chimp 1668 ATCCCCAGCTCTTCCGCAGGATGGCTGAAGAGTCCGCAGGGGGCACCTGCTGCCCCGTCC 1727

|||||||||||||||||||| |||||||||||||||||||||||||||||||||||||||

Human 1680 ATCCCCAGCTCTTCCGCAGGGTGGCTGAAGAGTCCGCAGGGGGCACCTGCTGCCCCGTCC 1739

Chimp 1728 TTAGGAGCTCCTTGAGGGGGGCACTGTGCCTGGGAGGCGTGGAGCCCTGGAGGCCTGAGG 1787

|| |||||||||||||||||||||||||||||||||||||||||||||||||||||||||

Human 1740 TTCGGAGCTCCTTGAGGGGGGCACTGTGCCTGGGAGGCGTGGAGCCCTGGAGGCCTGAGG 1799

Chimp 1788 TGAGGGGGGCAGGGCAGGGGTCCAATCACATGGCCACAGTTTCCAGTGGGACAGAAGCTT 1847

||||||||||||||||||| ||||||||||||||||||||||||||||||||||||||||

Human 1800 TGAGGGGGGCAGGGCAGGGATCCAATCACATGGCCACAGTTTCCAGTGGGACAGAAGCTT 1859

Chimp 1848 AGGGGGA-CCCAGGCCGGGGAGGCCAGGAGCTGGAGTCCTCTCAGGCCACTTTAGATGTG 1906

|||||| ||| ||||||||||||||||||||||||||||||||||||||||||||||||

Human 1860 AGGGGGGGCCCCGGCCGGGGAGGCCAGGAGCTGGAGTCCTCTCAGGCCACTTTAGATGTG 1919

Chimp 1907 TCTTAACCCTCTCTGCAGCCGGCCCCCGGTCCCAGCACACAGTATGACCTGTCCAAGGCC 1966

||||||||||||||||||||||||||||||||||||||||||||||||||||||||||||

Human 1920 TCTTAACCCTCTCTGCAGCCGGCCCCCGGTCCCAGCACACAGTATGACCTGTCCAAGGCC 1979

Chimp 1967 AGGGCTGCCCTCCTCCTGGCTGTGATCCAAGGCCGGCCTGGGGCCCAGCATGACGTGGAG 2026

||||||||||||||||||||||||||||||||||||||||||||||||||||||||||||

Human 1980 AGGGCTGCCCTCCTCCTGGCTGTGATCCAAGGCCGGCCTGGGGCCCAGCATGACGTGGAG 2039

Chimp 2027 GCGCTGGGGGGCCTGTGCCGGGCCCTGGGCTTTGAGACCACCGTGAGAACGGACCCTACA 2086

|||||||||||||||||| |||||||||||||||||||||||||||||||||||||||||

Human 2040 GCGCTGGGGGGCCTGTGCTGGGCCCTGGGCTTTGAGACCACCGTGAGAACGGACCCTACA 2099

Chimp 2087 GCTCAGGTGAGGGGAAGCCGAGAACTTCCGCTGGTGCTCTGAAGGAAGACCACCCCTCCC 2146

|| |||||||||||||||||||||||||| ||||||||||||||||||||||||||||||

Human 2100 GCCCAGGTGAGGGGAAGCCGAGAACTTCCACTGGTGCTCTGAAGGAAGACCACCCCTCCC 2159

Chimp 2147 TAGAAACCTGGGGCCTCTCTCCTGGGCCTCTCTCCATCACCGGCAGGAAGTGCACCACAA 2206

|||||||||| ||||||||||||||||| |||||||||||||||||||

Human 2160 TAGAAACCTG-------------GGGCCTCTCTCCATCACTGGCAGGAAGTGCACCACAA 2206

Chimp 2207 GTCTAGCCTCTGTGCTCCCTGTTGCCTGCATCTGGTCCGTCACTTCCCTGCCTCTGGAAG 2266

||||| ||||||||||||||||||||||||||||| ||||||||||||||||||||||||

Human 2207 GTCTAACCTCTGTGCTCCCTGTTGCCTGCATCTGGCCCGTCACTTCCCTGCCTCTGGAAG 2266

Chimp 2267 CCTGGTCTCCAGGGTCCCCGAGGCCTCCCTCACTGGCTGGTTCTCTGG-ccccccgcccc 2325

|||||||||||||||||||||||||| ||||||||||||||||||||| |||||||||||

Human 2267 CCTGGTCTCCAGGGTCCCCGAGGCCTTCCTCACTGGCTGGTTCTCTGGCCCCCCCGCCCC 2326

Chimp 2326 ctccccAGCTCAAAGCTTTAGCTCCAAGTCTTGGTTTCCCTCTTGGCTCCCAGCAGCCCA 2385

||||||||||||||||||||||||||||||||||||||||||||||||||||||||||||

Human 2327 CTCCCCAGCTCAAAGCTTTAGCTCCAAGTCTTGGTTTCCCTCTTGGCTCCCAGCAGCCCA 2386

Chimp 2386 CTCCGCTCTCCTCACACCTCTCAACTTCTTGGTGCGGCTTCCCCACGAGGGCAGGAGGAG 2445

|||| |||||||||||||||||||||||||||||||||||||||||||||||||||||||

Human 2387 CTCCACTCTCCTCACACCTCTCAACTTCTTGGTGCGGCTTCCCCACGAGGGCAGGAGGAG 2446

Chimp 2446 AACTGGCTCCAGGAAGCTGGGTCTCTACGTCACCTCTAAAGAGGCCATGCCAAGGCCTTG 2505

||||||||||||||||||||||||||| ||||||||||||||||||||||||||||||||

Human 2447 AACTGGCTCCAGGAAGCTGGGTCTCTATGTCACCTCTAAAGAGGCCATGCCAAGGCCTTG 2506

Chimp 2506 CAGGAGGGAGTTAGAAAAGGGCTTCTGGCCGGGCGCAGTGGCTCACGCCTGTAATCCCAA 2565

||||||||||||||||||||||||||||||||| ||||||||||||||||||||||||||

Human 2507 CAGGAGGGAGTTAGAAAAGGGCTTCTGGCCGGGTGCAGTGGCTCACGCCTGTAATCCCAA 2566

Chimp 2566 AACTTTAGGAGGCTGAGACGGGTGGATCACTTGAGATTAGGAGTTTGAGACCAGCCTGAC 2625

||||||||||||||||||||||||||||||||||||||||||||||||||||||||||

Human 2567 CGCTTTAGGAGGCTGAGACGGGTGGATCACTTGAGATTAGGAGTTTGAGACCAGCCTGAC 2626

Chimp 2626 TACCACGGTGAAACCTCGTCTCTACTATAAAGACAAAATTAGTAGGGCATGGTGGTGCGT 2685

||||| ||||||||||||||||||||||| ||||||||||||||||||||||||||||||

Human 2627 TACCATGGTGAAACCTCGTCTCTACTATATAGACAAAATTAGTAGGGCATGGTGGTGCGT 2686

Chimp 2686 GCCTGTAATCCCAGCTACTTGGGAGGCTGAGGCAGGAGAATGGCTTGAACCCAGGAGGCC 2745

||||||||||||||||||||||||||||||||||||||||||||||||||||||||||||

Human 2687 GCCTGTAATCCCAGCTACTTGGGAGGCTGAGGCAGGAGAATGGCTTGAACCCAGGAGGCC 2746

Chimp 2746 GAGGTTGCAGTGAGCTGAGATCGGGCCACTGCACTCCAGCCTGGGCGACAGAGCAAGACT 2805

||||||||||||||||||||| | ||||||||||||||||||||||||||||||||||||

Human 2747 GAGGTTGCAGTGAGCTGAGATTGTGCCACTGCACTCCAGCCTGGGCGACAGAGCAAGACT 2806

Chimp 2806 CCGTCTCaaaaaacaaaaaCAGGCCGGGCACAGTGGCTCACGCCTGTAATCCCAGCACTT 2865

| ||||||||||||||||||||||||||||||||||||||||||||||||||||||||||

Human 2807 CTGTCTCAAAAAACAAAAACAGGCCGGGCACAGTGGCTCACGCCTGTAATCCCAGCACTT 2866

Chimp 2866 TGGGAGGCCGAGGCAAGCGGATCACGACGTCAGGAGATCGAGACCATTCTGGCTAACACA 2925

|||||||||||| |||||||||||||||||||||||||||||||||||||||||||||||

Human 2867 TGGGAGGCCGAGACAAGCGGATCACGACGTCAGGAGATCGAGACCATTCTGGCTAACACA 2926

Chimp 2926 GCGAAACCTCATCTCTACTAAAAATACAAAAAATTAGCCGGGTGTGGTGGCATATGCCTG 2985

||||||||||||||||||||||||||||||||||||||||||||||||||||| | ||||

Human 2927 GCGAAACCTCATCTCTACTAAAAATACAAAAAATTAGCCGGGTGTGGTGGCATGTACCTG 2986

Chimp 2986 TATTCCCAGCTACTCAGGAGGCTGAGGCAGGAGAATCGCGTGAACCCAGGAGGCAGAGAT 3045

||||||||||||||||||||||||||||||||||||||| ||||||||||||||||||||

Human 2987 TATTCCCAGCTACTCAGGAGGCTGAGGCAGGAGAATCGCTTGAACCCAGGAGGCAGAGAT 3046

Chimp 3046 AGCAGTGAGCCGAGATCGCGCCACTGCACTCCAGCCTGGGTGACAGAGCAAGACTCCTTC 3105

||||||||||||||||||||||||||||||||||||||||||||||||||||||||||||

Human 3047 AGCAGTGAGCCGAGATCGCGCCACTGCACTCCAGCCTGGGTGACAGAGCAAGACTCCTTC 3106

Chimp 3106 TCaaaaaaaaaaaaaaaaaaaaaaaGAGGGCTTCCTCCCTGGACCTGTGAGTGGCAGGCG 3165

| ||||||||||||||||||||||||||||||||||||||||||||||||||||||||

Human 3107 CC--AAAAAAAAAAAAAAAAAAAAAGAGGGCTTCCTCCCTGGACCTGTGAGTGGCAGGCG 3164

Chimp 3166 GTGGGAGGCCAGGTGGGGCAGGGTCTGGGAAACCTTGTCAGCCTCACAGAGGGCAGCCAG 3225

||||||||||||||||||||||||||||||||||||||||||||||||||||||||||||

Human 3165 GTGGGAGGCCAGGTGGGGCAGGGTCTGGGAAACCTTGTCAGCCTCACAGAGGGCAGCCAG 3224

Chimp 3226 TGGCTGGGGAGGTGGTGGCTTTGGCCCAGGCCTCAACATTGTTCCCACCCCAGGCTTTCC 3285

|||||||||||| |||||||||||||||||||||||||||||||||||||||||||||||

Human 3225 TGGCTGGGGAGGCGGTGGCTTTGGCCCAGGCCTCAACATTGTTCCCACCCCAGGCTTTCC 3284

Chimp 3286 AGGAGCAGCTGGCCCAGTTCCGGGAGCAACTGGACACCTGCAGGGGCCCTGTGAGCTGTG 3345

||||| ||||||||||||||||||||||||||||||||||||||||||||||||||||||

Human 3285 AGGAGGAGCTGGCCCAGTTCCGGGAGCAACTGGACACCTGCAGGGGCCCTGTGAGCTGTG 3344

Chimp 3346 CCCTTGTGGCCCTGATGGCCCATGGGGGACCACGGGGTCAGCTGCTGGGGGCTGACGGGC 3405

||||||||||||||||||||||||||||||||||||||||||||||||||||||||||||

Human 3345 CCCTTGTGGCCCTGATGGCCCATGGGGGACCACGGGGTCAGCTGCTGGGGGCTGACGGGC 3404

Chimp 3406 AAGAGGTGCAGCCCGAGGCACTCATGCAGGAGCTGAGCCGCTGCCAGGTGCTGCAGGGCC 3465

||||||||||||||||||||||||||||||||||||||||||||||||||||||||||||

Human 3405 AAGAGGTGCAGCCCGAGGCACTCATGCAGGAGCTGAGCCGCTGCCAGGTGCTGCAGGGCC 3464

Chimp 3466 GCCCCAAGATCTTCCTGTTGCAGGCCTGCCGTGGGGGTGAGCGGCCCGGCCTCCTACTGC 3525

||||||||||||||||||||||||||||||||||||||||||||||||||||||||||||

Human 3465 GCCCCAAGATCTTCCTGTTGCAGGCCTGCCGTGGGGGTGAGCGGCCCGGCCTCCTACTGC 3524

Chimp 3526 CCTCACTTTCCTCGGCCAAGCTTCAGCCCCCGGGACTCACGGTCTACCTTCTCCAGGGAG 3585

|||||||||||||||||||||||||||||||||||||||| |||||||||||||||||||

Human 3525 CCTCACTTTCCTCGGCCAAGCTTCAGCCCCCGGGACTCACTGTCTACCTTCTCCAGGGAG 3584

Chimp 3586 CCCGGGTACCCGCCCTTCCCTGCCCCCTCTCCTGTCCTATCCTAGAGGTCAAGTCCACGA 3645

|||||||||| ||||||||||||||||||||||||||| |||||||||||||||||||||

Human 3585 CCCGGGTACCTGCCCTTCCCTGCCCCCTCTCCTGTCCTCTCCTAGAGGTCAAGTCCACGA 3644

Chimp 3646 CCTTGAACCCTTAACTCTCAACACCTGTCATTCAGCGCTCTGAATGTCTCCAGTCTGGCA 3705

||||||||||||||||||||||||||||||||||||||||||||||||||||||||||||

Human 3645 CCTTGAACCCTTAACTCTCAACACCTGTCATTCAGCGCTCTGAATGTCTCCAGTCTGGCA 3704

Chimp 3706 AGCCTGCCCTGGAGCTCTGGAGTTGGGTTCTCACCTTGACCCCACATTCACACTAGACCC 3765

||||||||||||||||||||||||||||||||||||||||||||||||||||||||||||

Human 3705 AGCCTGCCCTGGAGCTCTGGAGTTGGGTTCTCACCTTGACCCCACATTCACACTAGACCC 3764

Chimp 3766 CTGAGCACCCCCAGGTATCCCCGGAGTGAGACTATCTGCCTCTCCCCACCCTCTTCAGGA 3825

||||||||||||||||||||||||||||||||||||||||||||||||||||||||||||

Human 3765 CTGAGCACCCCCAGGTATCCCCGGAGTGAGACTATCTGCCTCTCCCCACCCTCTTCAGGA 3824

Chimp 3826 AACAGGGATGCTGGTGTGGGGCCCACAGCTCTCCCCTGGTACTGGAGCTGGCTGCGGGCA 3885

||||||||||||||||||||||||||||||||||||||||||||||||||||||||||||

Human 3825 AACAGGGATGCTGGTGTGGGGCCCACAGCTCTCCCCTGGTACTGGAGCTGGCTGCGGGCA 3884

Chimp 3886 CCTCCATCTGTCCCCTCCCATGCAGACGTCCTGCAGATCTACGCTGAGGCCCAAGGTGGG 3945

|||||||||||||||||||||||||| |||||||||||||||||||||||||||||||||

Human 3885 CCTCCATCTGTCCCCTCCCATGCAGATGTCCTGCAGATCTACGCTGAGGCCCAAGGTGGG 3944

Chimp 3946 TTCTGCCTTCCTTCCAGGGCCTGGGCTTGGGCAGGGCTGGTTGTGGGGACTGTCCAGAGA 4005

|||||||||||||||||||||||||||||||||||||||||||||||||| |||||||||

Human 3945 TTCTGCCTTCCTTCCAGGGCCTGGGCTTGGGCAGGGCTGGTTGTGGGGACCGTCCAGAGA 4004

Chimp 4006 GCATCTCCAGGGCTCTAAGCTGGGGTATGGCTGCCACCTGCATCCTCTGTTTGCCAAGAC 4065

||||||||||||||||||||||||||||||||||||||||||||||||||||||||||||

Human 4005 GCATCTCCAGGGCTCTAAGCTGGGGTATGGCTGCCACCTGCATCCTCTGTTTGCCAAGAC 4064

Chimp 4066 AATGGGAAG-aaaaaaaTCTTCCTAAACCGCAAGGGCCTTTGGGAAGTGGGAGCTTCTTC 4124

||||||||| ||||||||||||||||||||||||||||||||||||||||||||||||||

Human 4065 AATGGGAAGAAAAAAAATCTTCCTAAACCGCAAGGGCCTTTGGGAAGTGGGAGCTTCTTC 4124

Chimp 4125 CCCTGTTGGAGCCTGGCAAGAACGCTGGAGTCGGCAAGGTCAAATAGCTTTTCTGAGGTC 4184

||||||||||||||||||||||| |||||||||| |||||||||||||||||||||||||

Human 4125 CCCTGTTGGAGCCTGGCAAGAACCCTGGAGTCGGTAAGGTCAAATAGCTTTTCTGAGGTC 4184

Chimp 4185 ACAGCTGGTAAGTGGCTGGGCCGAGCTTTGAACTTCCGTCTGTCATTCCTGCCCTGCACT 4244

||||||| ||||||||||||||||||||||||||||||||||||||||||||||||||||

Human 4185 ACAGCTGTTAAGTGGCTGGGCCGAGCTTTGAACTTCCGTCTGTCATTCCTGCCCTGCACT 4244

Chimp 4245 CTTTCCACCTCCCTGGGCTGCCCTTAAGCCACAGATGGGGAGCTCCCGGGGCTGATGGAG 4304

||||||||||||||||||||||||||||||||||||||||||||||||||||||||||||

Human 4245 CTTTCCACCTCCCTGGGCTGCCCTTAAGCCACAGATGGGGAGCTCCCGGGGCTGATGGAG 4304

Chimp 4305 TTCCACGATGTTGATCACTGGAATTGATTCCTCTTGCAGGCAGCTCCTGCAGGGGCACCC 4364

||||||||||||||||||||||||||||||||||||||||||||||||||||||||||||

Human 4305 TTCCACGATGTTGATCACTGGAATTGATTCCTCTTGCAGGCAGCTCCTGCAGGGGCACCC 4364

Chimp 4365 CTCCAGGGAGCTCTGACCAAGCAGACATACTGACGGTCTACTCAGCCGCAGAGGGTAAGG 4424

|||||||||||||||||||||||||||| |||||||||||||||||||||||||||||||

Human 4365 CTCCAGGGAGCTCTGACCAAGCAGACATCCTGACGGTCTACTCAGCCGCAGAGGGTAAGG 4424

Chimp 4425 AGATGGGTCATCGGGAGCCTGTGGTTACACAGGGCCCAGCTTCCTGGCCTAAGATCTGGA 4484

||||||||||||||||||||||||||||||||||||||||||||||||||||||||||||

Human 4425 AGATGGGTCATCGGGAGCCTGTGGTTACACAGGGCCCAGCTTCCTGGCCTAAGATCTGGA 4484

Chimp 4485 GTAGCCTTAGGGGCAGCTAGGGCTTAGGGTTGGGGCACAGAGATGCCAGCCCAGCTGTGG 4544

||||||||||||||||||||||||||||||||||||||||||||||||||||||||||||

Human 4485 GTAGCCTTAGGGGCAGCTAGGGCTTAGGGTTGGGGCACAGAGATGCCAGCCCAGCTGTGG 4544

Chimp 4545 TCCAGCCATGTTCCCTACATGGGTTATGATGTGTAGTAACAGCAGTGATGGTGAGTGCTG 4604

|||||||||||||||||||||||||| |||||||||||||||||||||||||||||||||

Human 4545 TCCAGCCATGTTCCCTACATGGGTTAGGATGTGTAGTAACAGCAGTGATGGTGAGTGCTG 4604

Chimp 4605 TGAGGCGCAGCTGTGCCAACCACTGTGTGTGCAGGTTTCCTCTAGGCTGTGAGTTCCACG 4664

||||||||||||||||||||||||||||||||||||||||||||||||||||||||||||

Human 4605 TGAGGCGCAGCTGTGCCAACCACTGTGTGTGCAGGTTTCCTCTAGGCTGTGAGTTCCACG 4664

Chimp 4665 AGGCCAGGGTAGATCTGCCTGCCCCAAGGCCCTGCTCAGTGTCTGGCACATAGTAGGTGC 4724

||||||||||||| ||||||||||||||||||||||||||||||||||||||||||||||

Human 4665 AGGCCAGGGTAGACCTGCCTGCCCCAAGGCCCTGCTCAGTGTCTGGCACATAGTAGGTGC 4724

Chimp 4725 ACAGTAAATGTTTGTTCAGTAGTGAATCTCTCCATAGGCTCACCTCTGCAAATACCTAGC 4784

||||||||||||||||||||||||||||||||||||||||||||||||||||||||||||

Human 4725 ACAGTAAATGTTTGTTCAGTAGTGAATCTCTCCATAGGCTCACCTCTGCAAATACCTAGC 4784

Chimp 4785 AACAACTTGTTCCAGATGCAAGAAGTCCCTGCTCCCTGCCCTGTTCTCTTGCCTGATTCC 4844

||||||||||||||||||||||||||||||||||||||||||||||||||||||||||||

Human 4785 AACAACTTGTTCCAGATGCAAGAAGTCCCTGCTCCCTGCCCTGTTCTCTTGCCTGATTCC 4844

Chimp 4845 TGGGTCCTGCCTCCTTGTACCCCACCTTCCACCAACAATAGGACCCCTGGGATTGGAAGG 4904

||||||||||||||||||||||||| ||||||||||||||||||||||||||||||||||

Human 4845 TGGGTCCTGCCTCCTTGTACCCCACTTTCCACCAACAATAGGACCCCTGGGATTGGAAGG 4904

Chimp 4905 CAGAGGGTTGGGGCCTTGGTCTGATGCTCTGGCCCTTATCCCCTGACCTAGGCTATGTGG 4964

|||||||||||||||||||||||||||||||||||| |||||||||||||||||||||||

Human 4905 CAGAGGGTTGGGGCCTTGGTCTGATGCTCTGGCCCTGATCCCCTGACCTAGGCTATGTGG 4964

Chimp 4965 CCTATCGCGATGACAAGGGCTCAGACTTTATCCAGACACTGGTGGAGGTCCTCAGAGCCA 5024

||||||||||||||||||||||||||||||||||||||||||||||||||||||||||||

Human 4965 CCTATCGCGATGACAAGGGCTCAGACTTTATCCAGACACTGGTGGAGGTCCTCAGAGCCA 5024

Chimp 5025 GCCCCGGGAGAGACCTTCTGGAGCTGCTGACTGAGGTGTGTTGGGGGGTTCCAGGGTGAC 5084

|||||||||||||||||||||||||||||||||||||||||||||||||||||||||||

Human 5025 ACCCCGGGAGAGACCTTCTGGAGCTGCTGACTGAGGTGTGTTGGGGGGTTCCAGGGTGAC 5084

Chimp 5085 AAGTGGCAAGGAGCTGGGTTTGCCCTTCTCCCCAGCCCTGGTATTCTGATCACCTCCTAT 5144

||||||||||||||||||||||||||||||||||||||||||||||||||||||||||||

Human 5085 AAGTGGCAAGGAGCTGGGTTTGCCCTTCTCCCCAGCCCTGGTATTCTGATCACCTCCTAT 5144

Chimp 5145 GAACTCCATTGGCAAAGGAGGGATCCTCTGCCCTCAATACTACAAGATAACCAAACGCAG 5204

||||||||||||| ||||||||||||||||||||||||||||||||||||||||||||||

Human 5145 GAACTCCATTGGCGAAGGAGGGATCCTCTGCCCTCAATACTACAAGATAACCAAACGCAG 5204

Chimp 5205 GATGGCCGACGCTGCACAGATGCCATCCACTGCAGTTCTTAGTCACACGTACTGCAGTCG 5264

||||||||||||||||||||||||||||||||||||||||||||||||||||||||||||

Human 5205 GATGGCCGACGCTGCACAGATGCCATCCACTGCAGTTCTTAGTCACACGTACTGCAGTCG 5264

Chimp 5265 GGTGGGGAGGAGGACACTGCATGCCATGCGGGGCCACCTGGGCTTGTGCTCAGAGCAGGG 5324

||||||||||||||||||||||| ||||||||||||||||||||||||||||||||||||

Human 5265 GGTGGGGAGGAGGACACTGCATGTCATGCGGGGCCACCTGGGCTTGTGCTCAGAGCAGGG 5324

Chimp 5325 TGAACCTGCAGGGCCAGTGGGAAGCTGGCTTTGTAGTGACAAGAGTGTGAGATGCCCCCT 5384

||||||||||||||||||||||||||||||||||||||||||||| ||||||||||||||

Human 5325 TGAACCTGCAGGGCCAGTGGGAAGCTGGCTTTGTAGTGACAAGAGAGTGAGATGCCCCCT 5384

Chimp 5385 GGTTCCCACGGGCAGATGTGGTTGGTTGGTTTGAATATTTCCAAGGCCTGTCAGGGGGCT 5444

||||||||||||||||||||||||||||||||||||||||||||||||||||||||||||

Human 5385 GGTTCCCACGGGCAGATGTGGTTGGTTGGTTTGAATATTTCCAAGGCCTGTCAGGGGGCT 5444

Chimp 5445 GAAGTCCATTAGGGTGACGACCAGGTGGGGTGCAGCTGGTCTGCTGAGAGGGGACCTACG 5504

|||||||||||||||||||||||||||||||||||||||||||||||||||||||||| |

Human 5445 GAAGTCCATTAGGGTGACGACCAGGTGGGGTGCAGCTGGTCTGCTGAGAGGGGACCTAGG 5504

Chimp 5505 GGGTGGGAGCCTGTCCTGCTGGGTGGGGACATGTCTGGCCAGAGCAGAGGAATTCACCGT 5564

||||||||||||||||||||||||||||||||||||||||||||||||||||||||||||

Human 5505 GGGTGGGAGCCTGTCCTGCTGGGTGGGGACATGTCTGGCCAGAGCAGAGGAATTCACCGT 5564

Chimp 5565 TAGGCCTCTGGAGCTCTGCGAGCCTCAAAGATGTCCAGGCAGTCCTTGAAATTTTAGGCC 5624

||||||||||||||||||||||||||||||||||||||||||||||||||||||||||||

Human 5565 TAGGCCTCTGGAGCTCTGCGAGCCTCAAAGATGTCCAGGCAGTCCTTGAAATTTTAGGCC 5624

Chimp 5625 TTGCAATGTACCAAGCCAGGGGCTCTCCCCTGGGGCAGGCTGAGCCCCGGAGGGCTGTAG 5684

|||||||||||||||||||||||||||||||||||||||||||||||||||||||||||

Human 5625 TTGCAATGTACCAAGCCAGGGGCTCTCCCCTGGGGCAGGCTGAGCCCCGGAGGGCTGTAA 5684

Chimp 5685 CCCCGGGCACAGGCTGGGTGTGGTTCTCAGGTCAACAGGCGGATGTGTGAGCAGGAGGTG 5744

|||||||| ||||||||||||||| ||||||||||||||||| |||| ||||||||||||

Human 5685 CCCCGGGCGCAGGCTGGGTGTGGTCCTCAGGTCAACAGGCGGGTGTGCGAGCAGGAGGTG 5744

Chimp 5745 CTGGGCCCCGACTGCGATGAACTCCGCAAGGCCTGCCTGGAGATCCGCAGCTCGCTCCGG 5804

||||||||||||||||||||||||||||||||||||||||||||||||||||||||||||

Human 5745 CTGGGCCCCGACTGCGATGAACTCCGCAAGGCCTGCCTGGAGATCCGCAGCTCGCTCCGG 5804

Chimp 5805 CGCCGGCTCTGCCTCCAGGCCTGAGGGTGCGGCGGCCACGGGGGCGCTGCTGAGACGGTG 5864

||||||||||||||||||||||||||||||||||||||||||||||||||||||||||||

Human 5805 CGCCGGCTCTGCCTCCAGGCCTGAGGGTGCGGCGGCCACGGGGGCGCTGCTGAGACGGTG 5864

**Supplementary Figure S7. Nucleotide sequence alignment of chimpanzee (chimp) *CASP16* and human *CASP16P*.** The coding sequence of *CASP16* (11 exons) of the chimpanzee (*Pan troglodytes*) is highlighted by red fonts. The homologous sequence of human *CASP16P* is highlighted by blue fonts. A deletion leading to a frameshift in the human sequence is marked by red shading. The nucleotide sequences correspond to GenBank accession numbers NC_072416.2, nucleotides 5813029-5818892 (chimpanzee) and NC_000016.10, nucleotides 3143981-3149844 (human).

**A**

Human (*Homo sapiens*) *CASP16P*


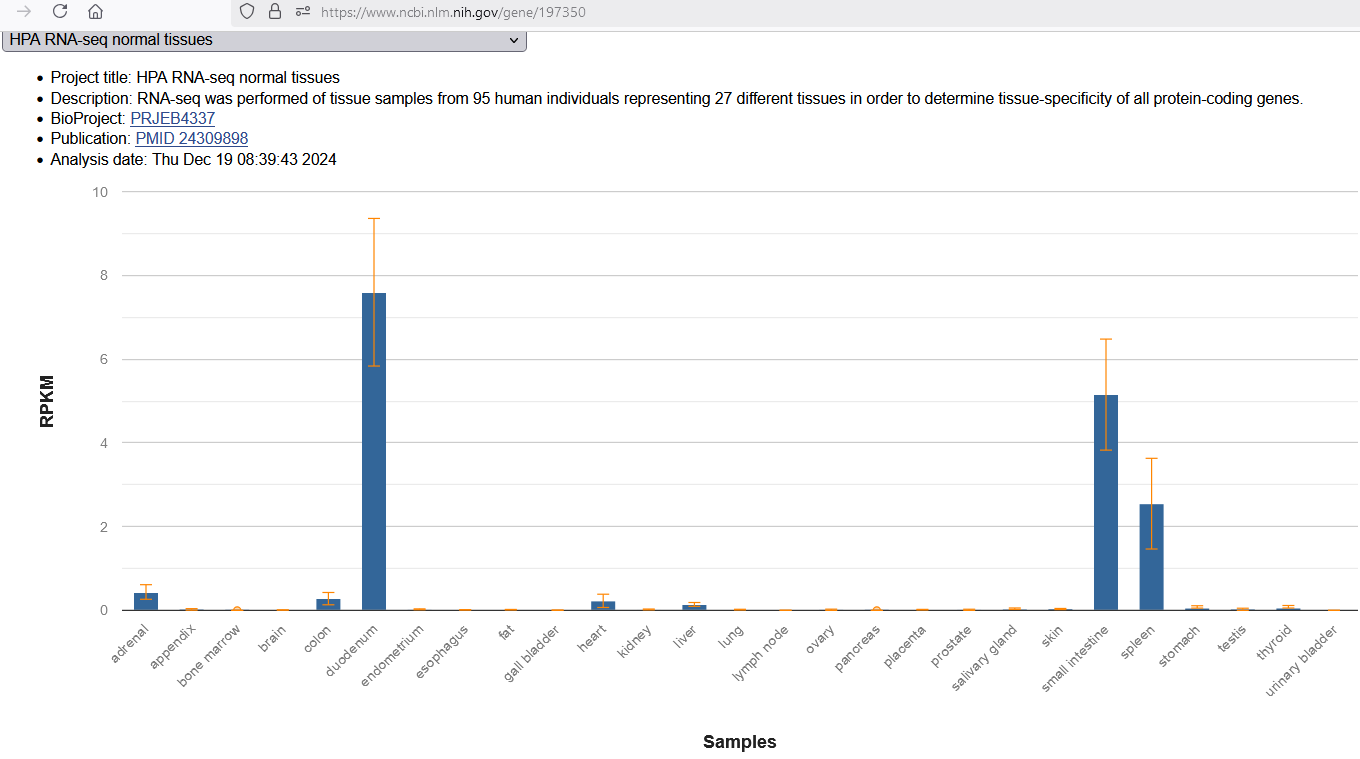


**B**

Cattle (*Bos taurus*) *CASP16*


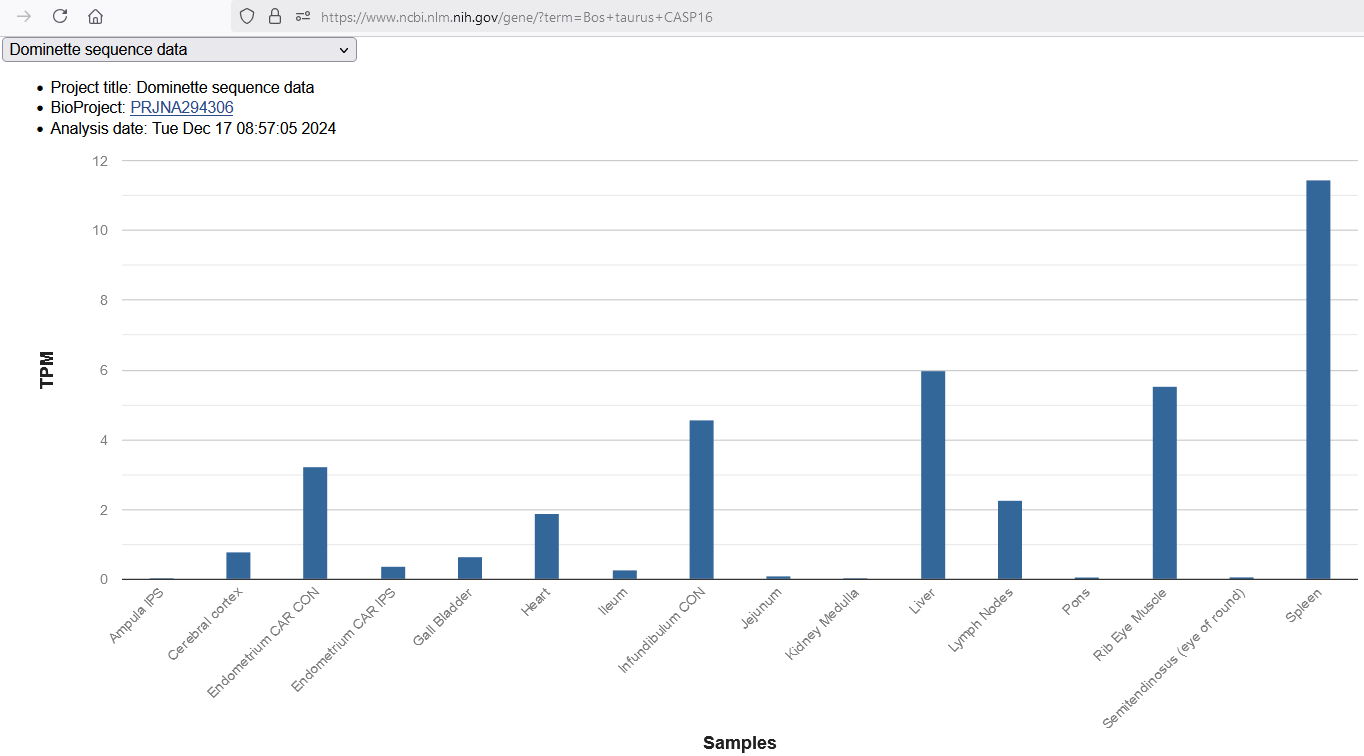


**Supplementary Figure S8. Transcription of human *CASP16P* and bovine *CASP16* mRNAs in tissues. (A)** Abundance of *CASP16P* transcripts in human tissues. **(B)** Abundance of *CASP16* transcripts in bovine tissues. Screenshots from the “Expression” section of NCBI GenBank “Gene” view (https://www.ncbi.nlm.nih.gov/gene/, last accessed on December 22, 2024) show data from Fagerberg et al. Mol Cell Proteomics 2014;13:397-406, doi: 10.1074/mcp.M113.035600 **(A)** and a Bioproject with GenBank accession number PRJNA294306 **(B)**. Abbreviations: RPKM, reads per kilobase per million mapped reads; TPM, transcripts per kilobase million.

**A**


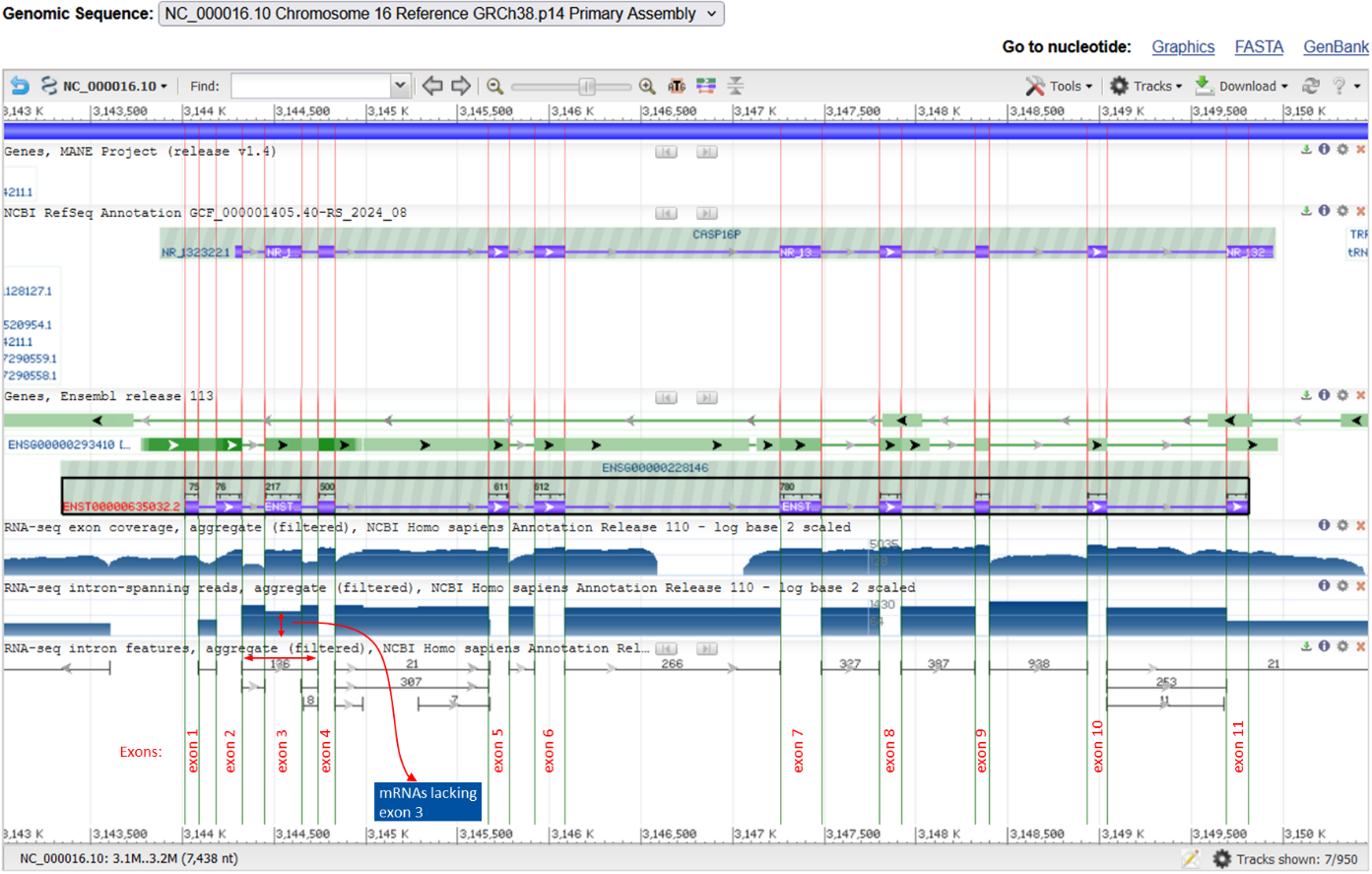


**B**

Translation of human *CASP16P* mRNA containing exon 3, reading frame determined by start codon in exon 1

agcttggtgcttggaggaagccccgtctcgccct**atg**gccttcctggtggctgggaccctg

A W C L E E A P S R P M A F L V A G T L

cggctggccacgcagatggcagacactcgagacagcctggggagaaaggggaagtatgac

R L A T Q M A D T R D S L G R K G K Y D

gtccagggtccaagggcagccctgatgctcagcagccctggggtggcggccgctgtagtc

V Q G P R A A L M L S S P G V A A A V V

actgccctggaggacgtgttccaggccctgggctttgagagctgcgagaggagggaggtc

T A L E D V F Q A L G F E S C E R R E V

ccggtccag**ggcttcctcgaggaactggcttggttccaggagcagctggatgcccacggg**

P V Q G F L E E L A W F Q E Q L D A H G

**cgccctgtggggtgtgccttagtggccttgatgcccccagagggcagctgaggcagccac**

R P V G C A L V A L M P P E G S - G S H

**agcagctggtccgggagctgagcggctgccgggccctgcggggctgccccaaagtcttcc**

S S W S G S - A A A G P C G A A P K S S

**tgctgctctcaagtggtcctgggtcctccctggagcccggagccttccttgctggcctga**

C C S Q V V L G P P W S P E P S L L A -

gagagctgtgtggccgctctcctcactggtccctggtgcagctgctgacgaagctcttcc

E S C V A A L L T G P W C S C - R S S S

gcagggtggctgaagagtccgcagggggcacctgctgccccgtccttcggagctccttga

A G W L K S P Q G A P A A P S F G A P -

ggggggcactgtgcctgggaggcgtggagccctggaggcctgagccggcccccggtccca

G G H C A W E A W S P G G L S R P P V P

gcacacagtatgacctgtccaaggccagggctgccctcctcctggctgtgatccaaggcc

A H S M T C P R P G L P S S W L - S K A

ggcctggggcccagcatgacgtggaggcgctggggggcctgtgctgggccctgggctttg

G L G P S M T W R R W G A C A G P W A L

agaccaccgtgagaacggaccctacagcccaggctttccaggaggagctggcccagttcc

R P P - E R T L Q P R L S R R S W P S S

gggagcaactggacacctgcaggggccctgtgagctgtgcccttgtggccctgatggccc

G S N W T P A G A L - A V P L W P - W P

atgggggaccacggggtcagctgctgggggctgacgggcaagaggtgcagcccgaggcac

M G D H G V S C W G L T G K R C S P R H

tcatgcaggagctgagccgctgccaggtgctgcagggccgccccaagatcttcctgttgc

S C R S - A A A R C C R A A P R S S C C

aggcctgccgtgggggaaacagggatgctggtgtggggcccacagctctcccctggtact

R P A V G E T G M L V W G P Q L S P G T

ggagctggctgcgggcacctccatctgtcccctcccatgcagatgtcctgcagatctacg

G A G C G H L H L S P P M Q M S C R S T

ctgaggcccaaggcagctcctgcaggggcacccctccagggagctctgaccaagcagaca

L R P K A A P A G A P L Q G A L T K Q T

tcctgacggtctactcagccgcagagggctatgtggcctatcgcgatgacaagggctcag

S - R S T Q P Q R A M W P I A M T R A Q

actttatccagacactggtggaggtcctcagagccaaccccgggagagaccttctggagc

T L S R H W W R S S E P T P G E T F W S

tgctgactgaggtcaacaggcgggtgtgcgagcaggaggtgctgggccccgactgcgatg

C - L R S T G G C A S R R C W A P T A M

aactccgcaaggcctgcctggagatccgcagctcgctccggcgccggctctgcctccagg

N S A R P A W R S A A R S G A G S A S R

cctgagggtgcggcggccacgggggcgctgctgagacggtg

P E G A A A T G A L L R R

**C**

Translation of human *CASP16P* mRNA containing exon 3, reading frame for the catalytic domain

agcttggtgcttggaggaagccccgtctcgccctatggccttcctggtggctgggaccct

S L V L G G S P V S P Y G L P G G W D P

gcggctggccacgcagatggcagacactcgagacagcctggggagaaaggggaagtatga

A A G H A D G R H S R Q P G E K G E V -

cgtccagggtccaagggcagccctgatgctcagcagccctggggtggcggccgctgtagt

R P G S K G S P D A Q Q P W G G G R C S

cactgccctggaggacgtgttccaggccctgggctttgagagctgcgagaggagggaggt

H C P G G R V P G P G L - E L R E E G G

cccggtccagggcttcctcgaggaactggcttggttccaggagcagctggatgcccacgg

P G P G L P R G T G L V P G A A G C P R

gcgccctgtggggtgtgccttagtggccttgatgcccccagagggcagctgaggcagcca

A P C G V C L S G L D A P R G Q L R Q P

cagcagctggtccgggagctgagcggctgccgggccctgcggggctgccccaaagtcttc

Q Q L V R E L S G C R A L R G C P K V F

ctgctgctctcaagtggtcctgggtcctccctggagcccggagccttccttgctggcctg

L L L S S G P G S S L E P G A F L A G L

agagagctgtgtggccgctctcctcactggtccctggtgcagctgctgacgaagctcttc

R E L C G R S P H W S L V Q L L T K L F

cgcagggtggctgaagagtccgcagggggcacctgctgccccgtccttcggagctccttg

R R V A E E S A G G T C C P V L R S S L

aggggggcactgtgcctgggaggcgtggagccctggaggcctgagccggcccccggtccc

R G A L C L G G V E P W R P E P A P G P

agcacacagtatgacctgtccaaggccagggctgccctcctcctggctgtgatccaaggc

S T Q Y D L S K A R A A L L L A V I Q G

cggcctggggcccagcatgacgtggaggcgctggggggcctgtgctgggccctgggcttt

R P G A Q H D V E A L G G L C W A L G F

gagaccaccgtgagaacggaccctacagcccaggctttccaggaggagctggcccagttc

E T T V R T D P T A Q A F Q E E L A Q F

cgggagcaactggacacctgcaggggccctgtgagctgtgcccttgtggccctgatggcc

R E Q L D T C R G P V S C A L V A L M A

catgggggaccacggggtcagctgctgggggctgacgggcaagaggtgcagcccgaggca

H G G P R G Q L L G A D G Q E V Q P E A

ctcatgcaggagctgagccgctgccaggtgctgcagggccgccccaagatcttcctgttg

L M Q E L S R C Q V L Q G R P K I F L L

caggcctgccgtgggggaaacagggatgctggtgtggggcccacagctctcccctggtac

Q A C R G G N R D A G V G P T A L P W Y

tggagctggctgcgggcacctccatctgtcccctcccatgcagatgtcctgcagatctac

W S W L R A P P S V P S H A D V L Q I Y

gctgaggcccaaggcagctcctgcaggggcacccctccagggagctctgaccaagcagac

A E A Q G S S C R G T P P G S S D Q A D

atcctgacggtctactcagccgcagagggctatgtggcctatcgcgatgacaagggctca

I L T V Y S A A E G Y V A Y R D D K G S

gactttatccagacactggtggaggtcctcagagccaaccccgggagagaccttctggag

D F I Q T L V E V L R A N P G R D L L E

ctgctgactgaggtcaacaggcgggtgtgcgagcaggaggtgctgggccccgactgcgat

L L T E V N R R V C E Q E V L G P D C D

gaactccgcaaggcctgcctggagatccgcagctcgctccggcgccggctctgcctccag

E L R K A C L E I R S S L R R R L C L Q

gcctgagggtgcggcggccacgggggcgctgctgagacggtg

A - G C G G H G G A A E T V

**D**

Translation of human *CASP16P* mRNA lacking exon 3

agcttggtgcttggaggaagccccgtctcgccct**atg**gccttcctggtggctgggaccctg

A W C L E E A P S R P M A F L V A G T L

cggctggccacgcagatggcagacactcgagacagcctggggagaaaggggaagtatgac

R L A T Q M A D T R D S L G R K G K Y D

gtccagggtccaagggcagccctgatgctcagcagccctggggtggcggccgctgtagtc

V Q G P R A A L M L S S P G V A A A V V

actgccctggaggacgtgttccaggccctgggctttgagagctgcgagaggagggaggtc

T A L E D V F Q A L G F E S C E R R E V

ccggtccag**cctccctggagcccggagccttccttgctggcctgagagagctgtgtggcc**

P V Q P P W S P E P S L L A - E S C V A

**gctctcctcactggtccctggtgcagctgctgacgaag**ctcttccgcagggtggctgaag

A L L T G P W C S C - R S S S A G W L K

agtccgcagggggcacctgctgccccgtccttcggagctccttgaggggggcactgtgcc

S P Q G A P A A P S F G A P - G G H C A

tgggaggcgtggagccctggaggcctgagccggcccccggtcccagcacacagtatgacc

W E A W S P G G L S R P P V P A H S M T

tgtccaaggccagggctgccctcctcctggctgtgatccaaggccggcctggggcccagc

C P R P G L P S S W L - S K A G L G P S

atgacgtggaggcgctggggggcctgtgctgggccctgggctttgagaccaccgtgagaa

M T W R R W G A C A G P W A L R P P - E

cggaccctacagcccaggctttccaggaggagctggcccagttccgggagcaactggaca

R T L Q P R L S R R S W P S S G S N W T

cctgcaggggccctgtgagctgtgcccttgtggccctgatggcccatgggggaccacggg

P A G A L - A V P L W P - W P M G D H G

gtcagctgctgggggctgacgggcaagaggtgcagcccgaggcactcatgcaggagctga

V S C W G L T G K R C S P R H S C R S -

gccgctgccaggtgctgcagggccgccccaagatcttcctgttgcaggcctgccgtgggg

A A A R C C R A A P R S S C C R P A V G

gaaacagggatgctggtgtggggcccacagctctcccctggtactggagctggctgcggg

E T G M L V W G P Q L S P G T G A G C G

cacctccatctgtcccctcccatgcagatgtcctgcagatctacgctgaggcccaaggca

H L H L S P P M Q M S C R S T L R P K A

gctcctgcaggggcacccctccagggagctctgaccaagcagacatcctgacggtctact

A P A G A P L Q G A L T K Q T S - R S T

cagccgcagagggctatgtggcctatcgcgatgacaagggctcagactttatccagacac

Q P Q R A M W P I A M T R A Q T L S R H

tggtggaggtcctcagagccaaccccgggagagaccttctggagctgctgactgaggtca

W W R S S E P T P G E T F W S C - L R S

acaggcgggtgtgcgagcaggaggtgctgggccccgactgcgatgaactccgcaaggcct

T G G C A S R R C W A P T A M N S A R P

gcctggagatccgcagctcgctccggcgccggctctgcctccaggcctgagggtgcggcg

A W R S A A R S G A G S A S R P E G A A

gccacgggggcgctgctgagacggtg

A T G A L L R R

**Supplementary Figure S9. Human *CASP16P* mRNAs do not encode an active protease. (A)** Localization of exons and introns in human *CASP16P*. RNA-seq exon coverage and RNA-seq intron-spanning reads of *CASP16P* are shown on a screenshot of the “Genomic Regions, Transcripts, and Products” section of the “Gene” view of *CASP16P* in NCBI GenBank (https://www.ncbi.nlm.nih.gov/gene/197350). The transcript with the accession number ENST00000635032.2 contains the exons (labelled using red fonts) of *CASP16P* as predicted in Suppl. Fig. S7. RNA-seq exon coverage and RNA-seq intron-spanning reads are depicted as histograms at the indicated positions of human chromosome 16. Note that exon 3 is revealed as a peak in the histogram of exon coverage, but at the same position there are also “intron-spanning reads”, which correspond to RNA-seq reads that directly connect exons 2 and 4. The latter represent mRNAs lacking exon 3. Detailed information about the information displayed at the “Gene” view of NCBI GenBank is provided in Murphy M et al. Gene Help: Integrated Access to Genes of Genomes in the Reference Sequence Collection. 2006 Sep 13 [Updated 2022 Nov 4]. In: Gene Help [Internet]. Bethesda (MD): National Center for Biotechnology Information (US); 2005-. Available from: https://www.ncbi.nlm.nih.gov/books/NBK3841/, last accessed on March 27, 2025). **(B)** Translation of human *CASP16P* mRNA containing exon 3 (red fonts). The reading frame determined by the start codon in exon 1 (green shading) is translated. The amino acid sequence is shown below the nucleotide sequence. Yellow shading, coding sequence. Exon 4, blue fonts. **(C)** Translation of human *CASP16P* mRNA in the reading frame of the catalytic domain. Color labels are the same as in panel B, with the exception of exon 3 and 4, which are not highlighted here. In addition, an in-frame upstream stop codon is shaded red. **(D)** Translation of a human *CASP16P* mRNA lacking exon 3. Color labels are the same as in panel B.


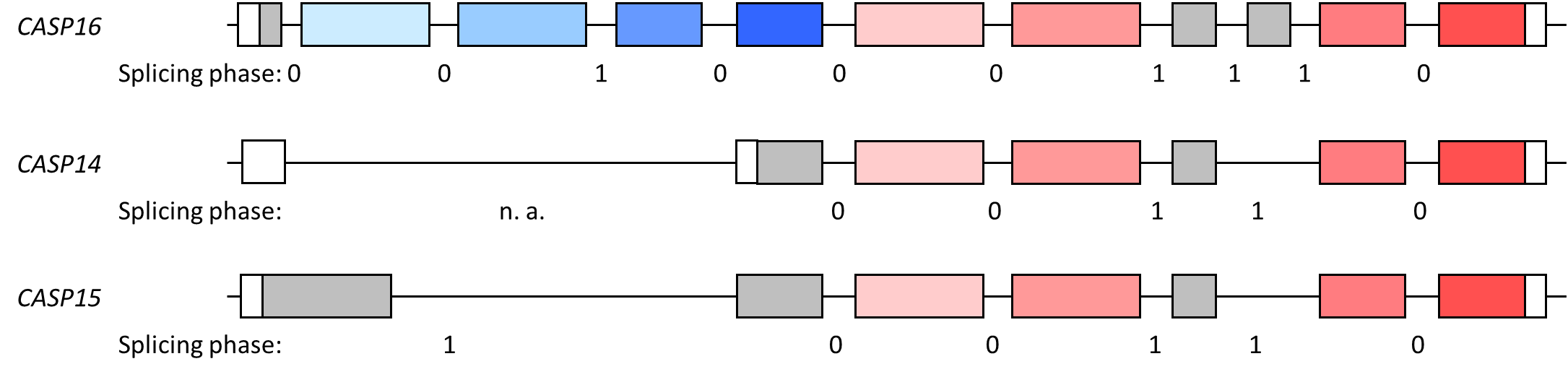


**Supplementary Figure S10. Exon-intron structures of *CASP14*, *CASP15* and *CASP16*.** Exons are depicted as boxes, whereas introns and intergenic sequences are shown as horizontal lines. Note that the length of the boxes and lines are not proportional to the lengths of exons and introns. Red boxes indicate exons that encode the catalytic domain. Blue boxes indicate exons that have arisen by duplication of catalytic domain-encoding exons, as depicted in Figure 3A. Grey boxes indicate other protein-coding segments, and white boxes indicate non-coding regions. The splicing phases are indicated below the introns except for one intron in the 5’-non-coding region of *CASP14*, for which the definition of a splicing phase relative to codons is not applicable (n. a.). The data were obtained by analysis of the following genes: *CASP14 caspase 14* [*Homo sapiens* (human)], Gene ID: 23581, updated on 8-Mar-2025, https://www.ncbi.nlm.nih.gov/gene/23581; *CASP15*, *LOC515736* [*Bos taurus* (domestic cattle)], Gene ID: 515736, updated on 30-Jan-2025, https://www.ncbi.nlm.nih.gov/gene/515736; *CASP16* *caspase 16* [*Pan troglodytes* (chimpanzee)], Gene ID: 467884, updated on 17-Aug-2024, https://www.ncbi.nlm.nih.gov/gene/467884. References: *CASP14*: Eckhart L, Ban J, Fischer H, Tschachler E (2000) Caspase-14: analysis of gene structure and mRNA expression during keratinocyte differentiation. Biochem Biophys Res Commun. 277:655-659. doi: 10.1006/bbrc.2000.3698. *CASP15*: Eckhart L, Uthman A, Sipos W, Tschachler E (2006) Genome sequence comparison reveals independent inactivation of the caspase-15 gene in different evolutionary lineages of mammals. Mol Biol Evol. 23:2081-2089. doi: 10.1093/molbev/msl077. *CASP16*: Suppl. Fig. S1, this study.
